# Supplementary material for: Dissection of gene expression datasets into clinically relevant interaction signatures via high-dimensional correlation maximization
Source: Nat Commun. 2019 Nov 28;10:5417. doi: 10.1038/s41467-019-12713-5 (PMC6883077; doi:10.1038/s41467-019-12713-5)
Supplement: Supplementary file 1 — Supplementary Information [file 41467_2019_12713_MOESM1_ESM.pdf]

Supplementary Information for

Dissection of gene expression datasets into clinically relevant interaction signatures via high-dimensional correlation maximization

Michael Grau[1,2], Georg Lenz[1,2], Peter Lenz[3,4,*]

[1]Department of Medicine A, Albert-Schweitzer Campus 1, University Hospital Münster, 48149 Münster, Germany

[2]Cluster of Excellence EXC 1003, Cells in Motion, University of Münster, 48149 Münster, Germany

[3]Department of Physics, Renthof 5, University of Marburg, 35032 Marburg, Germany

[4]LOEWE Center for Synthetic Microbiology, 35032 Marburg, Germany

Table of Contents

Supplementary Notes on SDCM Subroutines 1
Note 1: Standardized signal and initial weights 1
Note 2: Significance of correlations 2
Note 3: Significance of signal strength 3
Note 4: Default qualification thresholds for signatures 4
Note 5: Presort order and look-ahead scheme for step 1 4
Note 6: Convergence criteria for step 2 5
Note 7: Convergence criteria for step 3 6
Supplementary Notes on Method Comparison and Validation 7
Note 8: Measure for signature comparison and detection performance 7
Note 9: Compared methods 8
Note 10: DLBCL cohort for signature discovery in real gene expression data 8
Note 11: DLBCL cohorts for validation and signature transfer 8
Note 12: Identification of detected signatures 9
Note 13: Method comparison for real data 10
Supplementary Notes on Patient Survival Analyses 11
Note 14: Unbiased survival model selection 11
Note 15: Survival predictor validation 11
Note 16: Comparison with the current standard DLBCL predictor 12
Supplementary Figures 14
Fig. 1: Versatility test, signal dissection by correlation maximization 14
Fig. 2: Versatility test, hierarchical clustering 15
Fig. 3: Versatility test, non-negative matrix factorization 16
Fig. 4: Versatility test, biclustering with FABIA and FABIAS 17
Fig. 5: Versatility test, independent component analysis 19
Fig. 6: Versatility test, principal component analysis 20
Fig. 7: 3D test signal, hierarchical clustering 21
Fig. 8: 3D test signal, biclustering with FABIAS 22
Fig. 9: Versatility test with 13 signatures for performance comparison 23
Fig. 10: Weak signal sensitivity test 24
Fig. 11: Superposition test for signature #3 25
Fig. 12: Superposition test for signature #4 26
Fig. 13: Superposition test for signature #6 27
Fig. 14: Empirical algorithm complexity 28
Fig. 15: Missing values test 29
Fig. 16: Detected gender-associated signature 30
Fig. 17: Rediscovered DLBCL signature Stromal-1 32
Fig. 18: Rediscovered DLBCL signature Stromal-2 33
Fig. 19: Rediscovered cell-of-origin related DLBCL signatures 34
Fig. 20: Overview and characteristics of all 105 detected SDCM signatures 35
Fig. 21: Method comparison for real data, SDCM versus PCA 36
Fig. 22: Method comparison for real data, SDCM versus ICA 37
Fig. 23: Method comparison for real data, SDCM versus FABIA and FABIAS 39
Fig. 24: Method comparison for real data, SDCM versus non-negative matrix factorization 41
Fig. 25: Overview of fitted survival models 43
Fig. 26: Discovered survival signature *k*=27 44
Fig. 27: Discovered survival signature *k*=11 45
Fig. 28: Survival signature based on IC#54 detected by ICA.gauss 46
Fig. 29: Survival predictor performance and generalizability for the tri-variate model 47
Fig. 30: Survival predictor performance and generalizability for the bi-variate model 48
Fig. 31: Generalizability of survival differences between DLBCL subtypes 49
Fig. 32: Predictions within DLBCL subtypes, progression-free survival 50
Fig. 33: Predictions within GCB DLBCL, overall survival 51
Fig. 34: Risk landscape over sample strengths in discovered survival signatures 52
Fig. 35: Predictions within clinical risk classes 53
Fig. 36: Gene set enrichment analyses for SDCM signatures *k*=27and *k*=11 54
Supplementary Tables 55
Table 1: Simulation parameters for signatures in the versatility test scenario 55
Table 2: Performance comparison of SDCM with biclustering methods 56
Table 3: Performance comparison between SDCM and PCA 57
Table 4: Top survival models 58
Supplementary References 59

Supplementary Notes on SDCM Subroutines

While not essential for core detection concepts of SDCM explained in the methods section, we describe subroutines here for completeness.

Note 1. Standardized signal and initial weights

In order to compute specific signature axes, we need gene and sample weights that exclude noise dimensions from signature functional computation. Especially for high-dimensional spaces, where only a few of all measured genes may take part in an interaction, an unweighted computation would wash out scores and prevent detection of small signatures. While final weights are defined more precisely based on correlations (see Methods/Signature focus ($|\mathbf{w}^{\mathrm{g}}\rangle, |\mathbf{w}^{\mathrm{s}}\rangle$)), at the start of each detection iteration k, only the remaining signal $\mathbf{M}_{k-1}$ is available for weights estimation. To get the search strategy started, initial weights $|\mathbf{w}^{\mathrm{s}}_{\mathrm{initial}}\rangle$ respectively $|\mathbf{w}^{\mathrm{g}}_{\mathrm{initial}}\rangle$ are needed as replacement for correlation based weights (see Methods/Step 1). Only for these initial weights, we compute the standardized remaining signal $\mathbf{M}^{\mathcal{S}}_{k-1}$ described in this section at the beginning of each iteration k. Notably, for $k = 1$, SDCM started with the input signal matrix $\mathbf{M}_0$ that may or may not have undergone external data preprocessing, such as $\log_2 -$transformation or median centering of raw gene expression intensities. All $\mathbf{M}_k$ then keep the same unit as $\mathbf{M}_0$, as do all discovered signature matrices $\mathbf{E}_k$.

The standardized remaining signal $\mathbf{M}^{\mathcal{S}}_{k-1}$ is computed via a convergence loop (with index ℓ) from $\mathbf{M}_{k-1}$ as follows. Let $\mathbf{X}$ denote a random variable sampled by all components of a particular gene or sample vector, i.e. by one row or column in the current signal matrix $\mathbf{M}_{k-1,\ell}$. Starting with $\mathbf{M}_{k-1,1} \equiv \mathbf{M}_{k-1}$, we converge the uncentered variances $E[(\mathbf{X}-0)^2]$ for all gene rows and all sample columns to one as follows. First, we compute empirical uncentered variances $|\boldsymbol{v}^{\mathrm{s}}_{\ell}\rangle \in \mathbb{R}^n$ for all samples $|\mathbf{s}_{j,\ell}\rangle$ (columns in $\mathbf{M}_{k-1,\ell}$) via

$$\langle \mathbf{e}^{\mathrm{s}}_j | \boldsymbol{v}^{\mathrm{s}}_{\ell}\rangle \equiv \sum_{i=1}^{m} \langle \mathbf{e}^{\mathrm{g}}_i | \mathbf{s}_{j,\ell}\rangle^2 / m \tag{1}$$

and likewise $|\boldsymbol{v}^{\mathrm{g}}_{\ell}\rangle \in \mathbb{R}^m$ for all genes $|\mathbf{g}_{i,\ell}\rangle$ (rows in $\mathbf{M}_{k-1,\ell}$) via

$$\langle \mathbf{e}^{\mathrm{g}}_i | \boldsymbol{v}^{\mathrm{g}}_{\ell}\rangle \equiv \sum_{j=1}^{n} \langle \mathbf{e}^{\mathrm{s}}_j | \mathbf{g}_{i,\ell}\rangle^2 / n\,. \tag{2}$$

Then, the signal matrix $\mathbf{M}_{k-1,\ell}$ is divided component-wise by the component-wise square roots of the outer product $|\boldsymbol{v}^{\mathrm{g}}_{\ell}\rangle \otimes |\boldsymbol{v}^{\mathrm{s}}_{\ell}\rangle \in \mathbb{R}^{m\times n}$, defining $\mathbf{M}_{k-1,\ell+1}$. These two steps are iterated until uncentered variances of all genes and all samples equal 1 within an epsilon (iteration $\hat{\ell}$). The converged standardized signal $\mathbf{M}^{\mathcal{S}}_{k-1} \equiv \mathbf{M}_{k-1,\hat{\ell}}$ is usually obtained within a few tens of iterations. Notably, this procedure equalizes Euclidean norms for all gene vectors in gene space and for all sample vectors in sample space. Hence, all standardized points are embedded in a sphere within their respective space. In contrast, simply dividing genes by their norms (or samples by theirs) would only equalize norms in either space, but not in both spaces simultaneously.

Standardized components are lowest for gene vectors (or sample vectors) that are expressed at approximately the same level in all sample (or gene) dimensions, which is typical for noise. On the other hand, top standardized components of gene vectors (or sample vectors) are higher if their signal is concentrated in fewer dimensions, i.e. if they have a strong signal only for fewer samples (or fewer genes); this could be an indication for an existing

interaction. We utilize these properties of $\mathbf{M}_{k-1}^{S}$ to define an initial (and rough) approximation of the signature focus. For a gene vector $|\mathbf{g}_i\rangle$, initial sample weights are given by

$$\langle \mathbf{e}_j^{\mathrm{s}} | \mathbf{w}_{\mathrm{initial}}^{\mathrm{s}} \rangle \equiv \min\left(1, \frac{|\mathbf{M}_{k-1}^{S}(i,j)|}{0.8 \cdot \max_{j'}(|\mathbf{M}_{k-1}^{S}(i,j')|)}\right), \quad (3)$$

i.e. by the absolute standardized signal where 80% of the maximum already suffices for full weight. Analogously, for a sample vector $|\mathbf{s}_j\rangle$, initial gene weights are given by

$$\langle \mathbf{e}_i^{\mathrm{g}} | \mathbf{w}_{\mathrm{initial}}^{\mathrm{g}} \rangle \equiv \min\left(1, \frac{|\mathbf{M}_{k-1}^{S}(i,j)|}{0.8 \cdot \max_{i'}(|\mathbf{M}_{k-1}^{S}(i',j)|)}\right) \quad (4)$$

Note 2. Significance of correlations

For low-dimensional spaces or, equivalently, for tight signature foci, it is more probable to obtain high correlations due to noise alone. To decrease the influence of such high but insignificant correlation values, we compute p-values for each correlation and utilize them together with absolute correlation values to define the signature focus (see Methods/Signature focus ($|\mathbf{w}^{\mathrm{g}}\rangle$, $|\mathbf{w}^{\mathrm{s}}\rangle$)).

While it is always possible to estimate these p-values numerically (by simulating the null distribution of r values based on thousands of simulations with random gene expression data), for SDCM we needed a fast-to-compute and deterministic way to estimate these p-values. For a usual (unweighted) Pearson correlation r, the p-value can be estimated by the corresponding t-statistic[1]

$$t = r\sqrt{\nu}/\sqrt{1-r^2}\,, \quad (5)$$

where ν is the number of correlated points minus two, e.g. $n-2$ when correlating two genes in sample space. To approximate corresponding t-statistics for weighted correlations $r = [\mathbf{g}|\mathbf{a}^{\mathrm{s}}]_{|\mathbf{w}^{\mathrm{s}}\rangle}$ of genes with sample axes, we set $\nu^{\mathrm{s}} = \sum_j \langle \mathbf{e}_j^{\mathrm{s}} | \mathbf{w}^{\mathrm{s}} \rangle - 2$. Analogously, for correlations $r = [\mathbf{s}|\mathbf{a}^{\mathrm{g}}]_{|\mathbf{w}^{\mathrm{g}}\rangle}$ of samples with gene axes, we set $\nu^{\mathrm{g}} = \sum_i \langle \mathbf{e}_i^{\mathrm{g}} | \mathbf{w}^{\mathrm{g}} \rangle - 2$. As p-values should quantify the noise probability to observe stronger correlations of the same sign, they are computed by integrating only the respective tail of the t distribution with ν degrees of freedom. I.e., if ρ_ν denotes the probability density function of Student's t-distribution with ν degrees of freedom, we integrate

$$p = \int_t^\infty \rho_\nu(t')dt' \;\text{ for } t \geq 0 \text{ and } p = \int_{-\infty}^t \rho_\nu(t')dt' \;\text{ for } t \leq 0 \quad (6)$$

This approximation has been confirmed to be well defined by p-values based on null distributions, i.e. by computing correlations for normally distributed data.

For signature qualification, we combine all individual p-values. For collected p-values $|\mathbf{p}^{\mathrm{g}}\rangle$ for gene correlations $|\mathbf{r}^{\mathrm{g}}\rangle$ and $|\mathbf{p}^{\mathrm{s}}\rangle$ for $|\mathbf{r}^{\mathrm{s}}\rangle$, a scalar p-value representing all correlations to signature axes is computed as follows. All individual t_i^{g} gene statistics approximately follow a t distribution with ν^{s} degrees of freedom and all t_j^{s} sample statistics follow a t distribution with ν^{g} degrees of freedom (see above). If all observed absolute values $\{|t_i^{\mathrm{g}}| \,|\, i = 1 \ldots m\}$ for genes or $\{|t_j^{\mathrm{s}}| \,|\, j = 1 \ldots n\}$ for samples are significantly higher than absolute values expected by respective t-distributions, then the signature is carried by significant correlations. This is tested by two Kolmogorov-Smirnov tests in the signature focus (using gene weights $|\mathbf{w}^{\mathrm{g}}\rangle$ respectively sample weights $|\mathbf{w}^{\mathrm{s}}\rangle$ when

estimating cumulative distribution functions); they result in $p_{\mathrm{r,genes}}$ for all gene correlations (for their $|t_i^{\mathrm{g}}|$ statistics) and in $p_{\mathrm{r,samples}}$ for all sample correlations. Finally,

$$p_{\mathrm{r}} \equiv \min\left(p_{\mathrm{r,genes}}, p_{\mathrm{r,samples}}\right) \tag{7}$$

provides a scalar p-value for all correlations with current signature axes.

Note 3. Significance of signal strength

The significance of signal strengths should be quantified against the (initially unknown) noise level of the signal. We estimate this level by building an empirical noise distribution $\mathcal{N}_k$. In each detection iteration k, we add gene expression values from the signal matrix $\mathbf{M}_{k-1}$ before dissection that are not correlated to the current signature (dissection strengths $\mathbf{D}_k(i,j) < 10^{-3}$; see Eqn. 14). Values from the signal matrix $\mathbf{M}_k$ after dissection that were strongly correlated to the current signature before dissection (dissection strengths $\mathbf{D}_k(i,j) > 1 - 10^{-3}$) are also added to $\mathcal{N}_k$. Only values from genes and samples that have no strong signal perpendicular to respective signature axes are added ($\leq$ 3 SDs of radial Euclidean distances). Every selected value for gene i and sample j in the noise distribution is assigned $1 - \mathbf{D}_k(i,j)$ as weight. This is repeated in each iteration k, making the estimated noise distribution $\mathcal{N}_k$ larger and more reliable with every dissection. $\mathcal{N}_k$ and weights $\mathbf{W}_{\mathcal{N}_k}$ are stored as matrices of the same size as $\mathbf{M}_k$. Pixels never selected have weight $\mathbf{W}_{\mathcal{N}_k}(i,j) = 0$. Pixels selected as noise estimates in multiple detection iterations k are averaged in $\mathcal{N}_k$ and their maximum weight is retained in $\mathbf{W}_{\mathcal{N}_k}$.

To estimate the significance of all absolute signal values $|\mathbf{M}_{k-1}| \equiv (|\mathbf{M}_{k-1}(i,j)|)_{i,j}$ in a given signature focus, they are compared to absolute signals $|\mathcal{N}_k|$ in the empirical noise distribution. Let $\hat{\mu}\left(|\mathcal{N}_k|, \mathbf{W}_{\mathcal{N}_k}\right)$, $\hat{\sigma}\left(|\mathcal{N}_k|, \mathbf{W}_{\mathcal{N}_k}\right)$ and $\nu_{\mathcal{N}_k} \equiv \sum_{i,j} \mathbf{W}_{\mathcal{N}_k}$ denote the empirical weighted mean, the weighted standard deviation and the weights sum for $|\mathcal{N}_k|$. Let $\mathbf{W}_{\mathrm{focus}}$ denote square roots of components of $|\mathbf{w}^{\mathrm{g}}\rangle \otimes |\mathbf{w}^{\mathrm{s}}\rangle$ for a signature candidate (identical to dissection strengths $\mathbf{D}_k$ for an accepted signature). Then $\hat{\mu}(|\mathbf{M}_{k-1}|, \mathbf{W}_{\mathrm{focus}})$ denotes the weighted mean of absolute signal values in this focus, $\hat{\sigma}(|\mathbf{M}_{k-1}|, \mathbf{W}_{\mathrm{focus}})$ is their weighted standard deviation and $\nu_{\mathbf{W}_{\mathrm{focus}}} \equiv \sum_{i,j} \mathbf{W}_{\mathrm{focus}}$ is the weights sum. With these weighted means, standard deviations and degrees of freedoms, the associated t-statistic is computed identically to usual two-sample t-tests for independent samples with equal variance[1]:

$$\begin{aligned}
\nu &\equiv \nu_{\mathcal{N}_k} + \nu_{\mathbf{W}_{\mathrm{focus}}} - 2 \\
\mathrm{s} &\equiv \sqrt{\left(\left(\nu_{\mathcal{N}_k} - 1\right) \cdot \hat{\sigma}\left(|\mathcal{N}_k|, \mathbf{W}_{\mathcal{N}_k}\right)^2 + \left(\nu_{\mathbf{W}_{\mathrm{focus}}} - 1\right) \cdot \hat{\sigma}(|\mathbf{M}_{k-1}|, \mathbf{W}_{\mathrm{focus}})^2\right) / \nu} \\
t &\equiv \frac{\hat{\mu}(|\mathbf{M}_{k-1}|, \mathbf{W}_{\mathrm{focus}}) - \hat{\mu}\left(|\mathcal{N}_k|, \mathbf{W}_{\mathcal{N}_k}\right)}{\mathrm{s}\sqrt{\dfrac{1}{\nu_{\mathcal{N}_k}} + \dfrac{1}{\nu_{\mathbf{W}_{\mathrm{focus}}}}}}
\end{aligned} \tag{8}$$

We are interested in the probability p_{s} of seeing stronger absolute signal values in the signature focus by pure chance, i.e. we finally integrate

$$p_{\mathrm{s}} = \int_t^{\infty} \rho_\nu(t')dt', \tag{9}$$

where ρ_ν again denotes the probability density function of Student's t-distribution with ν degrees of freedom.

Note 4. Default qualification thresholds for signatures

Several thresholds determine whether a gene or sample is eligible as initial representative for a signature. Based on experience with our test scenarios, we configured the following defaults.

First, the noise probability p_s of signal strengths in the signature focus is tested (cf. significance of signal strength, Supplementary Eqn. 9). We used $\alpha_s = 10^{-5}/(m+n)$ as significance threshold, where the factor $1/(m+n)$ is the Bonferroni correction for multiple hypothesis testing, as we test up to $m+n$ initial representatives. Next, the noise probability p_r of correlations is tested (cf. significance of correlations, Supplementary Eqn. 7). As p_r decreases rapidly over signature size, we were able to choose a very strong significance threshold of $\alpha_r = 10^{-10}/(m+n)$, again using Bonferroni correction.

Statistical significance excludes noise artefacts from being reported as signatures, but cannot filter out statistical true positives that are not of interest because they may represent too weak correlations or may be too small. Therefore, we additionally allow specifying the minimal average correlation in the signature focus (i.e. a threshold for r_k, as defined in Eqn. 23); we chose $r_k \geq 0.4$ as default. Likewise, the minimum number of genes and samples participating in a signature can be specified (as quantified by m_k and n_k in Eqn. 22). With the intention to define defaults that are as general as possible, we utilized formulae that are adaptive to the signal size. The more genes m are measured, the more combinatorial possibilities exist to sort them in a way such that top genes in the sorted signal seem to show a true signature for a few samples. Hence, we should demand more samples supporting a signature for higher numbers of analyzed genes and vice versa. As default thresholds, we demanded $m_k \geq 0.5 \log_2 n$ and $n_k \geq 0.5 \log_2 m$. For a typical real-world signal with $m = 50000$, this results in $n_{\min} \approx 8$ samples that are minimally required to qualify as interesting signature.

Note 5. Presort order and look-ahead scheme for step 1

Instead of testing all genes and all samples as potential first representative of a signature at the beginning of each detection iteration k, we presort them and utilize a look-ahead scheme for performance optimization. In contrast to the correlation-based and thus computationally costly signature functional, presort scores are locally defined, i.e. they can be computed for a single gene or a single sample vector without knowing the signal from others. Hence, they compute relatively fast. Especially for large data sets, processing genes and samples in the order of these scores and using a look-ahead scheme that can break the search loop early provides a substantial performance enhancement of step 1.

The look-ahead scheme is implemented as follows. Once a qualifying axes pair $(|\mathbf{a}^g\rangle, |\mathbf{a}^s\rangle)$ has been found by the search loop (Methods/Step 1), we test if any of the following 200 genes or samples in presort order qualifies with a higher signature functional. If so, we compare it with the next 200 candidates. Otherwise, the search loop stops early without testing any further genes or samples. Notably, should this procedure select an initial representative with a signature functional $\mathcal{E}[|\mathbf{a}^g\rangle, |\mathbf{a}^s\rangle]$ that is lower than the current global maximum, this would only change the signature processing order, as the other signature remains in the signal and is detected in a later iteration.

The presorting scores are based on the maximum of two local information about each gene and sample vector. The first local score is the uncentered standard deviation $\sqrt{\mathbf{E}(\mathbf{X}^2)}$ where $\mathbf{X}$ stands for the random variable sampled by all components of a gene (or sample) vector, i.e. by the respective row (or column) in $\mathbf{M}_{k-1}$. We utilize uncentered

instead of usual standard deviations for the same reason we chose uncentered correlations, i.e. because zero signal values indicate baseline expression and a consistent offset from zero is interesting and should not be ignored (centering would destroy such offset information). The second local score is the gene's (or sample's) maximal component in its absolute standardized signal (defined in the first section of this note), e.g. $\max_j\left(\left|\mathbf{M}_{k-1}^{\mathcal{S}}(i,j)\right|\right)$ for gene $|\mathbf{g}_i\rangle$. This maximal component is a purity score, as it is larger if the signal is concentrated in fewer dimensions, rather than spread equally over many dimensions, which would be more indicative for noise. A purer first representative gene or sample prospectively can achieve a higher average correlation to others, leading to a higher signature functional.

For each gene and each sample, both local scores are computed at the beginning of each detection iteration k. Resulting scores are z-transformed (i.e. centered and divided by their usual standard deviation) to make both sources of local information comparable. Finally, the maximum of these two z scores for each gene and each sample is utilized to presort all genes and all samples in descending order. The resulting combined list of all potential first signature representatives of length $m+n$ is then processed by the search loop as explained in Methods/Step 1, using the look-ahead scheme defined above.

Note 6. Convergence criteria for step 2

Two criteria must be met before signature axes are considered sufficiently reliable and representative.

The first criterion tests for convergence. We consider signature axes converged, if adding another representative gene or sample keeps them correlated with preceding axes > 0.999. More precisely we compute one minus the correlation to the preceding axis, i.e.

$$\delta^{\mathrm{g}} \equiv 1-[\mathbf{b}_l^{\mathrm{g}}|\mathbf{b}_{l+1}^{\mathrm{g}}]_{|\mathbf{w}_{l+1}^{\mathrm{g}}\rangle} \text{ respectively } \delta^{\mathrm{s}} \equiv 1-[\mathbf{b}_l^{\mathrm{s}}|\mathbf{b}_{l+1}^{\mathrm{s}}]_{|\mathbf{w}_{l+1}^{\mathrm{s}}\rangle}. \tag{10}$$

(These correlations are always positive, per construction of accumulated axes; cf. Methods/Step 2) For convergence, we require

$$(\delta^{\mathrm{g}}+\delta^{\mathrm{s}})/2 < 10^{-3} \tag{11}$$

As each additional representative can only change axes with a maximum weight of $1/l$, this always converges.

The second criterion demands a minimum amount of selected representatives. This is important, because if two genes (or samples) with nearly identical signal exist by accident, the convergence criterion might already be reached for $l=2$, but this is usually not yet representative nor robust. By default, we require at least 15 representatives or at least 20% of the estimated signature size, whatever is lower. The signature size (m_k and n_k) is estimated as in Eqn. 22. The absolute threshold of 15 is intended for large signatures as there is no point in adding more representatives and wasting computation resources, if axes have already converged. The relative cut at 20% is important to demand less than 15 representatives in case of very small signatures that might not have this many members (in this case, forcing more representatives despite convergence would make signature axes less specific and less representative).

Once both criteria are met for a certain $\hat{l}$, signature axes $\left(|\mathbf{b}_{\hat{l}}^{\mathrm{g}}\rangle, |\mathbf{b}_{\hat{l}}^{\mathrm{s}}\rangle\right)$ are considered the final linear approximation of the detected underlying interaction and thus are fixed for the remainder of iteration k. They are passed on to

step 3, where they serve as axes over that precise signature curves are monotonically regressed (see Methods/Step 3).

Note 7. Convergence criteria for step 3

The signature signal is considered bi-monotonically converged as soon as its uncentered weighted correlation with the previous iteration is >0.99. More precisely, we correlate all pixels of the current approximation $\mathcal{S}_{\left|\mathbf{u}^{\mathrm{g}}_{k,i}\right\rangle,\left|\mathbf{u}^{\mathrm{s}}_{k,i}\right\rangle}\left(\widetilde{\boldsymbol{\mathcal{M}}}_{i,\hat{\jmath}}\right)$ by iteration i (cf. Eqns. 32 and 36) with the result from the previous iteration $\mathcal{S}_{\left|\mathbf{u}^{\mathrm{g}}_{k,i-1}\right\rangle,\left|\mathbf{u}^{\mathrm{s}}_{k,i-1}\right\rangle}\left(\widetilde{\boldsymbol{\mathcal{M}}}_{i-1,\hat{\jmath}}\right)$, using uncentered weighted correlation between all pixels with square roots of components of $\left|\mathbf{v}^{\mathrm{g}}_{\hat{l}}\right\rangle\otimes\left|\mathbf{v}^{\mathrm{s}}_{\hat{l}}\right\rangle$ as weights (cf. Eqn. 3). As $\hat{\imath}$ denotes the iteration of step 3 convergence, $\mathcal{S}_{\left|\mathbf{u}^{\mathrm{g}}_{k,\hat{\imath}}\right\rangle,\left|\mathbf{u}^{\mathrm{s}}_{k,\hat{\imath}}\right\rangle}\left(\widetilde{\boldsymbol{\mathcal{M}}}_{\hat{\imath},\hat{\jmath}}\right)$ is passed on to the computation of the final signature signal (see Methods/Step 4).

Supplementary Notes on Method Comparison and Validation

Note 8. Measure for signature comparison and detection performance

For method validation and performance assessment, we compared detected signatures for simulated datasets to known true simulated axes. Besides signature axes detected with SDCM, also PCA, ICA, FABIA/S and NNMF results are directly comparable with simulated axes (using PCs, ICs or column vectors of NNMF or FABIA/S loading matrices, respectively). The same measure was utilized for comparing signatures detected for real gene expression data (cf. Note 13).

Generally, this comparison can be performed in either the gene space (comparing with the known gene axis $|\mathbf{a}^{\text{g,sim}}\rangle$ of a simulated signature) or in the sample space (comparing with $|\mathbf{a}^{\text{s,sim}}\rangle$). In either case, we used uncentered weighted correlations (see Eqn. 3) to perform these comparisons via

$$r = \left[\mathbf{a}^{\text{detected}}\middle|\mathbf{a}^{\text{sim}}\right]_{|\mathbf{w}\rangle}, \tag{12}$$

where $|\mathbf{a}^{\text{detected}}\rangle$ is the detected gene or sample axis (respectively the principal component, the independent component, the column vector in the NNMF matrix factor or the column vector in the FABIA(S) loadings matrix).

Comparison weights $|\mathbf{w}\rangle$ are defined as follows. As no comparison method provides gene and sample weights, we utilized a common weight formula for all methods to facilitate fair comparisons (instead of using weights inferred by SDCM). Full weights are assigned to all dimensions with absolute values $|\langle\mathbf{e}_i|\mathbf{a}\rangle|$ that are greater than 50% of the maximum absolute component of either the simulated or the detected axis. Additionally, weights approach zero for dimensions that do take part in neither the simulated nor in the detected signature; formally:

$$\langle\mathbf{e}_i|\mathbf{w}\rangle \equiv \max\left(\min\left(1, \frac{|\langle\mathbf{e}_i|\mathbf{a}^{\text{sim}}\rangle|}{0.5\cdot\max\limits_{i'}|\langle\mathbf{e}_{i'}|\mathbf{a}^{\text{sim}}\rangle|}\right), \min\left(1, \frac{|\langle\mathbf{e}_i|\mathbf{a}^{\text{detected}}\rangle|}{0.5\cdot\max\limits_{i'}|\langle\mathbf{e}_{i'}|\mathbf{a}^{\text{detected}}\rangle|}\right)\right) \tag{13}$$

For visualization, we display correlations for detected vectors (in Supplementary Figs. 1-6) in best matching order. For the fixed order of simulated signatures #1-#7 (that correspond to the 7 columns in depicted correlation matrices), we reorder detected signatures (i.e. rows in depicted correlation matrices) such that the highest correlations of detected with simulated signature axes appear on the diagonal. The same was done for all 13-signature versatility tests (Supplementary Fig. 9).

As SDCM determines the number of signatures in the signal, a row for each detected signature is displayed, including false positive signatures, if any. As PCA and ICA do not determine the number of signatures (instead, they return as many components as the space has dimensions), we provide the number of simulated signatures and display an according number of top components. In case of PCA, they are determined by variances of the PCs. ICA does not provide any intrinsic order of returned ICs; here we display those ICs with the highest correlations to any simulated signature axis. NNMF, FABIA and FABIAS need the number of signatures pre-specified and were given the true number of simulated signatures in advance.

Note 9. Compared methods

We analyzed both simulated signals of the 7-signature versatility test scenario (Supplementary Figs. 2-6) as well as the real gene expression dataset (Supplementary Figs. 21-24) with several external methods. Here, we list their sources and configurations.

In case of PCA and NNMF, we used functions pca and nnmf of the MATLAB® Statistics Toolbox. HCs were computed with the pdist and linkage functions of the same toolbox. In case of ICA[2], we applied the current fastica implementation v2.5. In case of FABIA and FABIAS[3], computations were performed in R (version 3.2.5, R Core Team, 2016, available at: www.r-project.org) using the FABIA R package v2.16.

If methods offered configuration possibilities, we used default settings, where available. In particular, PCA has been set to utilize singular value decomposition internally and NNMF was computed by alternating least squares (cf. MATLAB® documentation). Additionally, we tested several representative configurations for settings without clear defaults. In case of HC, we tested both the Euclidean and a correlation-based distance (as implemented by pdist; Supplementary Fig. 2). As NNMF does not support negative $\log_2(\text{ratios})$, we applied three different transformations to make the input signal positive. Namely, $\exp(\log_2(\text{ratios}))$, $\log_2(\text{ratios})$ minus their minimum and setting negative $\log_2(\text{ratios})$ to zero (Supplementary Fig. 3). In case of FABIA and FABIAS, we tested each with two previously published configurations (Supplementary Fig. 4). In case of ICA, we tested each of the four implemented nonlinear contrast functions scoring non-normality (namely tanh, gauss, pow3 and skew; defined in[2]; results in Supplementary Fig. 5).

Note 10. DLBCL cohort for signature discovery in real gene expression data

We selected the largest available gene expression cohort of human DLBCL samples at time of this study for dissection and signature discovery.

These data are available via NCBI GEO[4], accession GSE31312[5] ($n = 498$ patients). All patients were treated with the current standard of care rituximab and CHOP chemotherapy (R-CHOP). Gene expression in corresponding 498 tumor samples from these patients (biopsied before start of therapy) has been measured for 54675 transcript clusters (Affymetrix GeneChip® HG U133 Plus 2.0 microarrays). To retain maximal genomic resolution, we performed signal dissection on this transcript cluster level (i.e. $m = 54675$). We only aggregated on gene level, when needed for gene set enrichment analysis (GSEA)[6]. For each transcript cluster, measured $\log_2(\text{intensities})$ were initially transformed to $\log_2(\text{ratios})$ relative to cohort mean expression values.

SDCM returned 105 signatures for this detection cohort (general characteristics depicted in Supplementary Fig. 20). All detected signature axes, weights, correlations and strengths are provided in Supplementary Data 1 and 2. Derived traditional gene signatures (i.e. flat sets of top genes) are also provided there for backwards-compatibility with, e.g., GSEA.

Note 11. DLBCL cohorts for validation and signature transfer

Besides GSE31312, we imported two additional gene expression datasets from independent DLBCL patients for validation purposes, one microarray-based and a newer RNA-sequencing based dataset to test robustness of signatures against crossing technological borders.

Microarray-based data are available via NCBI GEO[4], accession GSE10846[7] ($n^{\mathrm{Val}} = 233$ samples from patients who were also treated with R-CHOP; measured by the same Affymetrix GeneChip® HG U133 Plus 2.0 microarray platform with $m^{\mathrm{Val}} = 54675$ probed transcript clusters). RNA-sequencing based data are available from the European Genome-Phenome Archive (https://www.ega-archive.org) via accession EGAS00001002606[8] ($n^{\mathrm{Val}_{\mathrm{RNA-seq}}} = 624$ in the Reddy et. al. RNA-sequencing core set out of 775 samples in total, all samples are from patients treated with R-CHOP). This cohort was not published at time of method development and signal dissection, but became available in time for method, signature and predictor validation. We aligned RNA-sequencing reads against the coding hg38 RefSeq[9] transcriptome (downloaded from ftp://ftp.ncbi.nlm.nih.gov/genomes/refseq/vertebrate_mammalian/Homo_sapiens/reference/GCF_000001405.38_GRCh38.p12/GCF_000001405.38_GRCh38.p12_genomic.gff.gz) using Bowtie[10] and aggregated reads for transcripts and genes with RSEM[11]. Heatmaps show normalized TPM values ($\log_2$(ratios) relative to gene-wise cohort medians).

To facilitate validation, we transferred detected signatures. Like computing sample strengths in the detection cohort (see Eqn. 28), we projected samples $\left|\mathbf{s}_j^{\mathrm{Val}}\right\rangle$ of each validation cohort (defined by columns of the validation signal matrix $\mathbf{M}^{\mathrm{Val}}$) on gene axes $|\mathbf{a}^{\mathrm{g}}\rangle$ detected by SDCM or, e.g., on ICs detected by ICA. I.e., we defined sample strengths $\left|\mathbf{u}^{\mathrm{Val,s}}\right\rangle$ in each validation cohort by

$$\left\langle \mathbf{e}_j^{\mathrm{Val,s}} \middle| \mathbf{u}^{\mathrm{Val,s}} \right\rangle \equiv \left\langle \mathbf{s}_j^{\mathrm{Val}} \middle| \mathbf{a}^{\mathrm{g}} \right\rangle^{0}_{|\mathbf{w}^{\mathrm{g}}\rangle} / \|\mathbf{w}^{\mathrm{g}}\| \,, \qquad (14)$$

where $|\mathbf{a}^{\mathrm{g}}\rangle$ and $|\mathbf{w}^{\mathrm{g}}\rangle$ denote the detected gene axis and gene weights of the signature to transfer. These sample strengths $\left|\mathbf{u}^{\mathrm{Val,s}}\right\rangle$ are the basis for sample ordering, classification and survival prediction in each validation cohort.

As the chosen first validation cohort has been measured with the identical microarray platform, we performed this signature transfer with high genomic resolution on transcript cluster level (i.e., $m^{\mathrm{Val}} = 54675 = m$). For the RNA-sequencing based second validation cohort, this signature transfer was performed with the same projection formula after mapping microarray probe sets to aggregated RNA-sequencing results on gene level. Probe sets without a match (e.g. for older gene models or non-coding RNA sequences) were assigned NaN values (not a number; shown as gray lines in signature heatmaps for the RNA-sequencing cohort).

Note 12. Identification of detected signatures

Generally, signatures can be identified either by associating their sample strengths with known clinical covariates or by associating their gene strengths with known gene signatures.

For the former, we used χ^2 independence tests for categorical data like patient gender and, for follow-up data, Kaplan-Meier survival estimates and log-rank tests. For the latter, we used GSEA[6]. All GSEAs have been performed on gene level; enrichment was computed with respect to gene ranks by transcript cluster strengths $|\mathbf{u}^{\mathrm{g}}\rangle$ in the respective signature. (In case of multiple measured transcript clusters per gene, we selected the one with highest absolute value in $|\mathbf{u}^{\mathrm{g}}\rangle$.) Enrichment significance was assessed by permutation tests. Detailed enrichment results are shown in Supplementary Figs. 17-19.

Note 13. Method comparison for real data

As for simulated versatility tests, we applied PCA, NNMF (three configurations as in Supplementary Fig. 3), FABIA/S (four configurations as in Supplementary Fig. 4) and ICA (four configurations as in Supplementary Fig. 5) also to the real gene expression data of the detection cohort (Note 9).

We compared detected gene axes (respectively PCs, ICs, NNMF or FABIA/S loading vectors) with all SDCM signatures, using the identical measure as for comparison with simulated axes (Note 8). To demonstrate that SDCM is not based on any orthogonality assumption, we first correlated SDCM gene axes with each other to show their partial correlations (Supplementary Fig. 21a).

PCA compressed the signal into few PCs of strong variance (Supplementary Fig. 21b-c), but the gender-associated signature was split over multiple PCs and PCA did not discover any of the key survival signatures with high correlation. Overall, ICA results showed the highest similarity to SDCM (Supplementary Fig. 22); all ICA contrast functions rediscovered the strong gender-associated signature and the large Stromal-1 signature with high correlations, but correlations to identified key survival signatures were only moderate. FABIA/S results showed strong volatility across different configurations. The best FABIAS configuration rediscovered some key signatures, but represented other SDCM signatures multiple times (incomplete dissection; Supplementary Fig. 23). NNMF also represented some SDCM signatures multiple times, but less so than FABIA/S. Its best configuration robustly redetected the gender-associated signature and Stromal-1, like ICA (Supplementary Fig. 24). None of the compared methods and configurations rediscovered both top survival signatures presented in the main text with high correlation.

To determine methods in an unbiased manner that describe the highest impact on patient survival, all signatures detected by either SDCM or any of the comparison methods were subsequently used as basis for survival model construction (Note 14).

Supplementary Notes on Patient Survival Analyses

Note 14. Unbiased survival model selection

While, in case of simulated data, known definitions of simulated patterns support a direct ranking of method performance, true signatures in real gene expression data are mostly unknown. In addition to comparing detection performance for the few biologically known key signatures (cf. Note 13), we therefore used patient survival data as additional independent measure for detection quality. I.e., we ranked methods by the predictive impact of their discovered signatures, assuming that more predictive signatures are also more biologically specific models of underlying interactions.

We associated signatures discovered by SDCM, PCA, ICA, NNMF and FABIA/S with progression-free survival (PFS) in the detection cohort by constructing Cox proportional hazards models[1,12] (CPHMs). Follow-up information was available for 470/498 patients.

To test the predictive power of a detected signature gene axis (or PC, IC, NNMF or FABIA/S loadings vector) $|\mathbf{a}^{\mathrm{g}}\rangle$, we formed explanatory variables for CPHMs based on sample strengths $\langle \mathbf{s}|\mathbf{a}^{\mathrm{g}}\rangle_{|\mathbf{w}^{\mathrm{g}}\rangle}$ for all 470 available samples (i.e. projections of column vectors $|\mathbf{s}\rangle$ in the initial signal matrix $\mathbf{M}_0$ of the detection cohort). To facilitate a fair model selection and because comparison methods do not provide their own gene weights, we used the same common weighting formula as for performance measurement (Supplementary Eqn. 13) for all methods including SDCM, i.e.

$$\langle \mathbf{e}_i^{\mathrm{g}} | \mathbf{w}^{\mathrm{g}} \rangle \equiv \min\left(1, \frac{|\langle \mathbf{e}_i | \mathbf{a}^{\mathrm{g}} \rangle|}{0.5 \cdot \max_{i'} |\langle \mathbf{e}_{i'} | \mathbf{a}^{\mathrm{g}} \rangle|}\right) \qquad (15)$$

Resulting sample strengths were further z-transformed for direct comparability of relative influence of fitted Cox β coefficients. All Cox coefficients, model likelihoods and p_{CPHM}-values for explanatory variables have been computed with the coxphfit function of the MATLAB® Statistics toolbox.

The number of possible combinations n_C of l signatures from a pool of L signatures is $n_C = \binom{L}{l}$. For the 514/3278 signatures concerning at least one third of all samples (cf. Supplementary Fig. 25), we tested $\binom{514}{2} = 131{,}841$ 2-signature CPH models and $\binom{514}{3} = 22{,}500{,}864$ 3-signature CPH models. To rank models, we computed their Akaike information criteria (13, §2.2) (AIC) based on the maximum likelihood estimate of each fitted model and using the aicbic function of the MATLAB® Statistics toolbox.

Top bivariate and top tri-variate models are listed in Supplementary Tables 4a and 4b. The globally best 2-signature model was comprised of signatures {SDCM#11, SDCM#27}, with a relative likelihood (13, §2.8) of 0.02 to the second best bi-variate model. The globally best 3-signature model was comprised of signatures {SDCM#11, SDCM#27, ICA.gauss#54}, with a relative likelihood (13, §2.8) of 0.17 to the second best tri-variate model.

Note 15. Survival predictor validation

Validation of the predictor was performed on two levels. First, we investigated whether discovered signatures also exist on gene expression level in the independent DLBCL cohort. Next, we analyzed whether these signatures are also predictive on survival level in the independent validation cohort.

We evaluated both the best 3-variate and the best 2-variate survival model from Note 14. First, we validated survival signatures SDCM#27, SDCM#11 and ICA.gauss#54 individually on gene expression level by transferring them to the validation cohort (cf. Note 11 and Supplementary Figs. 26b, 27b and 28b for resulting sample strengths and correspondingly sorted samples in the validation cohort). Correlations between most top genes of either signature were retained in this independent cohort, in this way validating the existence of these gene expression signatures in DLBCL (not just cohort-specific laboratory effects).

Next, we applied both top CPHMs to sample strengths and to progression free survival data for the validation cohort. We collected these strengths for all samples in the validation cohort as vectors $|\mathbf{u}_{\mathrm{SDCM},k=27}^{\mathrm{Val,s}}\rangle$, $|\mathbf{u}_{\mathrm{SDCM},k=11}^{\mathrm{Val,s}}\rangle$ and $|\mathbf{u}_{\mathrm{ICA.gauss},k=54}^{\mathrm{Val,s}}\rangle$, respectively. Then, we applied the best bi-variate or best tri-variate Cox model to predict hazard ratios (aka risk ratios) relative to baseline risk for all sample vectors $|\mathbf{M}^{\mathrm{Val}}(\mathbf{I}_0, j)\rangle$ in the validation cohort. Formally for the bi-variate model,

$$\log(\text{hazard ratio for sample index } j) = \beta_{\mathrm{CPHM},k=27}\langle \mathbf{e}_j^{\mathrm{Val,s}} | \mathbf{u}_{k=27}^{\mathrm{Val,s}}\rangle + \beta_{\mathrm{CPHM},k=11}\langle \mathbf{e}_j^{\mathrm{Val,s}} | \mathbf{u}_{k=11}^{\mathrm{Val,s}}\rangle. \quad (16)$$

To quantify and control predicted survival differences, we computed Kaplan-Meier estimates in both cohorts for identical risk intervals (see Supplementary Fig. 29 and Supplementary Fig. 30). While both models revealed significant survival differences in the validation cohort, generalization properties of the tri-variate model were inferior when compared to the simpler bi-variate model for identical risk cutoffs (compare Supplementary Fig. 29b and Supplementary Fig. 30b). Hence, we considered it over-fitted and selected the simpler bi-variate model for subsequent survival analyses regarding association with DLBCL subtype, comparison with a previous DLBCL predictor and association with patient IPI scores[14].

As the selected final survival model was comprised solely of SDCM signatures, we used the possibility of refitting it with sample strengths based on SDCM gene weights for increased specificity (instead of using the common weights formula needed in Note 14 to facilitate a fair method comparison). The final coefficients of the bi-variate CPHM fitted in the detection cohort are as follows: For standardized sample strengths in signature $k = 27$, a negative Cox coefficient $\beta_{\mathrm{CPHM},k=27} = -0.655$ was fitted with $p_{\mathrm{CPHM},k=27} = 2.1 \cdot 10^{-11}$. Thus, higher sample strengths in signature $k = 27$ are associated with lower hazards and more favorable survival. For standardized sample strengths in signature $k = 11$, a positive Cox coefficient $\beta_{\mathrm{CPHM},k=11} = 0.444$ with $p_{\mathrm{CPHM},k=11} = 2.2 \cdot 10^{-9}$ was fitted. This indicates that higher sample strengths in signature $k = 11$ are associated with higher hazards and inferior survival.

Note 16. Comparison with the current standard DLBCL predictor

Lastly, we compared our final 2-signature model with a previously described 3-signature DLBCL survival predictor[7] based on gene signatures termed germinal center B cell signature, stromal-1 and stromal-2 (rediscovered in Supplementary Figs. 17-19).

For a precise computation of the predictor score formula from 2008, we directly averaged published sets of Affymetrix transcript clusters (see Suppl. Table 3 in[7]), rather than using our rediscovered counterparts. These predictor scores were then used as an additional explanatory variable in CPHMs. We quantified improvements in predictive model capabilities due to added explanatory variables by likelihood ratio tests (LRT) that compare the

larger model with the nested model before adding variables. Let l_2 be the log-likelihood of the larger model and l_1 be the log-likelihood of the smaller nested model. Then $-2(l_1 - l_2)$ is asymptotically χ^2-distributed with one degree of freedom according to Wilks' theorem[15]. Hence, p_{LRT}-values can be readily approximated by integrating the respective upper tail of the χ^2 distribution. For maximal sensitivity, we performed these tests using all available R-CHOP PFS events from both cohorts.

As a self-control, we initially tested the previously described 3-signature score on top of age alone. As expected, it was found to be significant ($p_{\mathrm{LRT}} = 2.4 \cdot 10^{-13}$). Testing our model (i.e. signatures $k = 27$ and $k = 11$) for the same events yielded $p_{\mathrm{LRT}} = 9.5 \cdot 10^{-20}$. Consequently, testing signatures $k = 27$ and $k = 11$ as additional predictor variables on top of the previously published 3-signature predictor score still added significant explanatory value ($p_{\mathrm{LRT}} = 1.3 \cdot 10^{-8}$). Testing vice versa, i.e. adding the former predictor score as an additional explanatory variable to our 2-signature model with signatures $k = 27$ and $k = 11$, did not lead to any significant improvement ($p_{\mathrm{LRT}} = 0.07$). Collectively, our model described all survival dependencies that were captured by the former 3-signature model, plus significant additional dependencies, although it uses one signature less. This indicates a higher specificity of discovered signatures (and their top genes) for biological interactions that are relevant for therapy outcome.

Supplementary Figures

Fig. 1: Versatility test, signal dissection by correlation maximization

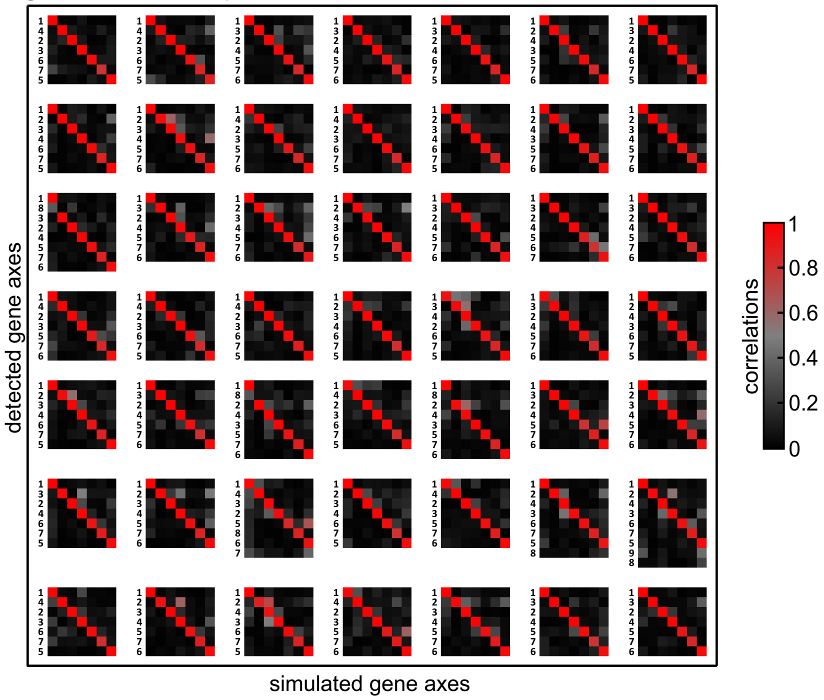

SDCM results for 49 simulations of the 7-signature versatility scenario (cf. Fig. 3a). Depicted are all detected gene axes returned by SDCM, including false positives, if any (numbers indicate detection iterations k). All seven signatures were detected reliably with high sensitivity (each column has a red pixel) and with high specificity (only one red pixel per column). Only rarely, signatures #2 and #3 were not dissected completely (suboptimal specificity, as there is an additional red pixel in the same column). In 5/49 runs SDCM returned false positives, i.e. gene axes that were not correlated with any simulated signature axis. (We can display false positives only for SDCM, as PCA and ICA do not have any built-in detection of the number of signatures and the other methods require this number to be pre-specified.)

Fig. 2: Versatility test, hierarchical clustering

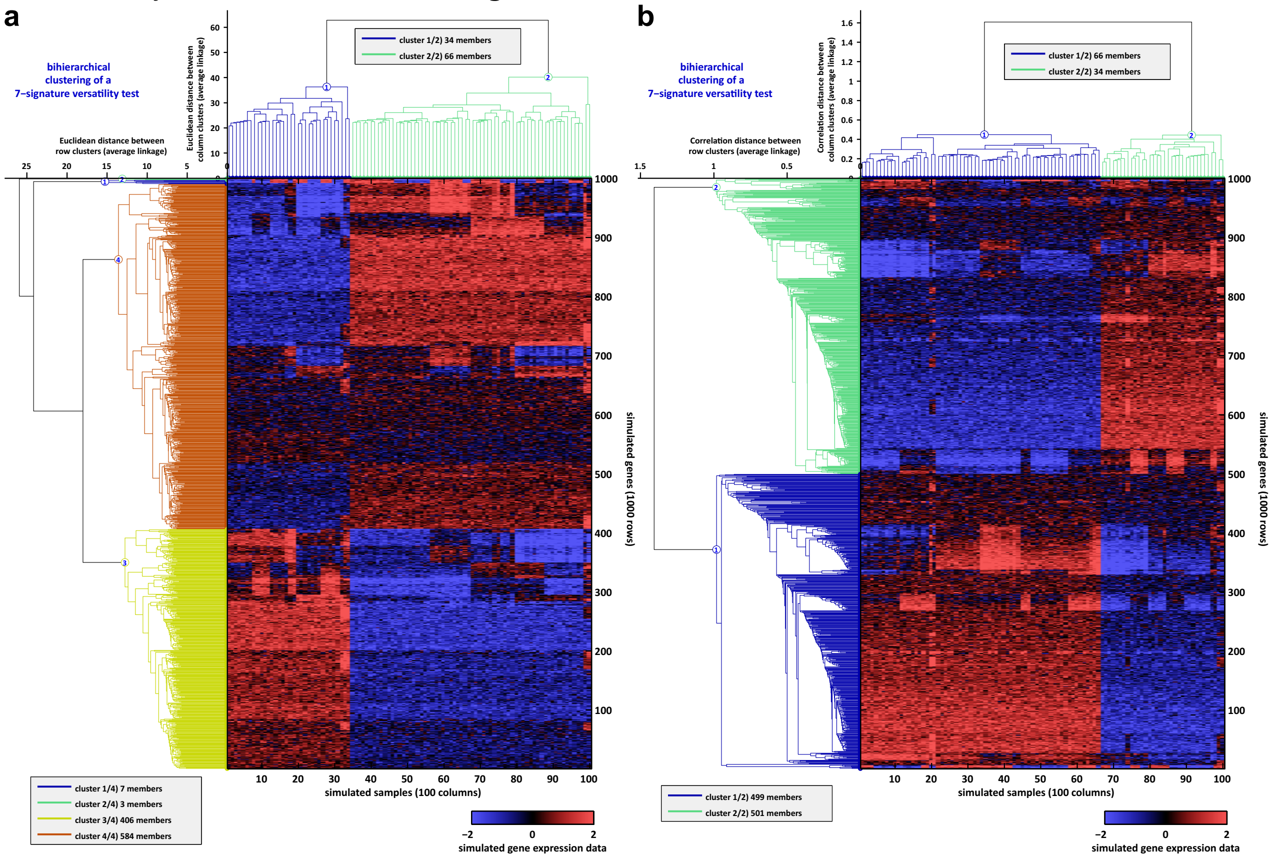

HC of 1000 simulated genes and 100 simulated samples for a 7-signature versatility test (cf. Fig. 3a). **(a)** HC results for the default configuration (Euclidean distance and average linking). HC detected and roughly clustered the strong and large superposed signature #1. Gene cluster 3 (yellow) contains most correlated genes of signature #1 (red on the left, blue on the right), and gene cluster 4 (orange) contains most anti-correlated genes of this signature, although it contains several weakly correlated genes as well. All other superposed signatures #2-#7 were split by these dominating clusters (and by other sub clusters). I.e., HC could not dissect signatures #2-#7 from the superposed signal of signature #1; neither could it reconstruct the order of their participating genes and affected samples. **(b)** HC of the same data as in (a), using the correlation based distance (as implemented by the pdist function in MATLAB®). Here, gene clusters for both halves of signature #1 were detected more clearly than with Euclidean distance. However, all other superposed signatures #2-#7 were still not reconstructed, as HC just arranges genes and samples once, but cannot dissect overlapping signals. (See also Supplementary Fig. 7 for an illustration of HC detection problems in 3D.)

Fig. 3: Versatility test, non-negative matrix factorization

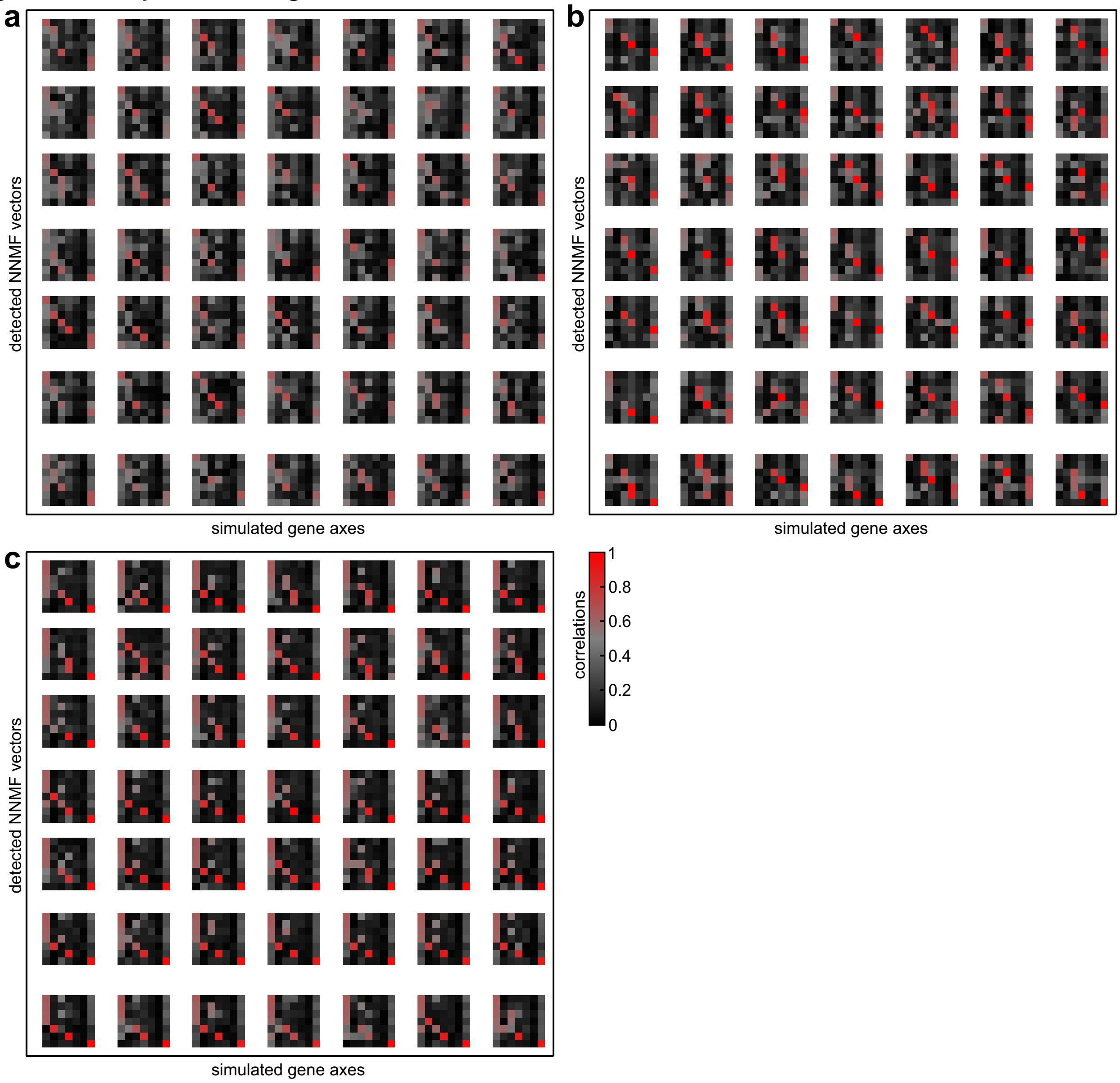

NNMF results for 49 simulations of the 7-signature versatility scenario (cf. Fig. 3a). **(a)** To transform simulated data to positive numbers, as required by NNMF, we used here exp(simulated $\log_2$(ratios)). The true number of signatures in the signal (seven) was provided, as the method requires this input. Simulated gene axes were compared (Supplementary Note 8) with NNMF vectors in gene space (i.e. column vectors of the first NNMF matrix factor, as returned by the nnmf function in MATLAB®). Only rarely, a signature was detected (few red pixels, low sensitivity) and, often, NNMF vectors represented mixtures of several signatures (gray pixels, low specificity). **(b)** NNMF results using simulated $\log_2$(ratios) minus their minimum to transform simulated data to positive numbers. Here, signature #4 was detected in most runs (high sensitivity for this signature), but not always with high specificity (other gray or even red pixels exist in column 4). Sensitivity and specificity were still low for most other signatures. **(c)** NNMF results after zeroing negative $\log_2$(ratios) to transform simulated data to positive numbers as required by NNMF. Here, signature #7 was detected in most runs with high specificity (a single red pixel in column 7). Signatures #5 and #6 were not detected and the signal of signature #1 was split over several detected NNMF vectors.

Fig. 4: Versatility test, biclustering with FABIA and FABIAS

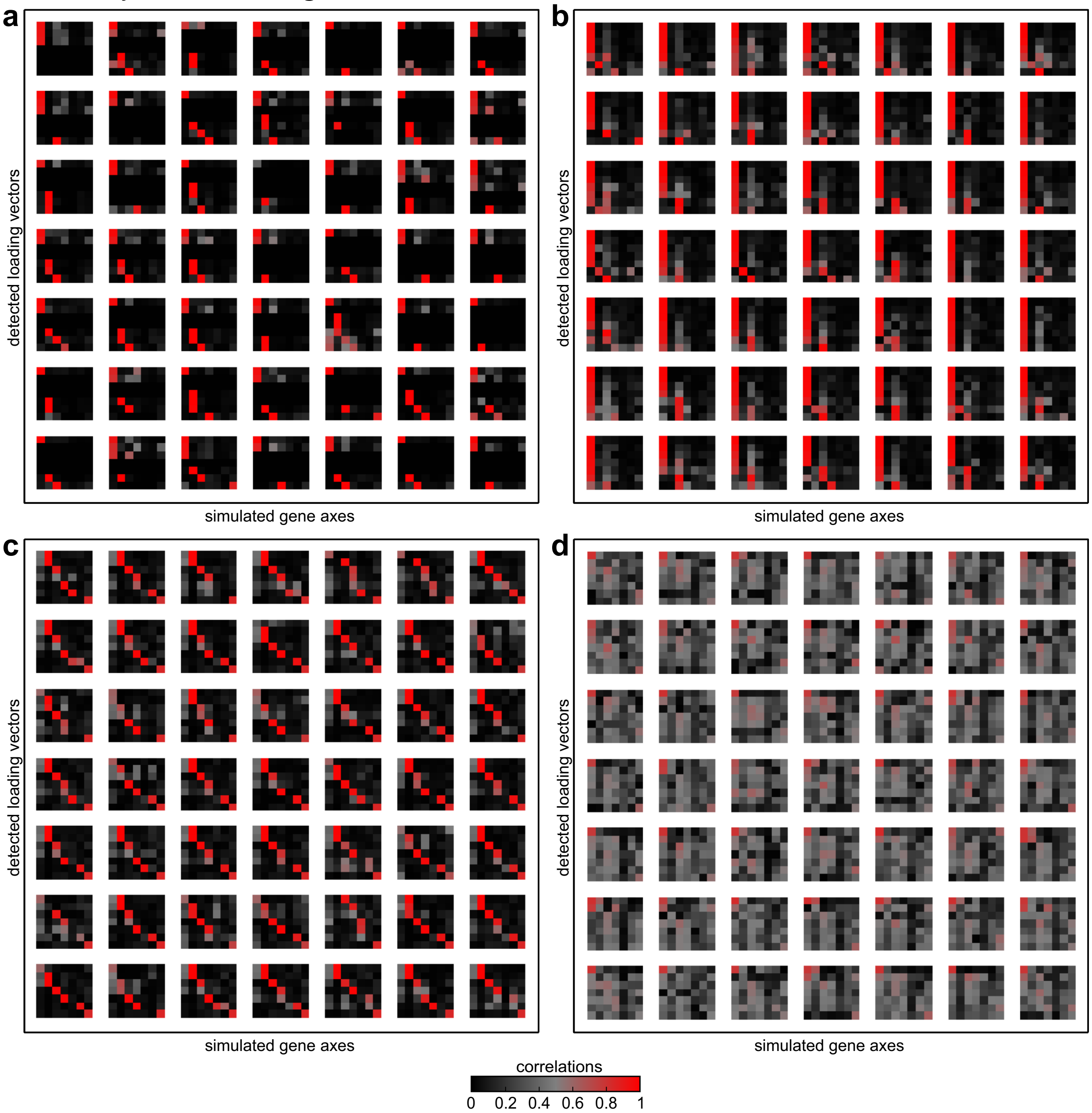

FABIA and FABIAS results for 49 simulations of the 7-signature versatility scenario (cf. Fig. 3a). **(a)** Results for FABIA using original default parameters[33] ($\alpha = 0.4$, cyc $= 200$, spl $= 1$, spz $= 1$). Detected column vectors in FABIA loading matrices were compared with simulated gene axes (Supplementary Note 8). As FABIA needs the number of biclusters in advance, it was given the information that there are seven simulated signatures, as for NNMF. Signature #1 was always detected with high sensitivity (a red pixel exists in each first column), but often its signal was split over more than one detected bicluster (secondary red or gray pixels exist in the first column). Signature #2 was also detected often, but again was sometimes split over multiple biclusters. Signatures #4-#7 were only rarely detected. **(b)** FABIA results for defaults according to its R package v2.16 ($\alpha = 0.01$, cyc $= 500$, spl $= 0$, spz $= 0.5$). Results are dominated by detections of parts of signature #1 whose signal is split over most detected biclusters. **(c)** Results from FABIAS using original default parameters[33] ($\alpha = 0.6$, cyc $= 200$, spz $= 1$). Signatures #2,#3,#4 and #7 were detected most of the time and signatures #5 and #6 several times. Signature #2 was often split over two biclusters and signature #1 was not detected with these settings. Overall, these were the

best biclustering results by FABIA/FABIAS for our versatility test. **(d)** FABIAS results for defaults according to its R package v2.16 ($\alpha = 0.1$, $\mathrm{cyc} = 500$, $\mathrm{spz} = 0.5$). Here, signals of simulated signatures were split over almost all detected biclusters and each bicluster represented signal parts of all simulated signatures (gray pixels).

Fig. 5: Versatility test, independent component analysis

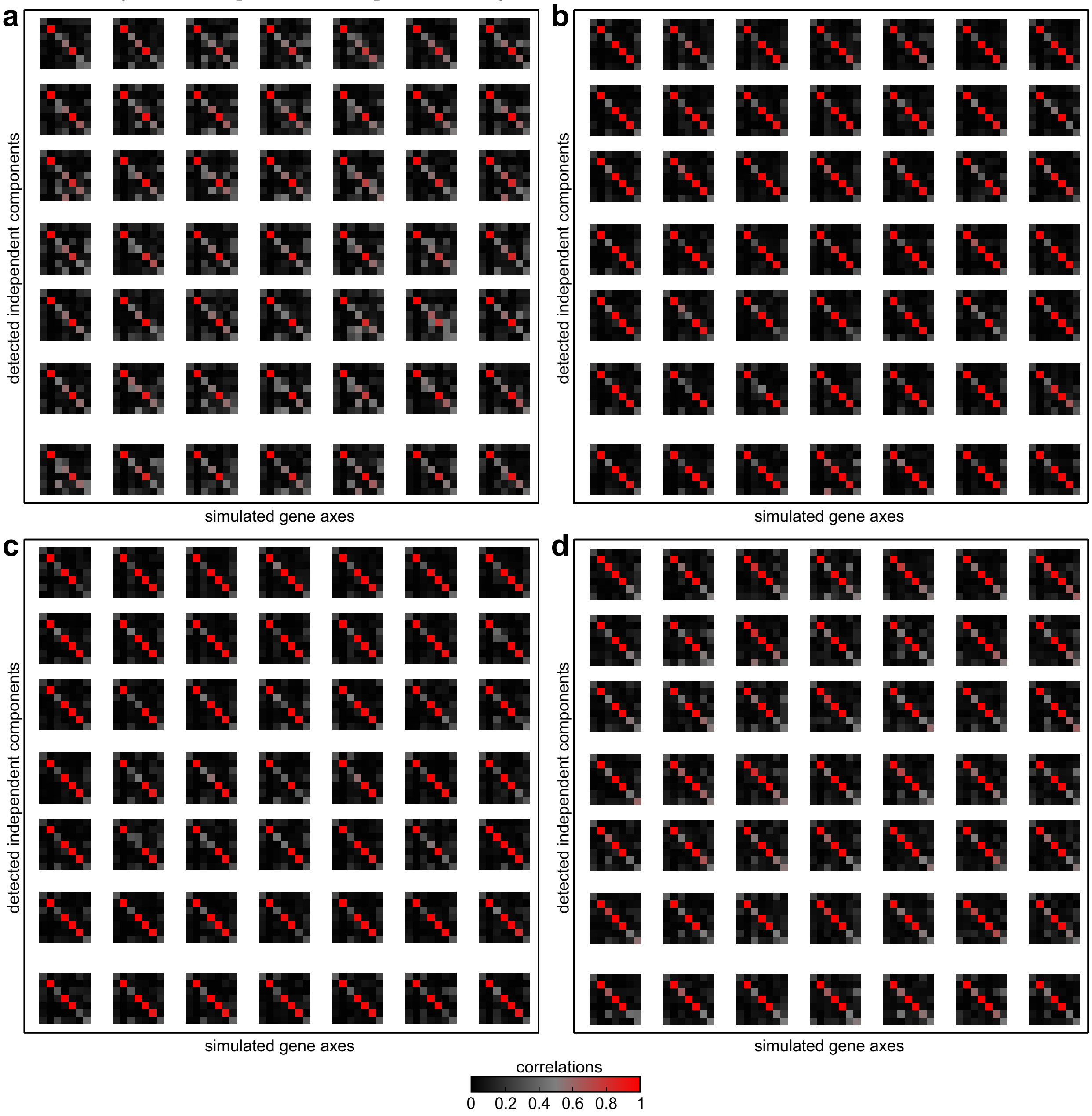

ICA results for 49 simulations of the 7-signature versatility scenario (cf. Fig. 3a). **(a)** Results for the default pow3 score function of the current fastica implementation v2.5[35]. Detected ICs were compared with simulated gene axes (Supplementary Note 8). Depicted are the top seven ICs with the highest absolute correlations to any simulated gene axes. Signatures #2 and #5 were detected with high sensitivity (a red pixel exists in respective columns) and high specificity (no secondary red or gray pixel exists in the same columns). All other signatures were not detected or only with weak correlation to their true simulated gene axes. **(b)** ICA results for the tanh score function. Signatures #2, #4, #5 and #6 were detected with high sensitivity and specificity, but other signatures were not detected. **(c)** ICA results for the gauss score function. Results are similar to tanh, but signature #4 was detected less often. In one run, signature #3 was detected as well. **(d)** ICA results for the skew score function. Results are similar to tanh and gauss, but signature #6 was no longer detected with high correlation. Signature #3 was detected more often.

Fig. 6: Versatility test, principal component analysis

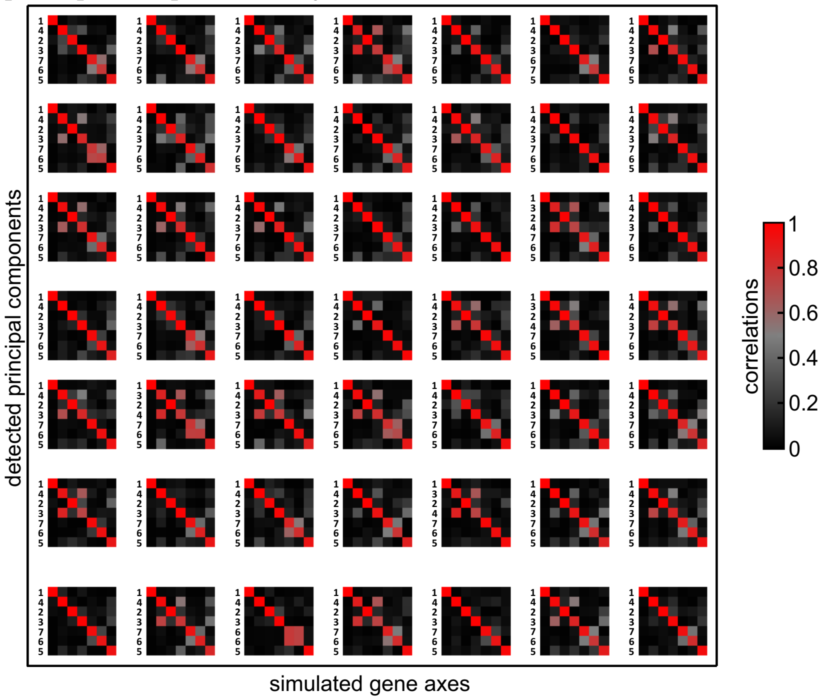

PCA results for 49 simulations of the 7-signature versatility scenario (cf. Fig. 3a) using the pca function in MATLAB®. Detected PCs were compared with simulated gene axes (Supplementary Note 8). Depicted are the top seven PCs by variance (numbers indicate variance ranks). All seven signatures were detected reliably with high sensitivity (a red pixel exists in each column). The specificity for signature #1 was excellent (only black pixels exist in column one beside the single red pixel). Specificity for signatures #2,#4,#5 and #6 was suboptimal in several runs (their gene axes were correlated to and explained by multiple PCs, i.e. their signal was not dissected completely). Overall, PCA results were significantly better than results by HC, NNMF, FABIA, FABIAS or ICA (cf. Supplementary Figs. 2-5).

Fig. 7: 3D test signal, hierarchical clustering

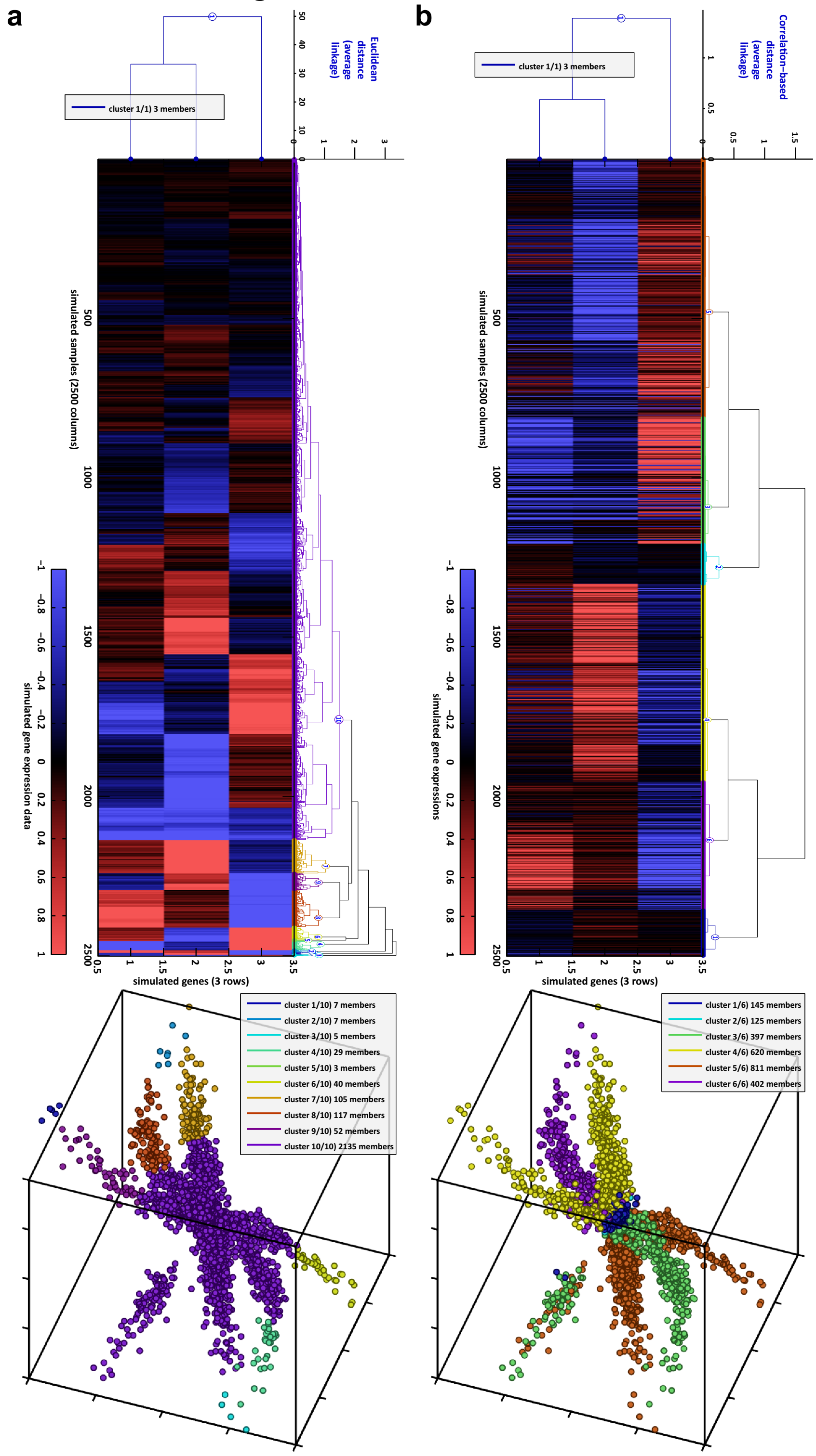

(a) HC results for the 3D test signal (from Fig. 1), using the default configuration (Euclidean distance and average linkage). Sample points in the 3D gene space are colored by detected sample clusters (bottom). Most samples were combined by HC in one large cluster around zero. At the tips of simulated interactions, smaller sample clusters were formed. All four simulated interactions were split over several clusters. **(b)** HC results using the correlation based distance (as implemented by the pdist function in MATLAB®). Simulated interactions are split into fewer clusters, but signal parts of the same interaction on different sides of the center are still split into distinct clusters. Additionally, different interactions on the same side of the center are mixed by merging them into just one cluster.

Fig. 8: 3D test signal, biclustering with FABIAS

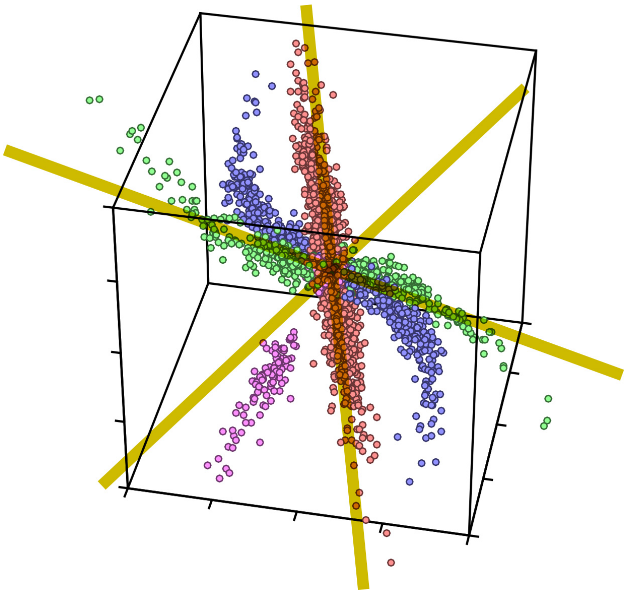

We applied FABIAS to our 3D test signal, using its best configuration from the versatility test (i.e. $\alpha = 0.6$, cyc = 200, spz = 1; cf. Supplementary Fig. 4c). Lines depict column vectors of the resulting loadings matrix. Comparing with PCA results (Fig. 1i), FABIAS performs better in that it aligns one vector with the gene axis of the red effect. However, like PCA, FABIAS does not support more biclusters than the data has dimensions and, therefore, principally cannot find all four simulated gene axes.

Fig. 9: Versatility test with 13 signatures for performance comparison

For a detailed comparison of PCA with SDCM, we simulated a versatility scenario with 13 superposed signatures (see main text) 49 times. Detected PCs respectively gene axes were compared with simulated gene axes (Supplementary Note 8). Matrices depict correlations as before (each column corresponds to a simulated signature axis and each row to a detected signature axis respectively PC). Table 3 summarizes these results numerically. **(a)** PCA results (obtained with the pca function in MATLAB®) are depicted for the top 13 PCs by variance (numbers indicate variance ranks). Sensitivity and specificity for the large signature #1 was still excellent, like for the 7-signature versatility test in Supplementary Fig. 6 (there are only black pixels in column one beside the first red pixel). While most other signatures were detected in most runs (i.e. a red pixel exists in most columns), the specificity was often only moderate (other gray or red pixels exist in the same column). **(b)** SDCM results for identical simulations of the 13-signature versatility test. Depicted are all detected gene axes returned by SDCM, including false positives, if any (numbers indicate detection iterations). Compared to the 7-signature versatility test (Supplementary Fig. 1), a few more false positives were returned (rows without any red pixel) and detection specificity was not optimal in more cases (more than one red pixel in the same column). Overall however, high sensitivity and specificity were retained.

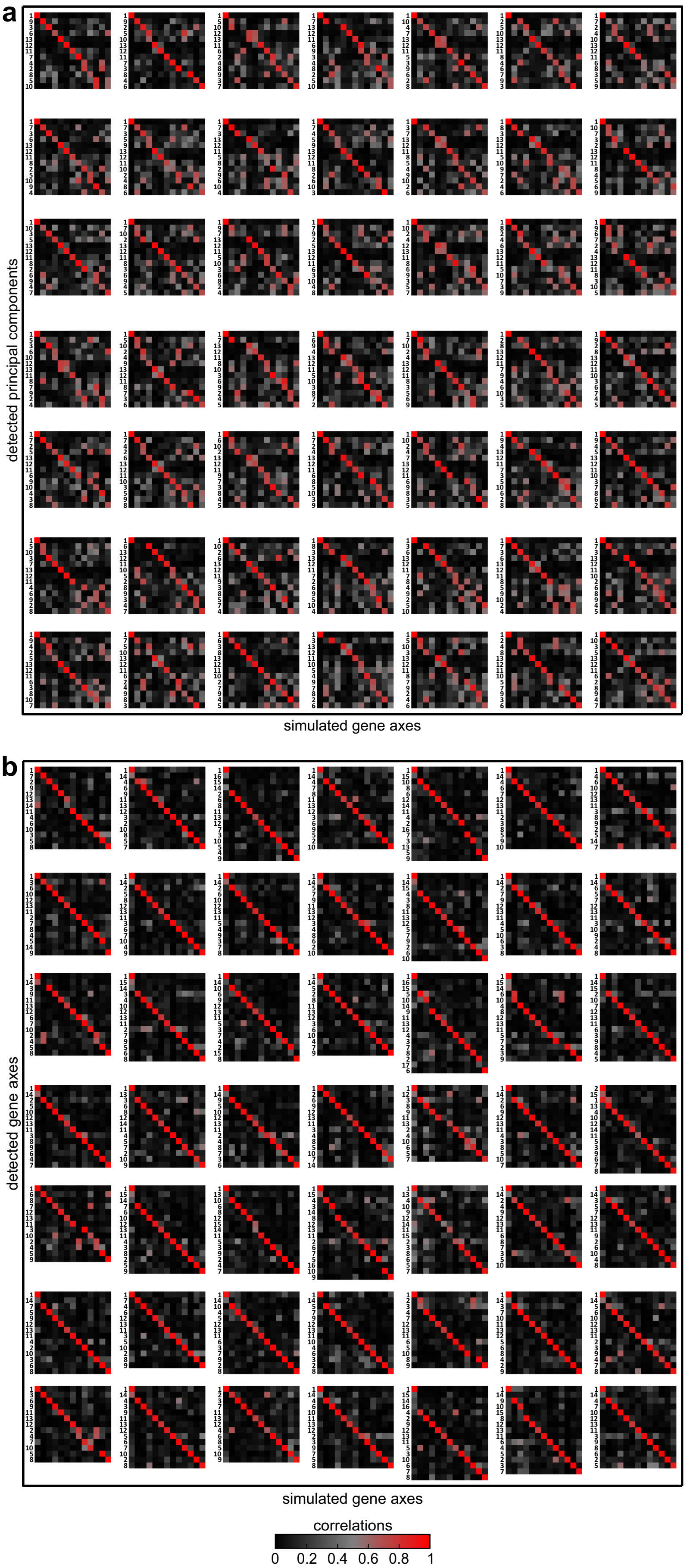

Fig. 10: Weak signal sensitivity test

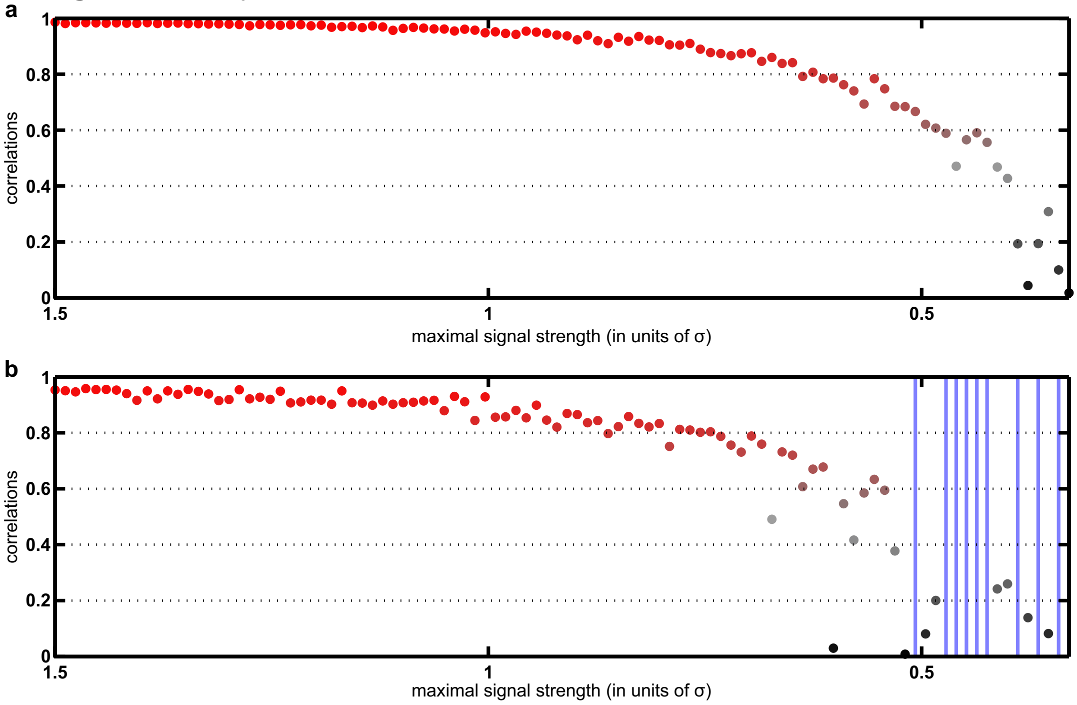

To test detection sensitivity limits, we simulated signals containing a single instance of signature #6 from the versatility scenario (see Fig. 3a in main text) for decreasing signal strength relative to the noise level σ. Detected gene axes (respectively the top PCs by variance) were compared with the simulated gene axis as before (Supplementary Note 8). **(a)** PCA yielded higher correlations for these test signals than SDCM. It retained high correlation ($r > 0.8$) for weaker signals (down to about 0.63σ). As PCA does not determine the number of signatures in the signal, it returned PCs even for arbitrarily weak signal strengths $\ll 0.5\sigma$, but here PCs were essentially false positives ($r \leq 0.2$). **(b)** SDCM detected this signature with high correlation ($r > 0.8$) down to $\sim 0.75\sigma$. Below this value, first accuracy decreased (lower correlation with the simulated gene axis). Below $\sim 0.5\sigma$, SDCM often terminated without any detected signature (blue lines). SDCM never detected two or more signatures in these 1-signature signals.

Fig. 11: Superposition test for signature #3

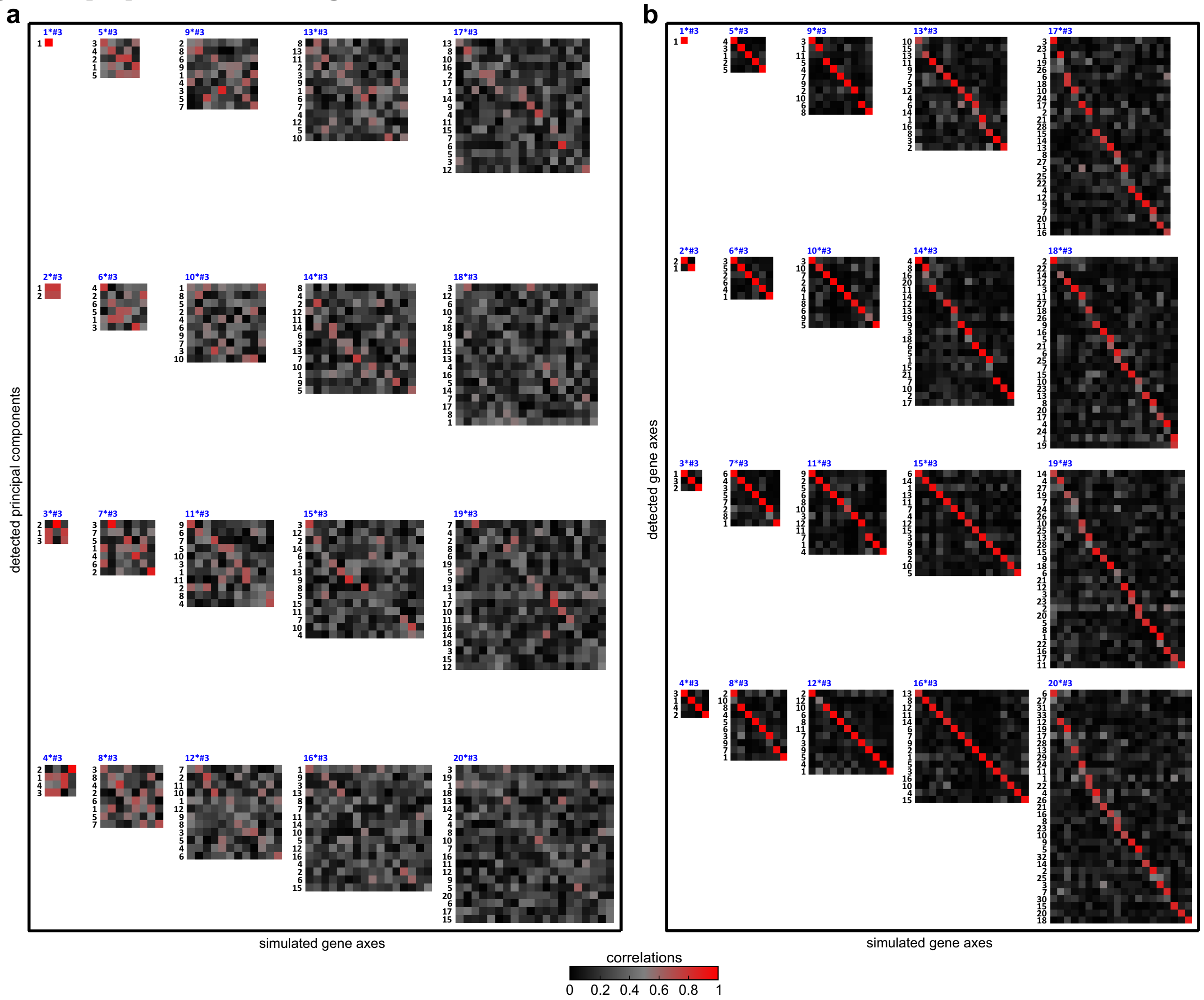

To test detection and dissection limits with respect to superposition depth, we superposed an increasing number (blue) of instances of signature #3 from the versatility scenario (see Fig. 3a). To keep the overall signature average at zero despite the asymmetric form of signature #3, instances were simulated with alternating signs. **(a)** Detected PCs were compared with simulated gene axes (Supplementary Note 8). Depicted are the top PCs by variance according to the true number of simulated signatures (no false positives are shown; numbers indicate variance ranks). Sensitivity and specificity of PCA broke down in this superposition test series. In particular, the signal of simulated signatures was split over several PCs (multiple gray pixels in the same column of depicted correlation matrices). **(b)** Gene axes detected by SDCM were compared with simulated gene axes. Depicted are all detected gene axes returned by SDCM, including false positives, if any (numbers indicate detection ranks). SDCM retained high sensitivity and specificity overall. With increasing number of superposed signatures, also the number of false positives (no red pixel in a row) and double detections due to incomplete first dissections (a single red pixel in the same column as for another row) became more frequent.

Fig. 12: Superposition test for signature #4

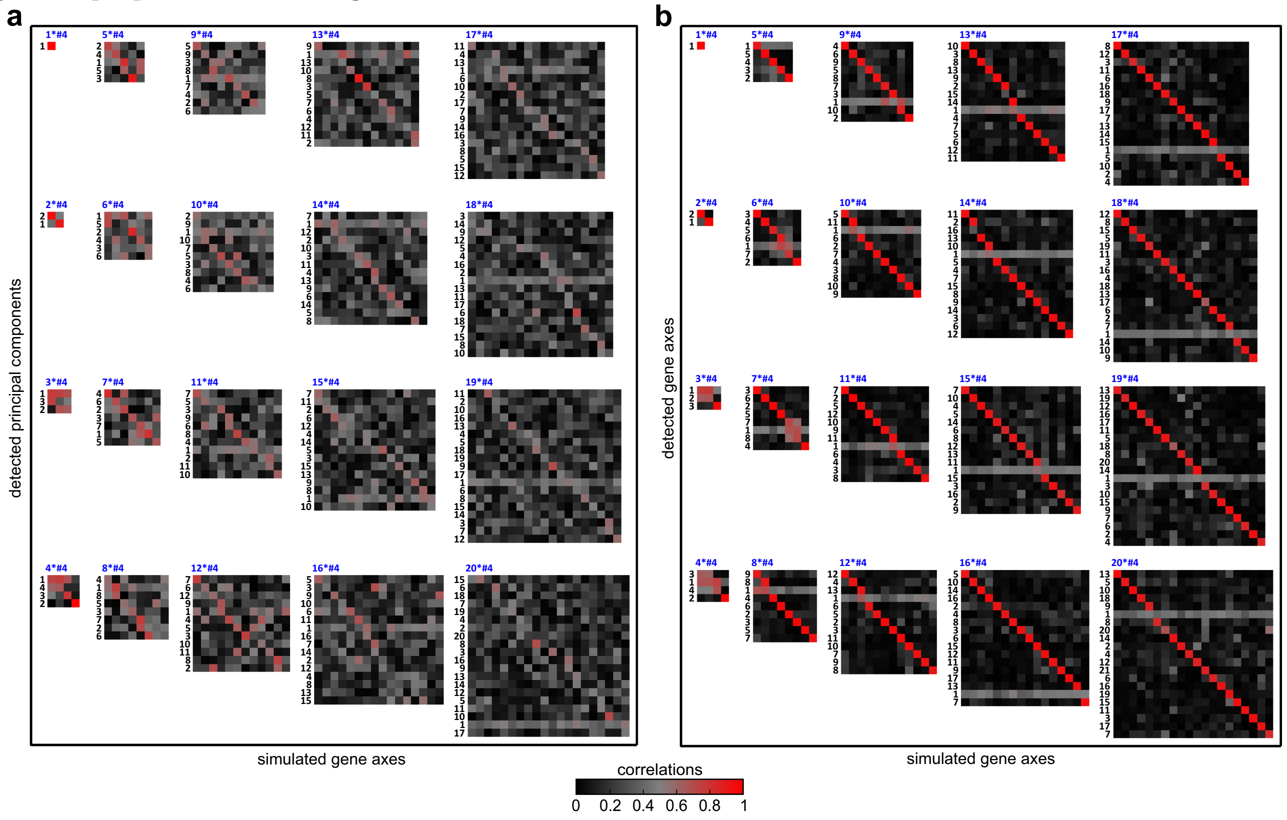

To test detection and dissection limits with respect to superposition depth, we superposed an increasing number (blue) of instances of the one-sided signature #4 from the versatility scenario (see Fig. 3a). As this signature is asymmetric and we simulated it without altering signs, an overall negative signal average accumulates with the number of simulated instances. **(a)** Detected PCs were compared with simulated gene axes (Supplementary Note 8). Depicted are the top PCs by variance according to the true number of simulated signatures (no false positives are shown; numbers indicate variance ranks). As for instances of signature #3 (Supplementary Fig. 11), sensitivity and specificity of PCA broke down. **(b)** Gene axes detected by SDCM were compared with simulated gene axes. Depicted are all detected gene axes returned by SDCM, including false positives, if any (numbers indicate detection ranks). SDCM retained high sensitivity and specificity overall. The accumulating negative offset was not detected separately for five instances of signature #4 or less. The offset causes a loss of sensitivity and specificity, especially for three and four instances of signature #4. From eight instances onwards, the offset was always detected as a separate signature in the first SDCM iteration (light gray rows). Additionally, it was dissected in a way that enabled highly sensitive and specific detections of all superposed instances of signature #4 in subsequent SDCM iterations. Compared to the superposition test for signature #3 (Supplementary Fig. 11), no double-detections occurred for this one-sided signature.

Fig. 13: Superposition test for signature #6

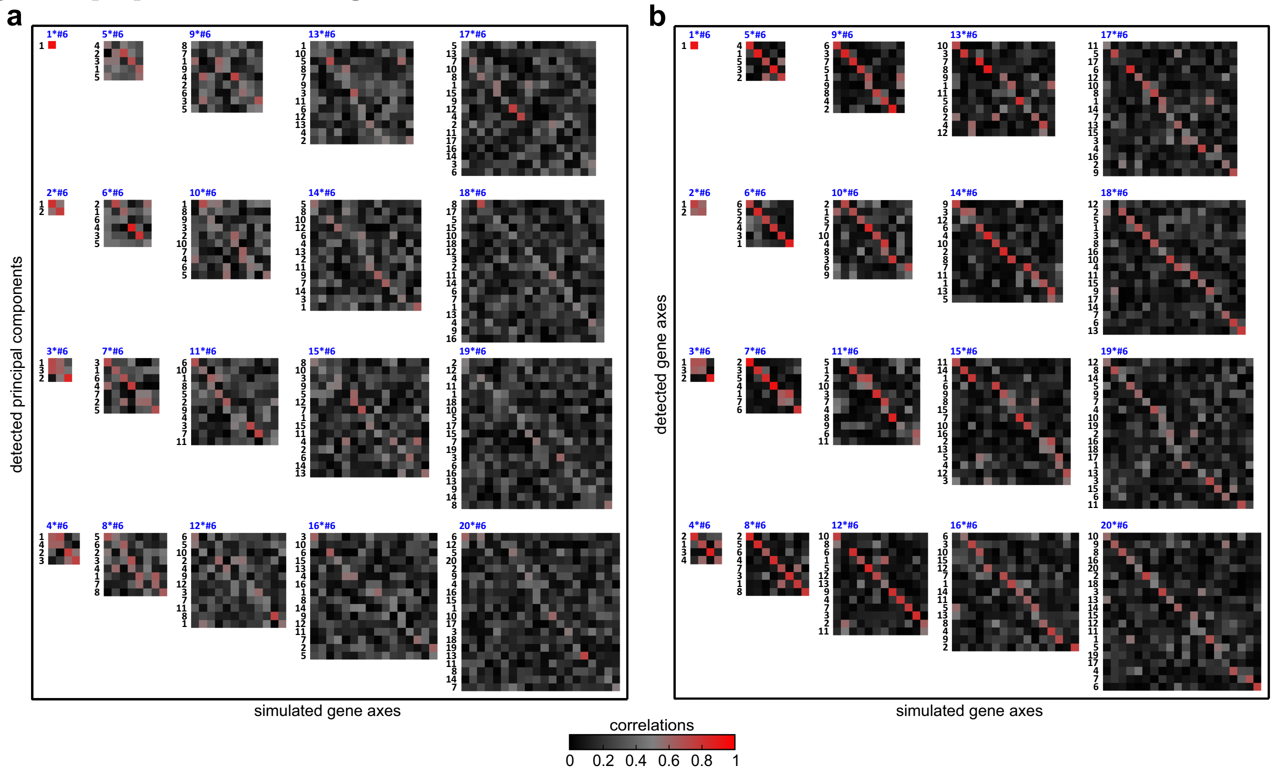

As detection and dissection limits with respect to superposition depth were not reached for SDCM with signatures #3 and #4 (Supplementary Figs. 11 and 12), we repeated the superposition test with signature #6 whose maximal absolute signal just equals the simulated noise level σ (see Fig. 3a). **(a)** Detected PCs were again compared with simulated gene axes (Supplementary Note 8). Depicted are the top PCs by variance according to the true number of simulated signatures (no false positives are shown; numbers indicate variance ranks). Again, sensitivity and specificity of PCA broke down. **(b)** Gene axes detected by SDCM were compared with simulated gene axes. Again, all detected gene axes returned by SDCM are depicted, including false positives, if any (numbers indicate detection ranks). While results were still significantly better than for PCA (panel a), sensitivity and specificity were moderate at best and broke down for higher superposition depths, as expected.

Fig. 14: Empirical algorithm complexity

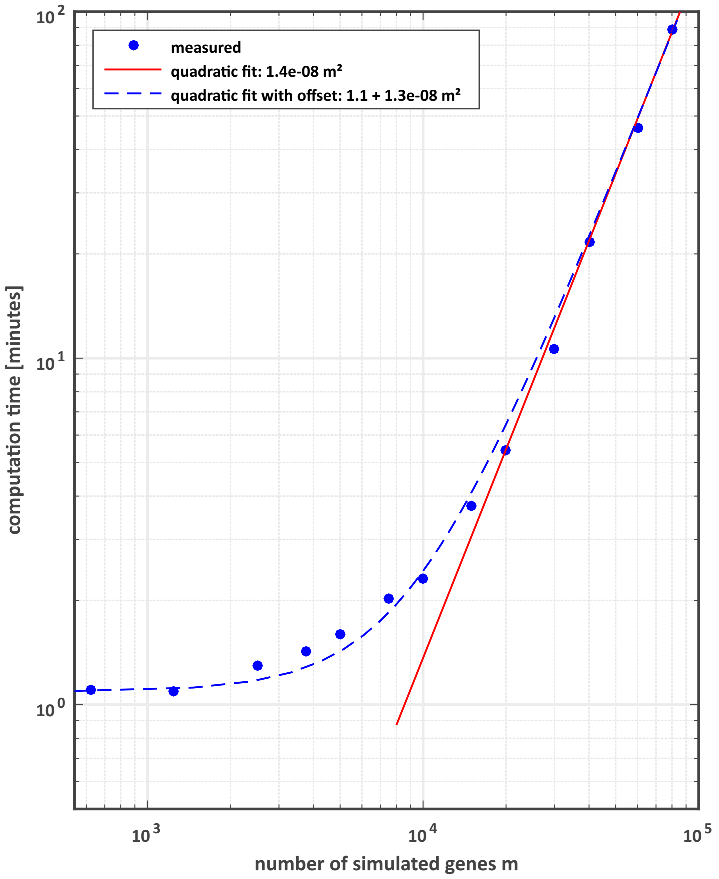

To measure SDCM performance, we simulated signals with $n = 100$ samples for gene spaces with increasing dimension up to $m = 80000$. Each signal contained a single instance of signature #3 from the versatility test (Fig. 3a), for randomly selected genes and samples. As our default configuration allows for some false positives in order to increase detection sensitivity (see, e.g., Supplementary Fig. 9b), we adjusted the signal significance parameter α_s for this performance test so that only the single simulated signature qualified for all m and no FPs, leading to comparable computation times. All computations were performed in a virtual machine on an Intel Xeon E5 host at 3.2GHz using four parallel MATLAB® workers. Computation time was measured from the start of the first search strategy until returning results.

The asymptotic runtime increase is, in good approximation, quadratic in m (red line), as was expected by analytical complexity estimation (see Methods/Algorithmic complexity). The offset at $m \to 0$ (blue fitting model) accounts for computation time that is independent of m, e.g., for bimonotonic regression in the subspace defined by the signature.

Fig. 15: Missing values test

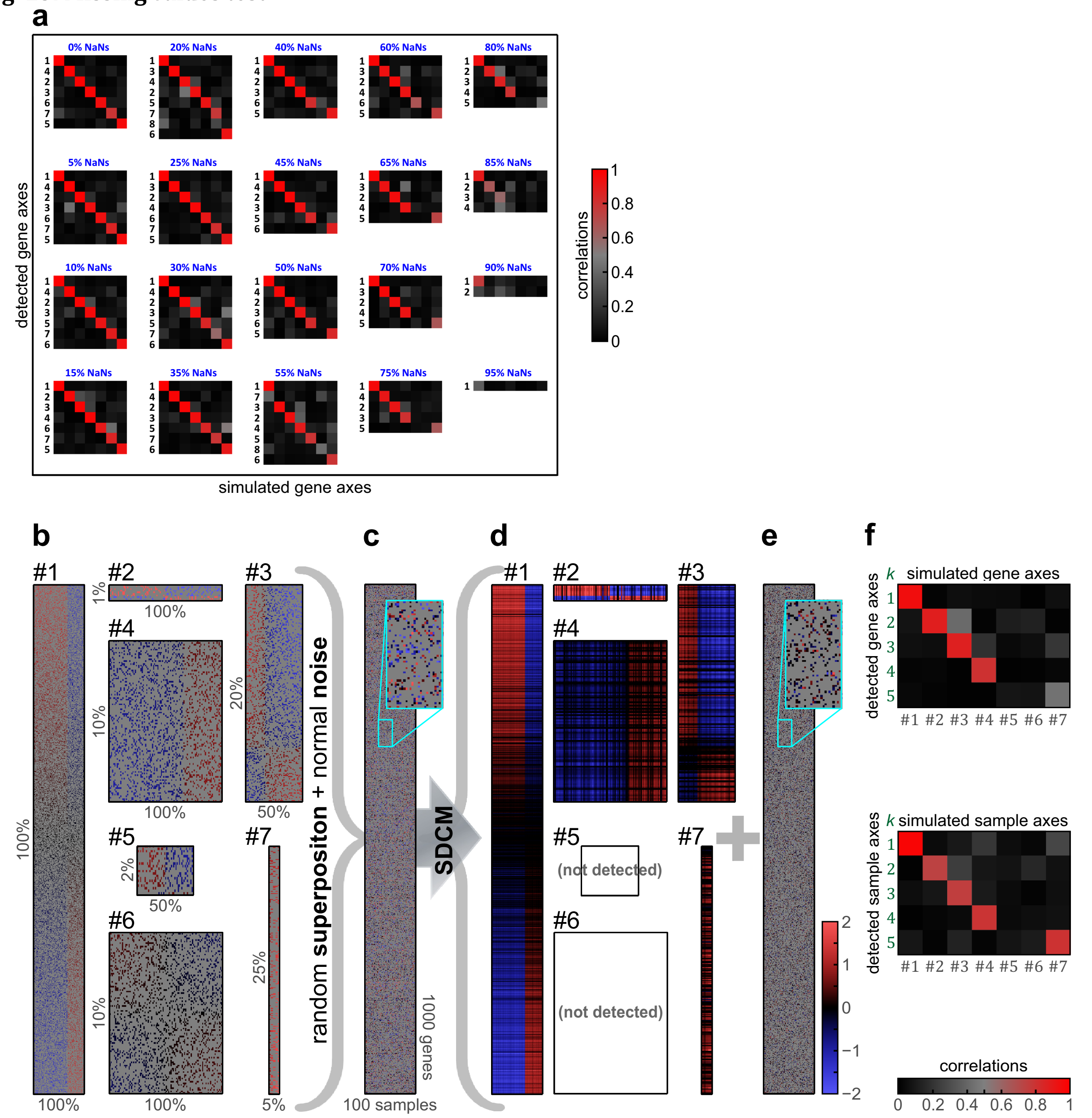

SDCM can handle missing values. To test detection limits in this context, we simulated a series of the 7-signature versatility test (Fig. 3a) for increasing ratios of randomly selected missing values. **(a)** SDCM detection results are presented for 0% to 95% missing values. Detected gene axes were compared with simulated ones as before (Supplementary Note 8). Correlation matrices show a sensitive and specific detection of all signatures except for the weak signature #6 up to 55% missing values. Signatures #1-#4 were detected with high correlation up to 80% missing values. With 90% missing values, only the large signature #1 was still detected. For 95% missing values, SDCM terminated after one signature that is only very weakly correlated to signature #1. **(b-f)** Detailed SDCM results for the 7-signature versatility test with 80% missing values are presented analogously to Fig. 3 in the main text. Signatures #1-#4 were still detected with high correlations (panel f). Samples affected by signature #7 were still correctly identified (high sample axis correlation), but gene axis correlation for #7 was low, indicating only a rough detection of participating genes. Signatures #5 and #6 were not detected. Signature signals (panel d) reconstructed many missing values from detected correlations, but reconstruction failed for genes and samples for which almost all values were missing (black rows and columns).

Fig. 16: Detected gender-associated signature

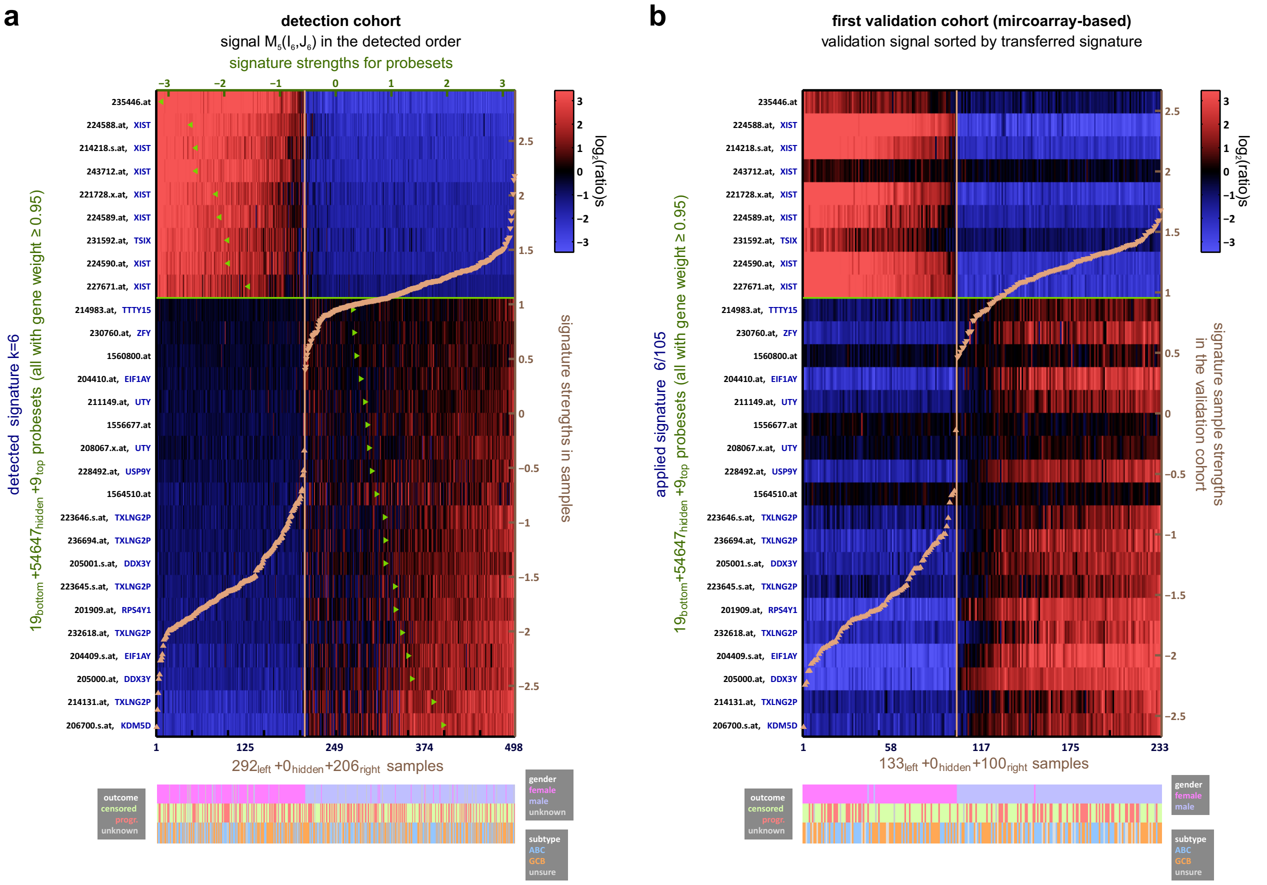

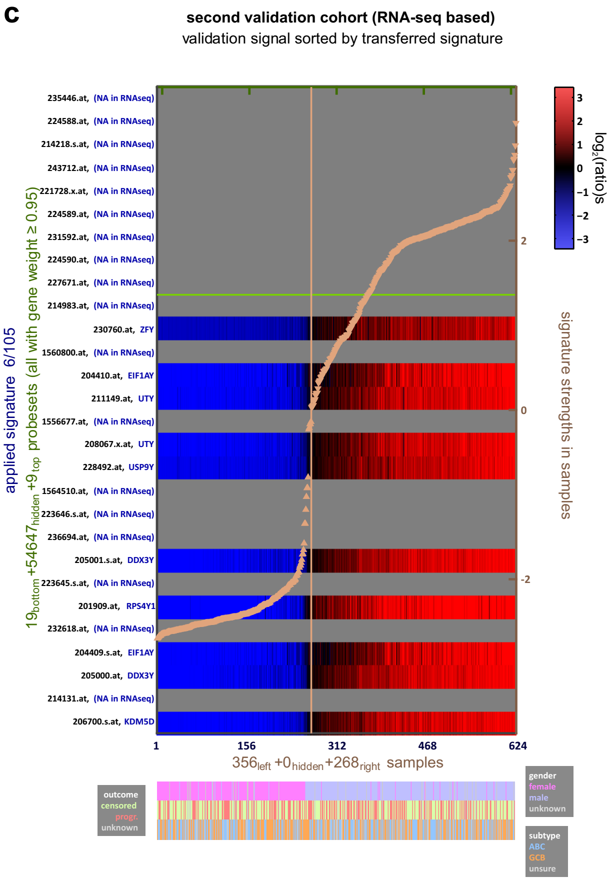

(a) In iteration $k = 6$, SDCM discovered this signature for the detection cohort (comprised of tumor samples from 498 patients). Gene expression data are shown as $\log_2(\text{ratios})$ relative to cohort means. The signal is ordered by signature strengths (green for rows, representing genes or measured transcript clusters, and light brown for sample columns). The detected signature signal was two-sided, i.e. this signature has both highly correlated transcript clusters (lower half of rows with positive strengths) as well as highly anti-correlated transcript clusters (upper half of rows with negative strengths). Sample strengths (light brown) had a bimodal distribution with a gap around zero, resulting in two clearly defined sample classes. Colored lines (bottom) indicate sample gender, disease progression and DLBCL subtype, respectively. We noticed a significant association with gender ($p = 2.8 \cdot 10^{-83}$, χ^2-test). There were 9 male, 185 female and 12 unknowns for negative sample

strengths, as well as 262 male, 14 female and 16 unknowns for positive sample strengths. All sample strengths, including IDs for all 23/498=4.6% of samples for which the gender association did not hold, are provided in Supplementary Data 2.

(b) The detected signature was transferred (Supplementary Note 11) and applied to the validation cohort (comprised of tumor samples from 233 independent patients, same microarray platform). Here, transcript clusters showed likewise correlations, validating this signature on GE level. Signal strengths of transcript clusters differed; a possible origin of these differences may be the use of paraffin–embedded tumor samples for the detection cohort versus fresh-frozen material for the validation cohort. As in (a), sample strengths gave rise to two clearly defined sample classes that were significantly associated with gender. There were 2 males and 98 females for negative sample strengths, as well as 132 males and 1 female for positive sample strengths ($p = 5.7 \cdot 10^{-50}$, χ^2-test). All sample strengths, including sample IDs for all 3/233=1.3% mismatches, are provided in Supplementary Data 2.

(c) Despite incomplete mapping due to microarray probe sets for non-coding RNAs such as XIST (RNA-sequencing data was mapped to the coding transcriptome only), gene-gene correlations in this binary signature were strong enough to again clearly separate samples by gender in this second add-on validation cohort by ($p = 6.2 \cdot 10^{-98}$, χ^2-test). This confirms this signature as a proof-of-concept from a method validation perspective and serves as an independent self-control for the underlying RNA-sequencing analysis and for imported sample annotations of this cohort.

Fig. 17: Rediscovered DLBCL signature Stromal-1

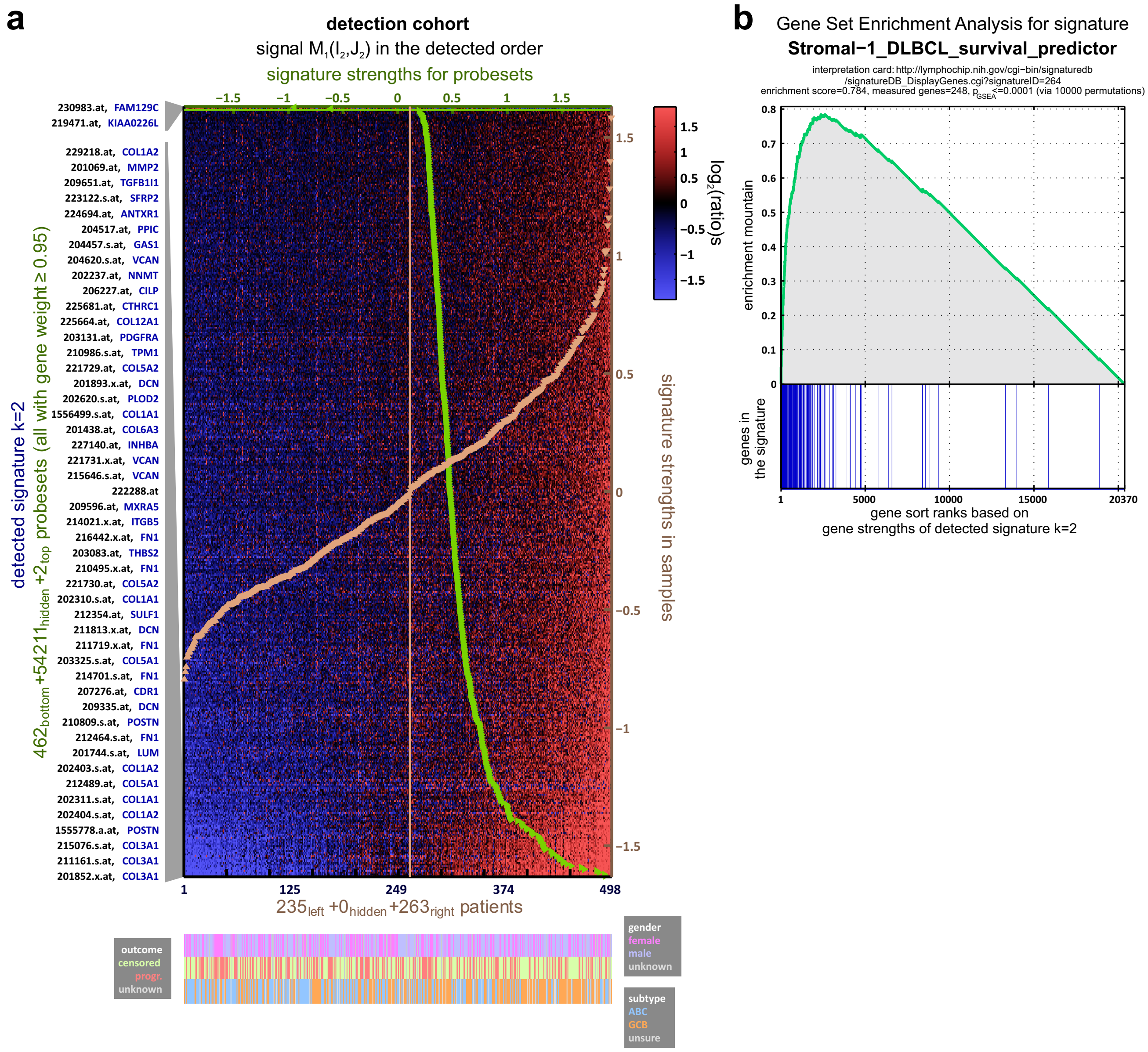

(a) A one-sided signature was detected in iteration $k = 2$. It is presented as described for the gender-associated signature (Supplementary Fig. 16). **(b)** The previously defined gene signature Stromal-1_DLBCL_survival_-predictor[2] was significantly enriched at top correlated genes of this detected signature (enrichment score 0.784 with $p \leq 10^{-4}$ by permutation test).

Fig. 18: Rediscovered DLBCL signature Stromal-2

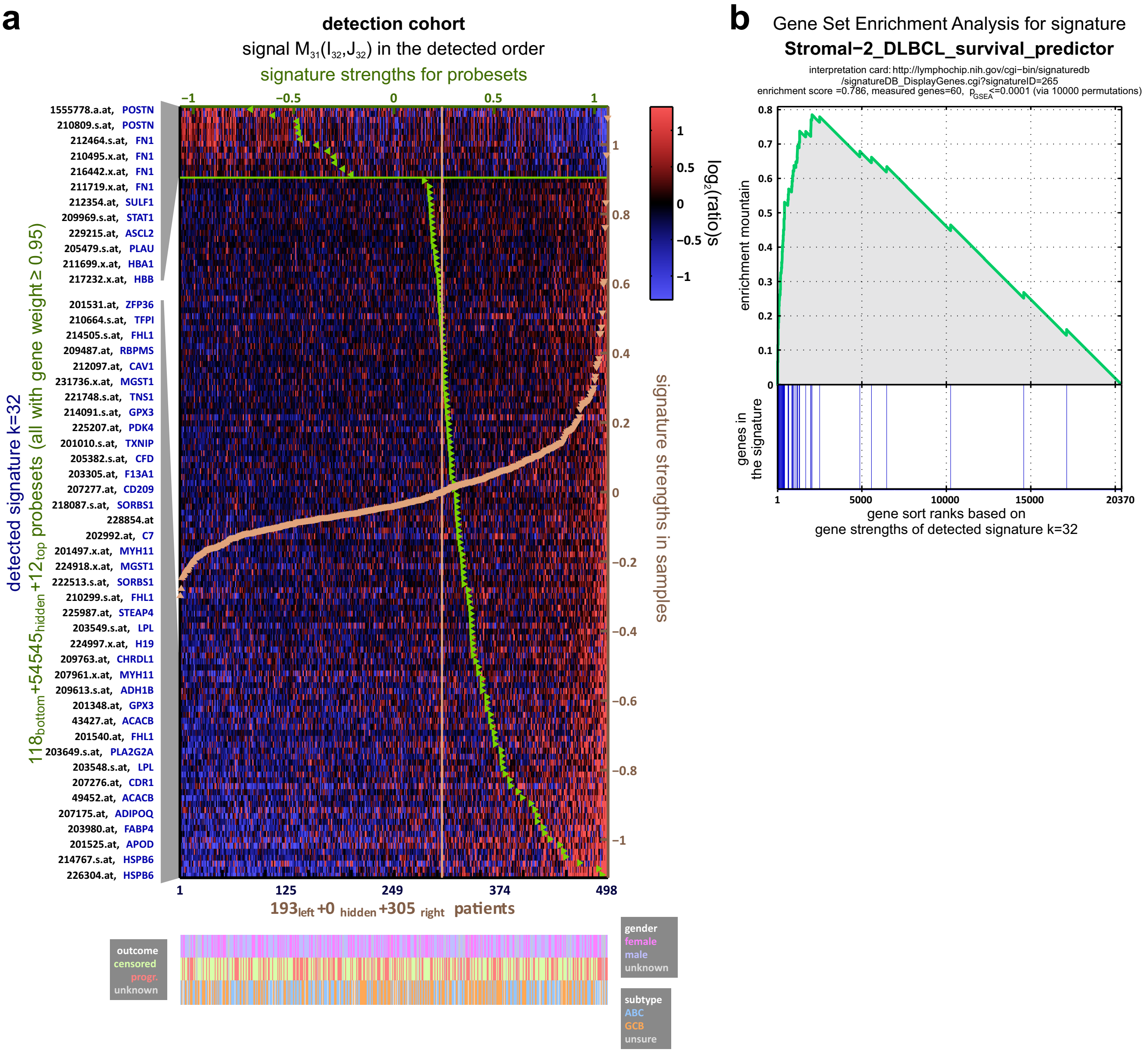

(a) A predominantly one-sided signature was detected in iteration $k = 32$; it is presented as described for the gender-associated signature (Supplementary Fig. 16). **(b)** The previously defined gene signature Stromal-2_-DLBCL_survival_predictor[2] was significantly enriched at its top correlated genes (enrichment score 0.786 with $p \leq 10^{-4}$ by permutation test).

Fig. 19: Rediscovered cell-of-origin related DLBCL signatures

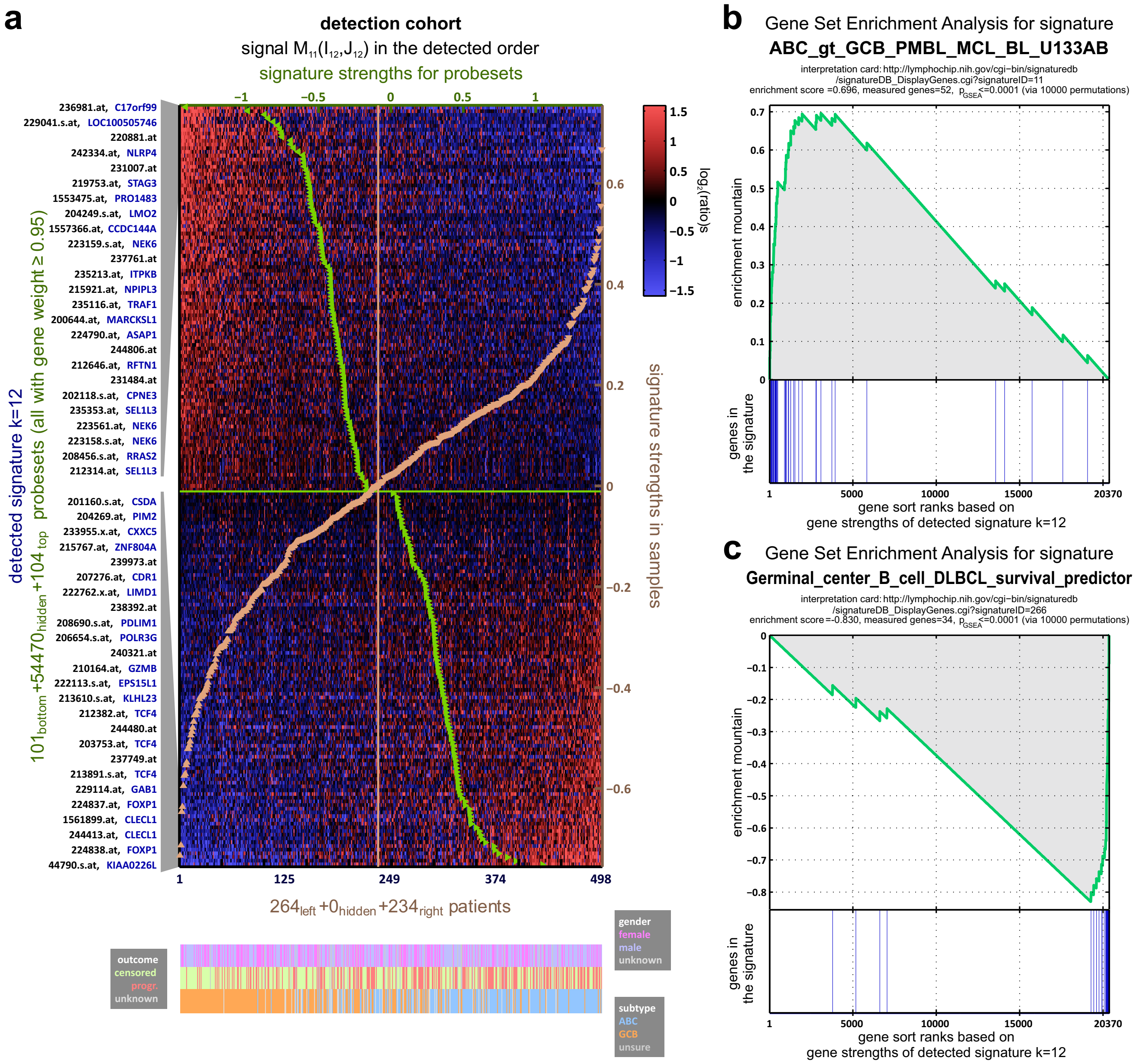

(a) A two-sided signature was detected in iteration $k = 12$; it is presented as described for the gender-associated signature (Supplementary Fig. 16). Indicator bars at the bottom show a clear survival trend and subtype arrangement, but no association with gender. **(b)** The previously defined gene signature ABC_gt_-GCB_PMBL_MCL_BL_U133AB[40] was significantly enriched at top correlated genes of the detected signature (enrichment score 0.696 with $p \leq 10^{-4}$ by permutation test). **(c)** At top anti-correlated genes, the gene signature Germinal_center_B_cell_DLBCL_survival_predictor[2] was significantly enriched (enrichment score -0.830 with $p \leq 10^{-4}$ by permutation test). Together, these associations confirm the unsupervised rediscovery of cell-of-origin related DLBCL signatures.

Fig. 20: Overview and characteristics of all 105 detected SDCM signatures

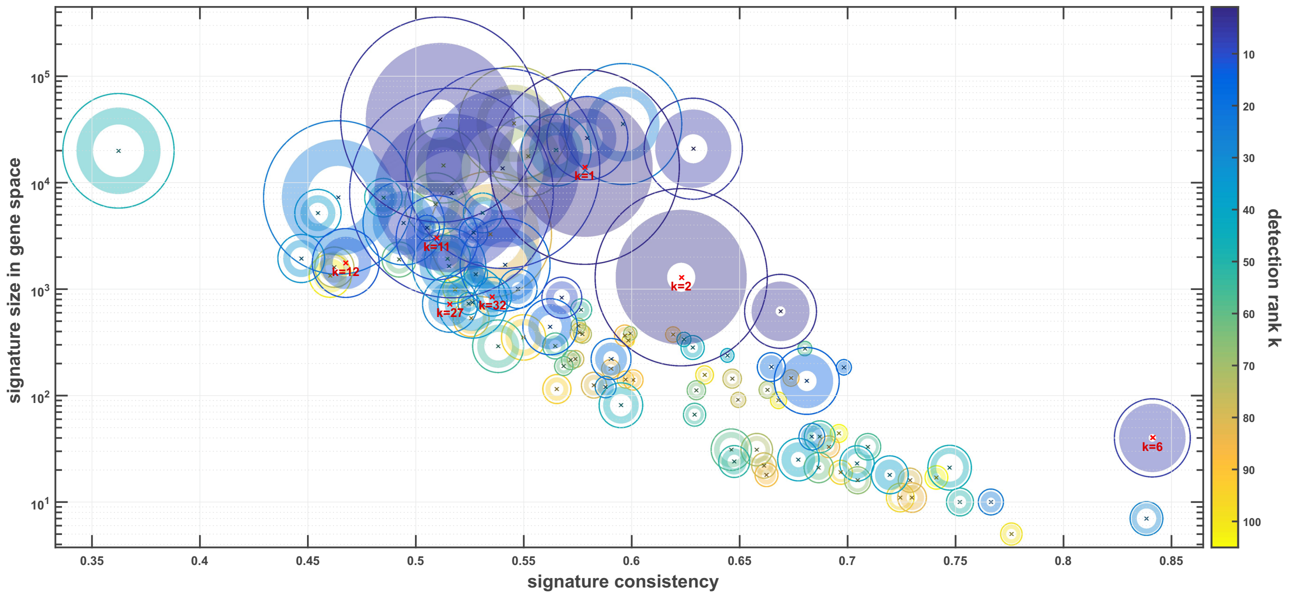

Signature size vs. signature consistency for all 105 detected signatures. Signature size is the number of unique probesets having full weight $\langle \mathbf{e}_i^{\mathrm{g}} | \mathbf{w}^{\mathrm{g}} \rangle = 1$. Signature consistency is quantified by the mean absolute correlation of all probesets and samples having full weight, i.e. $\left(\mathrm{mean}\left(|\langle \mathbf{e}_i^{\mathrm{g}} | \mathbf{r}^{\mathrm{g}} \rangle|\right) + \mathrm{mean}\left(|\langle \mathbf{e}_j^{\mathrm{s}} | \mathbf{r}^{\mathrm{s}} \rangle|\right)\right)/2$.

Independent of size and consistency, filled areas depict the amount of signal as sum of $|\log_2(\text{ratio})\text{s}|$ that is explained by the corresponding dissected signature signal. Signatures detected first explain most of the signal (blue areas). Outer circles indicate the remaining signal in the signature's focus as sum of remaining $|\log_2(\text{ratio})\text{s}|$ after signature dissection, including noise. Empty inner circles of rings depict the signal that has already been explained by previous dissections in the current signature focus. The thinner the filled ring area the stronger the overlap with the previously detected signatures.

Most of the signatures with higher ranks (green-yellow) highly overlap (thin rings) or explain only relatively small parts of the signal (small filled areas and a low number of unique probesets). Smaller signatures usually have a higher inner consistency. However, small signatures need to be critically validated, as in the extreme case, they might only consist of different probesets for a signal gene.

Normally, signatures with fewer unique probesets also explain less of the signal. An exception is the gender-associated signature $k = 6$ due to strong $\log_2(\text{ratio})$s of its member probesets between males and females. For the same reason, its corresponding signal-to-noise ratio is the best of all signatures, resulting in the highest consistency of all.

Both discovered survival signatures $k = 11$ and $k = 27$ have higher consistency than the rediscovered COO-related signature $k = 12$. This again underlines our hypothesis that survival signatures $k = 11$ and $k = 27$ provide a more precise representation of underlying biological differences.

Fig. 21: Method comparison for real data, SDCM versus PCA

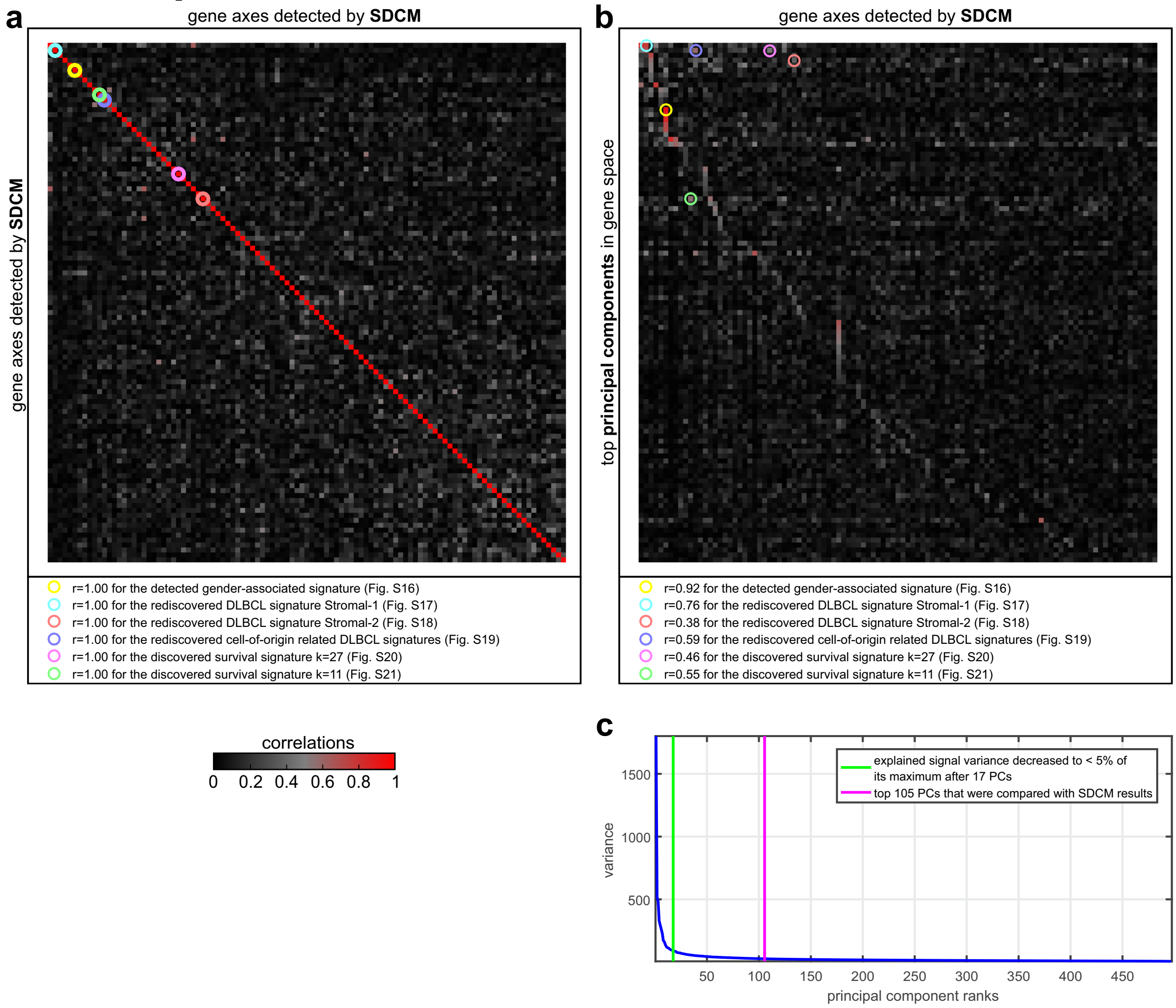

SDCM and PCA results for the detection cohort (GSE31312, 54675 transcript clusters, tumor samples form 498 R-CHOP treated patients). **(a)** Signature gene axes discovered by SDCM were compared with each other (Supplementary Note 8) to display partial correlations between signatures. **(b)** Comparison with PCA. Detected PCs (rows) were compared (Supplementary Note 8) with SDCM gene axes (columns). Depicted are the 105 PCs having top variance. PCA redetected the gender-associated signature ($r = 0.92$), but several PCs have gender-associated genes among their top genes (incomplete dissection, i.e. the signature is not explained by just one PC). One PC has $r = 0.76$ to top genes of the Stromal-1 signature, but the same PC also has high correlation to SDCM signature $k = 1$ (see first column, i.e. this PC represents a mixture of distinct signatures). Another PC mixes the Stromal-2 signature ($r = 0.38$) and survival signature $k = 27$ ($r = 0.46$); no PC has better correlation to these biological signatures. Overall, PCA results show little overlap with SDCM results for real data. While PCA performed well for the versatility test with signatures of distinct variances (cf. Supplementary Fig. 6), its real data results resemble those of the superposition tests (Supplementary Figs. 11-13). I.e., PCs point along directions of maximal signature overlap, failing to dissect partially correlated but distinct signatures. **(c)** Variances explained by each PC. PCA explains most of the signal's variance with only few PCs (after 17 PCs, the explained variance per PC is already less than 5% of the maximum). However, describing the signal by only few dimensions of maximal variance seems to have the disadvantage that PCs often represent mixtures of multiple biologically distinct signatures.

Fig. 22: Method comparison for real data, SDCM versus ICA

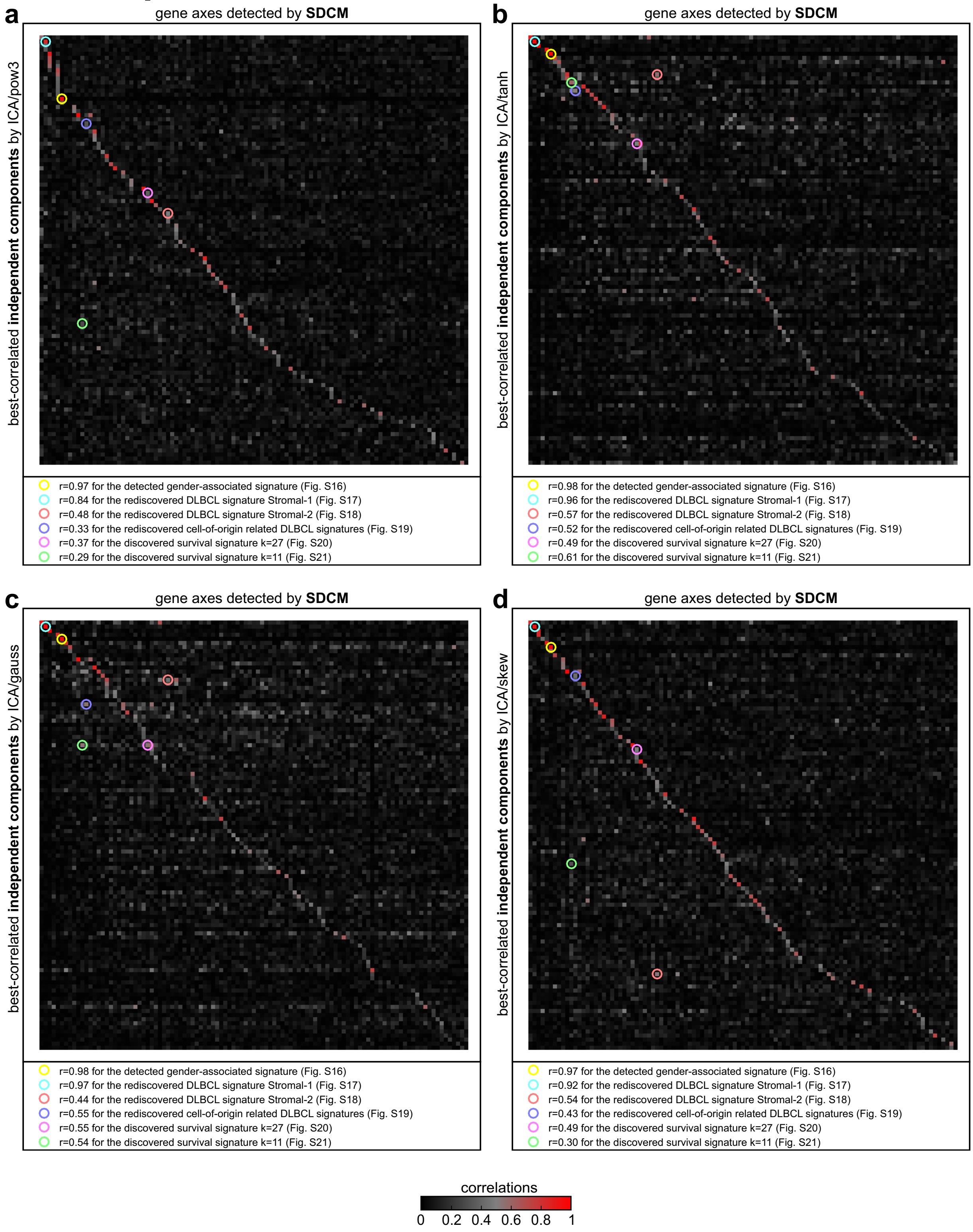

ICA results for the detection cohort (GSE31312, 54675 transcript clusters, tumor samples from 498 R-CHOP treated patients). **(a)** Results for the default pow3 contrast function (cf. Eqn. 16 in [35]) of the current fastica implementation v2.5. Detected ICs were compared with SDCM gene axes (Supplementary Note 8). Depicted are the 105 ICs with the highest absolute correlations to any SDCM gene axis. The gender-associated signature ($r = 0.97$) and the Stromal-1 signature ($r = 0.84$) were rediscovered with high correlations, while the other four biological signatures were not represented well by any IC ($r < 0.5$). **(b)** ICA results for the tanh contrast function[35]. Besides excellent rediscovery of both the gender-associated signature ($r = 0.98$) and the Stromal-1 signature ($r = 0.96$), ICA/tanh

also performed better for the remaining four biological signatures than ICA/pow3. Still, correlations of top genes were only moderate. **(c)** ICA results for the gauss contrast function [35]. Results were similar to tanh. **(d)** ICA results for the skew contrast function [35]. Results were similar to tanh and gauss. Overall, ICA/skew rediscovered more SDCM signatures than any other comparison method (count of columns having a red pixel).

Fig. 23: Method comparison for real data, SDCM versus FABIA and FABIAS

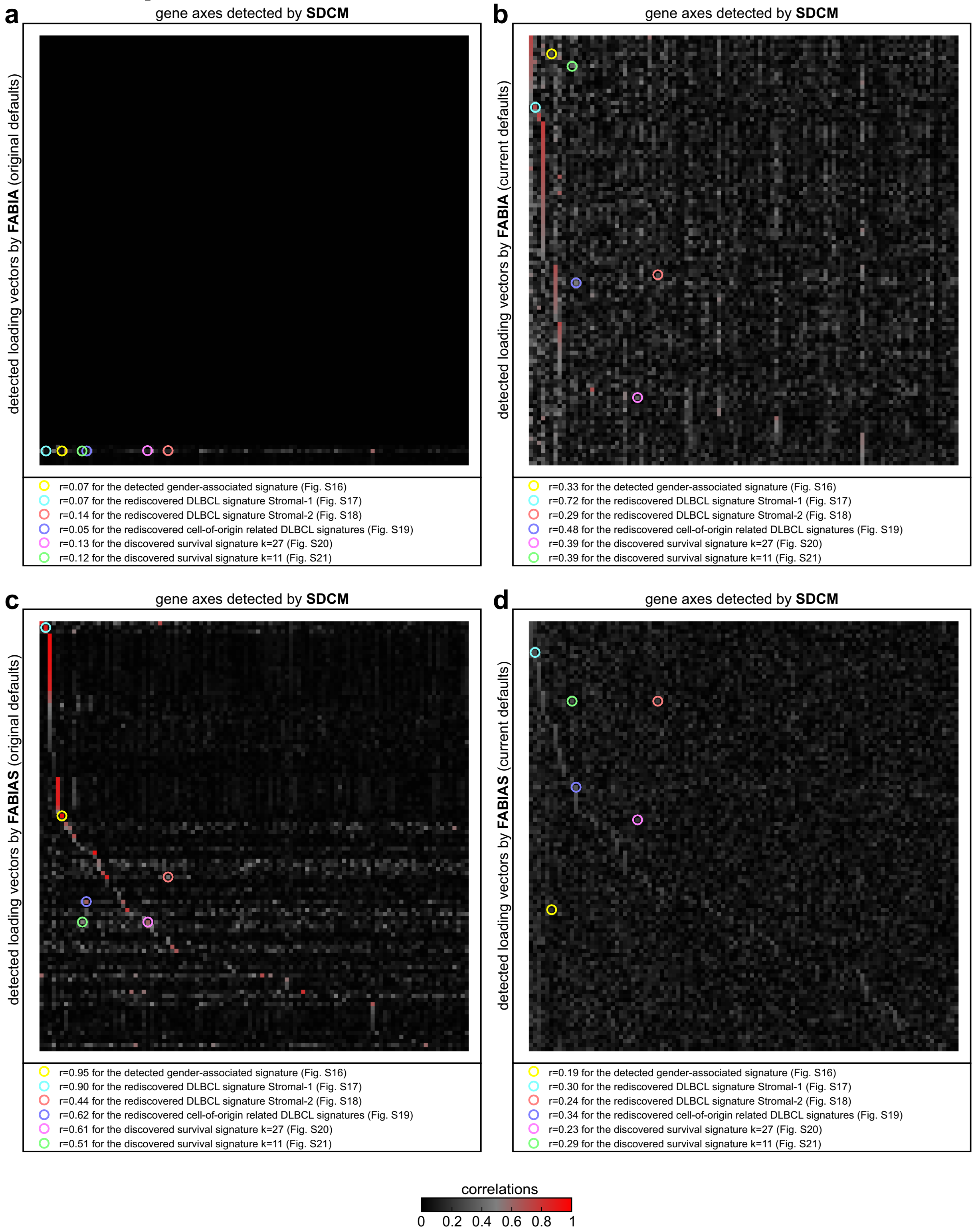

FABIA and FABIAS results for the detection cohort (GSE31312, 54675 transcript clusters, tumor samples from 498 R-CHOP treated patients). **(a)** Results for FABIA using original default parameters[33] ($\alpha = 0.4$, cyc $= 200$, spl $= 1$, spz $= 1$). Detected column vectors in FABIA loading matrices were compared with SDCM gene axes (Supplementary Note 8). As FABIA needs the number of biclusters in advance, it was tasked to search for 105 signatures, as many as discovered by SDCM. Unfortunately, most returned loading vectors for this FABIA configuration were exactly zero (as confirmed on two independent workstations). **(b)** FABIA results using defaults according to its R package v2.16 ($\alpha = 0.01$, cyc $= 500$, spl $= 0$, spz $= 0.5$). Similar to results for simulated data

(Supplementary Fig. 4b), results are dominated by multiple redetections of SDCM signatures $k = 1, 4, 7$ and 8 (incomplete dissection, i.e. the same signature is represented multiple times). Except for the Stromal-1 signature ($r = 0.72$), no FABIA vector represented any of the biological key signatures well ($r < 0.5$). **(c)** Results from FABIAS using original default parameters[33] ($\alpha = 0.6$, cyc $= 200$, spz $= 1$). Both the gender-associated signature ($r = 0.95$) and the Stromal-1 signature ($r = 0.90$) were rediscovered with high correlation. Additionally, the cell-of-origin signature ($r = 0.62$) and survival signature $k = 27$ ($r = 0.61$) were redetected with the highest correlations of any comparison method. Similar to (b), results were dominated by multiple rediscoveries of signatures $k = 3$ and $k = 5$. **(d)** FABIAS results for defaults according to its R package v2.16 ($\alpha = 0.1$, cyc $= 500$, spz $= 0.5$). As for simulated data (Supplementary Fig. 4d), signals of SDCM signatures were split over many detected biclusters; additionally, no bicluster represented any signature discovered by SDCM with high correlation (only gray pixels).

Fig. 24: Method comparison for real data, SDCM versus non-negative matrix factorization

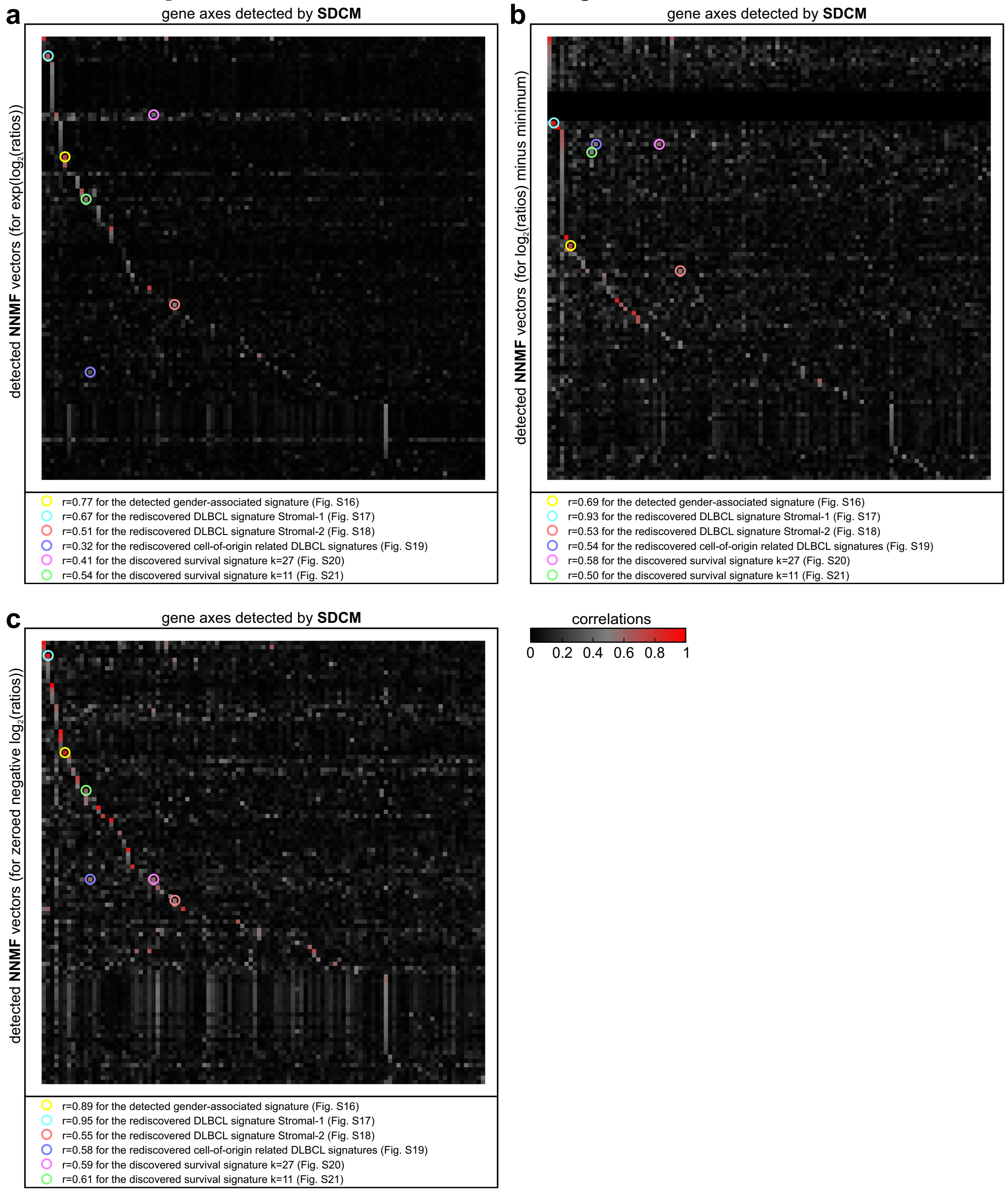

NNMF results for the detection cohort (GSE31312, 54675 transcript clusters, tumor samples from 498 R-CHOP treated patients). **(a)** To transform simulated data to positive numbers, as required by NNMF, we used here exp(measured $\log_2$(ratios)). The number of signatures detected by SDCM was provided, as the method requires such input in advance. SDCM gene axes were compared (Supplementary Note 8) with NNMF vectors in gene space (i.e. column vectors of the first NNMF matrix factor, as returned by the nnmf function in MATLAB®). Overall, only few SDCM signatures were redetected and those only with moderate correlations, e.g. the gender-associated signature with $r = 0.77$. **(b)** NNMF results for measured $\log_2$(ratios) minus their minimum to transform data to positive numbers. Here, the Stromal-1 signature was redetected with high correlation ($r = 0.93$). Similar to FABIA

(Supplementary Fig. 23b), SDCM signatures $k = 1$ and $k = 4$ were detected multiple times with decreasing correlation (incomplete dissections of the underlying signature signal). **(c)** NNMF results after zeroing negative $\log_2$(ratios) to transform measured data to positive numbers as required by NNMF. This NNMF configuration redetected more SDCM signatures than (a) and (b) with good correlations to the gender-associated signature ($r = 0.89$) and to the Stromal-1 signature ($r = 0.95$) and moderate correlations to the other four biological signatures.

Fig. 25: Overview of fitted survival models

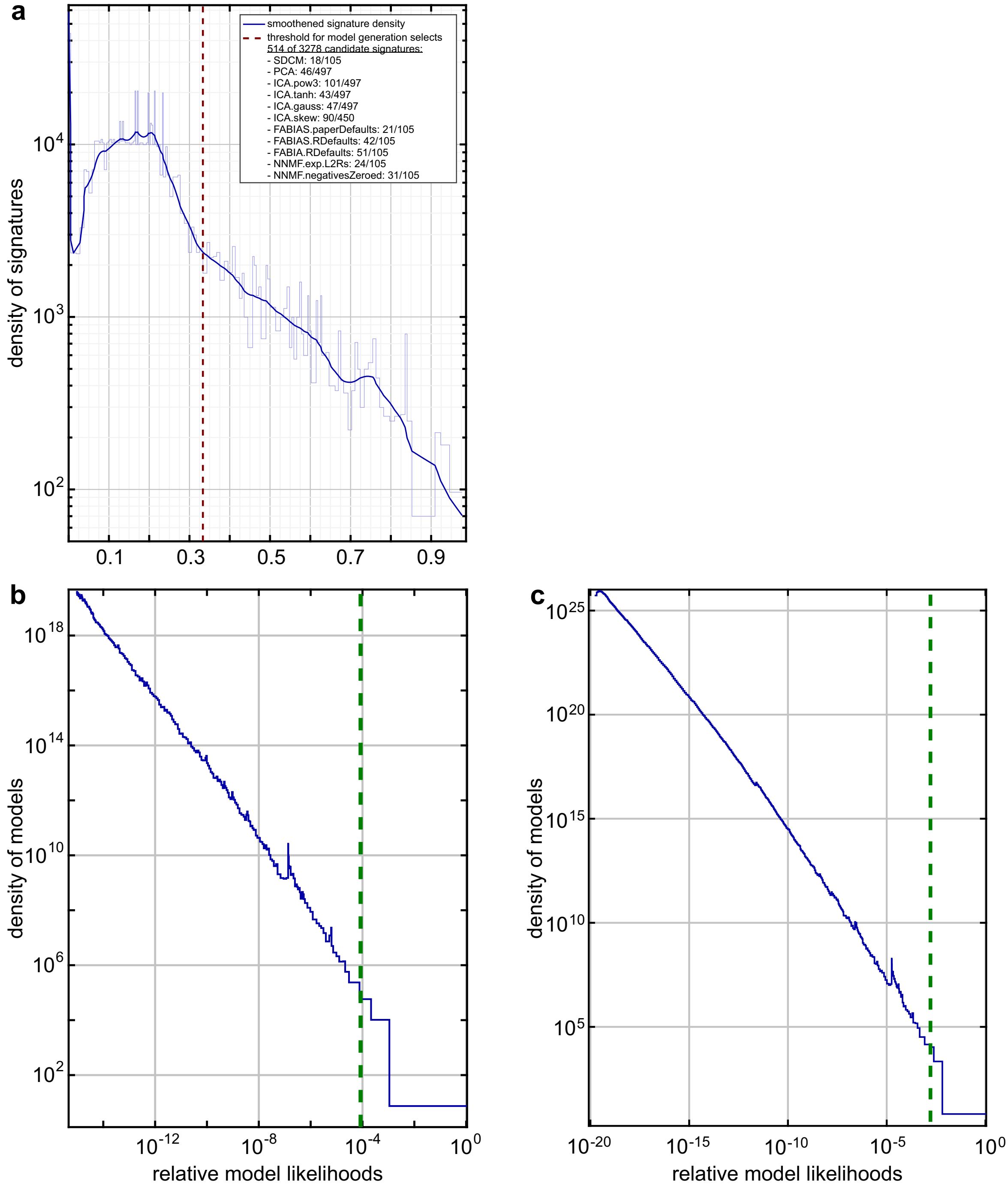

(a) Selection of candidate signatures for model generation. All 3278 signatures detected by SDCM or by any of the comparison methods are distributed over a measure of affected samples in the detection cohort GSE31312. More precisely, we depict the ratios of sample strengths $\langle \mathbf{s}|\mathbf{a} \rangle$ in the respective gene axis (or PC, IC, NNMF or FABIA/S loadings vector) that exceed one standard deviation of all sample strengths in any signature. As suggested by the distribution form and to enable unbiased screening of survival models based on combinations of up to three signatures, we filtered out the 514 top signatures (see legend) affecting at least one third of all samples (red line). **(b)** Distribution of likelihoods including all fitted 131,841 bi-variate CPH models, relative to the best model (cf. Note 14). The top 25 bi-variate models (right of green line) are listed in Table 4a. **(c)** Distribution of relative likelihoods (13, §2.8) including all 22,500,864 fitted tri-variate CPH models (cf. Note 14). The top 25 tri-variate models (right of green line) are listed in Table 4b.

Fig. 26: Discovered survival signature *k*=27

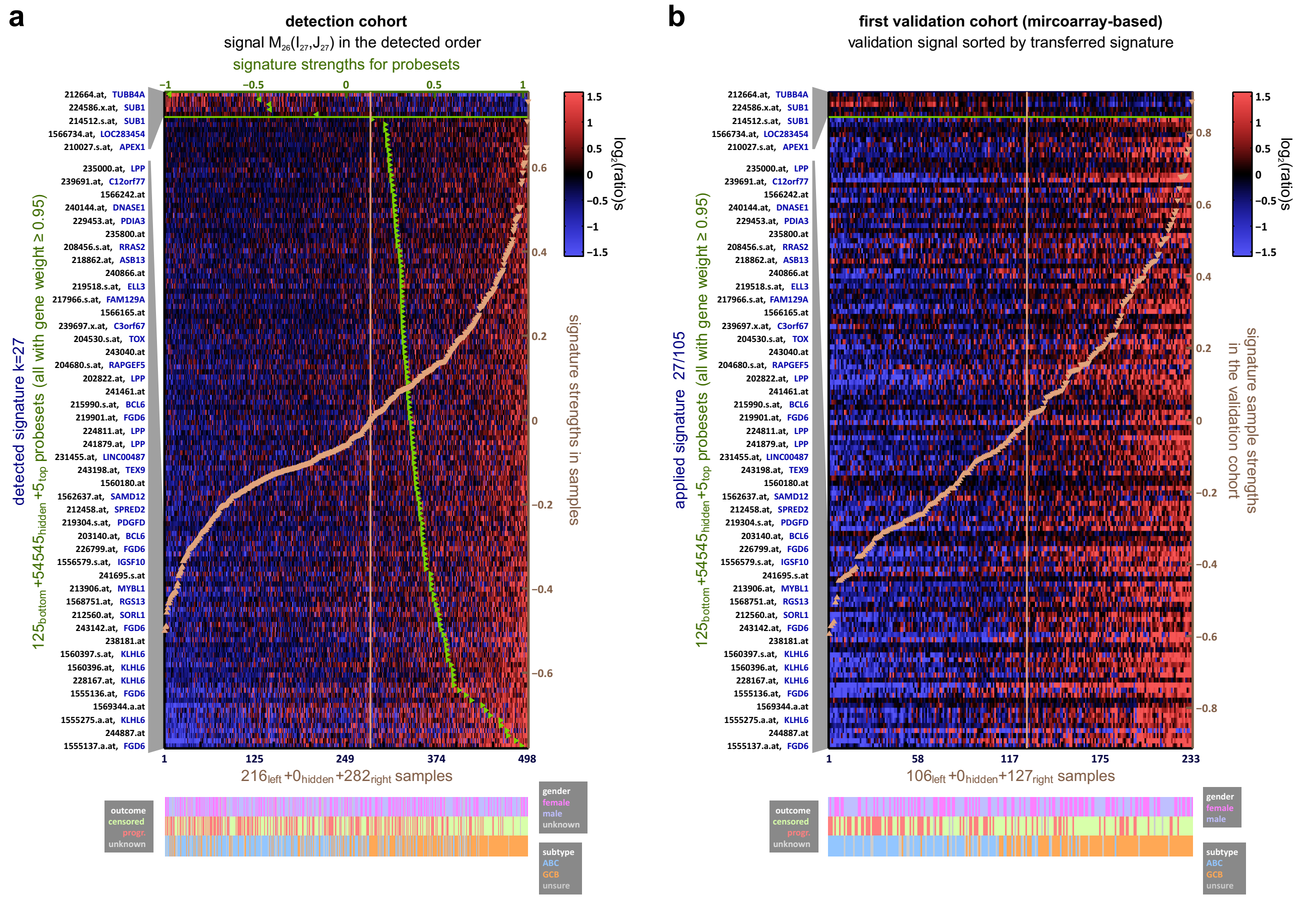

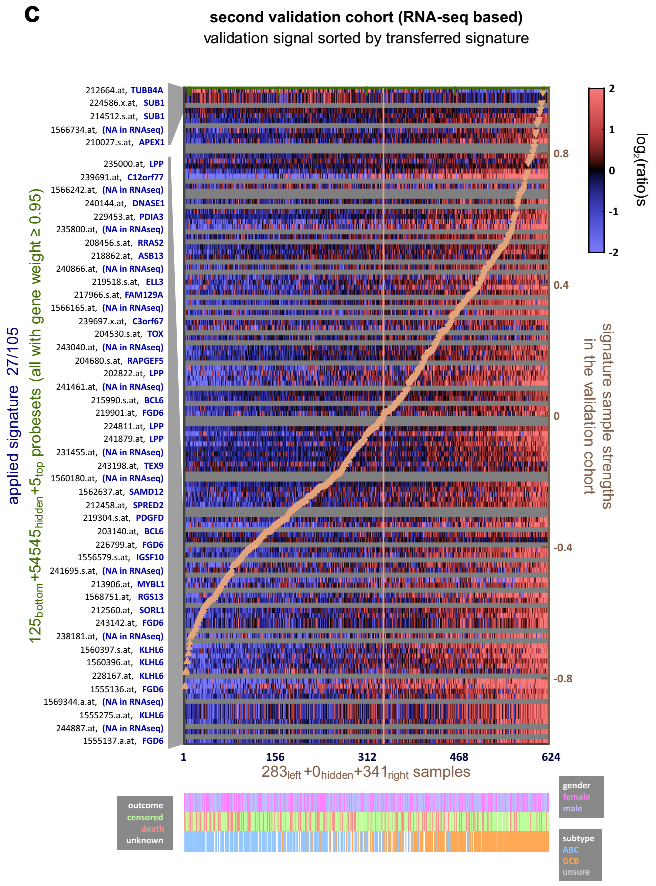

(a) A predominantly one-sided signature was detected in iteration $k = 27$; it is presented as described for the gender-associated signature (Supplementary Fig. 16). **(b)** The signal for the transferred signature in the validation cohort is depicted (Supplementary Note 11). As for the gender-associated signature, signal strengths of transcript clusters differed, but their correlations were retained, validating the discovered signature on GE level. **(c)** The signal for the transferred signature in the RNA-sequencing based add-on validation cohort. Affymetrix probe sets without a match on gene level are depicted in gray (Supplementary Note 11).

ABC DLBCL cases showed a higher density at lower sample strengths and GCB DLBCL cases had a higher density at high sample strengths in all three cohorts. Consistent with known differences in average survival of DLBCL subtypes, a clear survival trend is visible in indicator bars for disease outcome in all cohorts.

Fig. 27: Discovered survival signature *k*=11

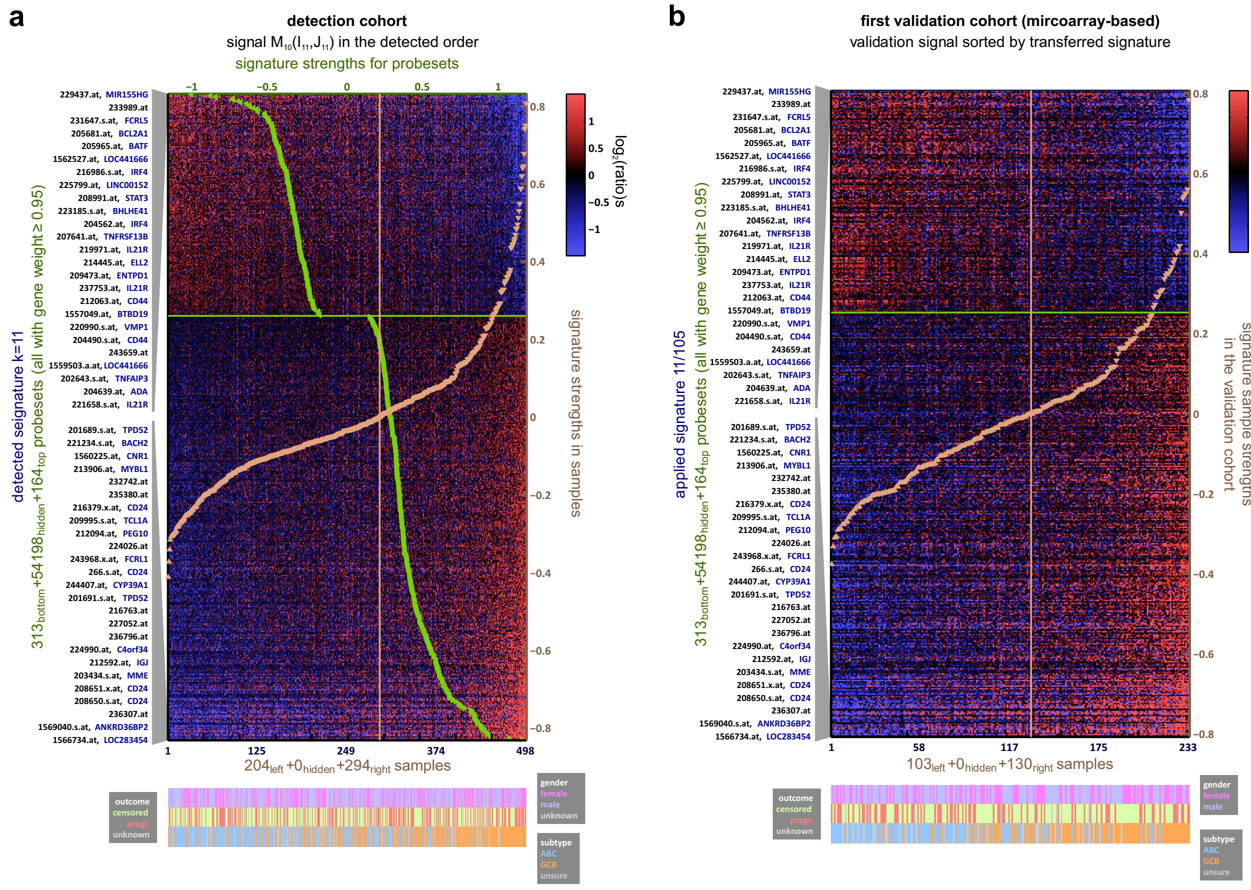

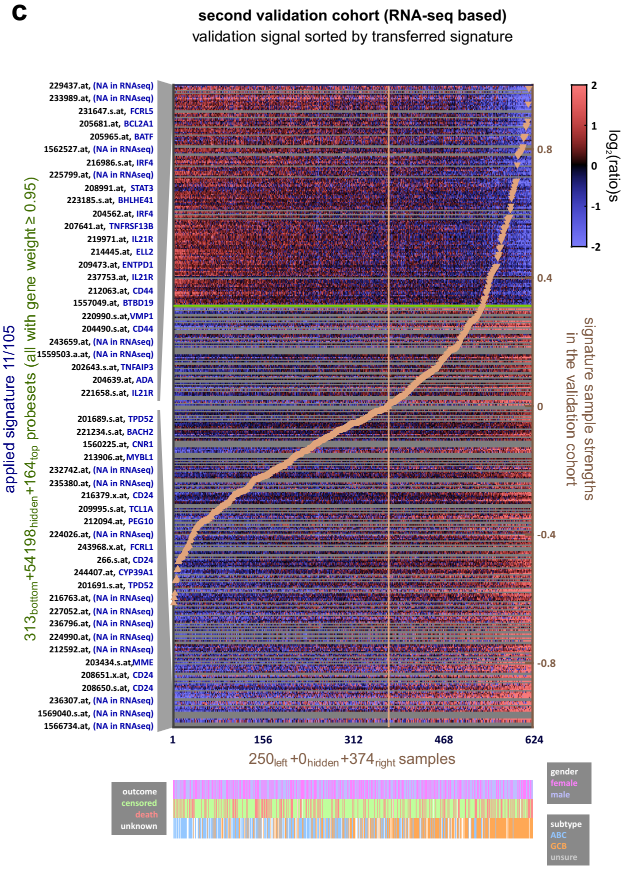

(a) A two-sided signature was detected in iteration $k = 11$. It is presented as described for the gender-associated signature (Supplementary Fig. 16). **(b)** The signal for the same signature in the validation cohort is depicted (Supplementary Note 11). Correlations were retained, which validated this signature on GE level. **(c)** The signal for the same signature in the RNA-sequencing based second validation cohort. Affymetrix probe sets without a match on gene level are depicted in gray (Supplementary Note 11).

As for signature $k = 27$ (Supplementary Fig. 26), ABC DLBCL cases showed a higher density at lower sample strengths and GCB DLBCL cases had a higher density at high sample strengths in all three cohorts. Unlike signatures $k = 27$ and $k = 12$ (Supplementary Fig. 19), no clear survival trend is directly visible in indicator bars for disease progression. However, an unusually high density of GCB DLBCL cases with progressions or observed deaths can be seen at high sample strengths. These events support the synergistic prediction of survival when combining signatures $k = 27$ and $k = 11$ (see main text).

Fig. 28: Survival signature based on IC#54 detected by ICA.gauss

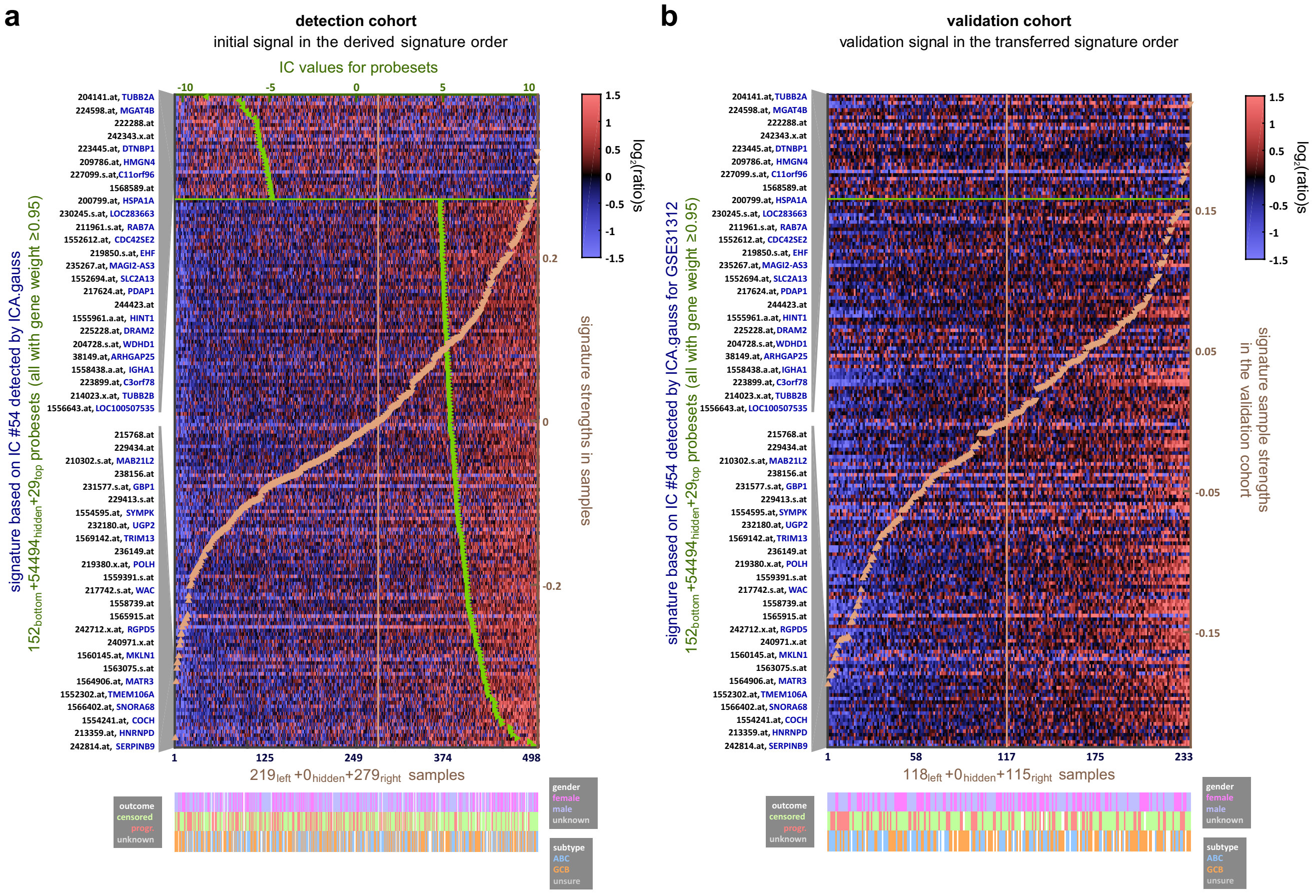

(a) A primarily one-sided signature was detected by ICA.gauss as 54th IC. Besides SDCM signatures $k = 27$ and $k = 11$, this signature is part of the globally best tri-variate CPHM. Gene and sample orders for representing this signature were determined by IC probeset values and sample strengths, respectively (i.e. weighted projections of samples on this IC). Depicted are initial $\log_2$(ratios) for all probesets having a weight ≥ 0.95 (Supplementary Eqn. 15) and for all samples. This signature is associated with a visible survival trend in the learning cohort with less hazards for higher expression of top correlated genes at the bottom (see red/green indicator bar below the heatmap). **(b)** The signal for the same signature is depicted for the validation cohort. Correlations were retained for many genes, validating the existence of this DLBCL signature on GE level. However, the clear survival trend is no longer visible in this independent validation cohort; this was confirmed by Kaplan-Meier analyses (cf. Supplementary Fig. 29).

Fig. 29: Survival predictor performance and generalizability for the tri-variate model

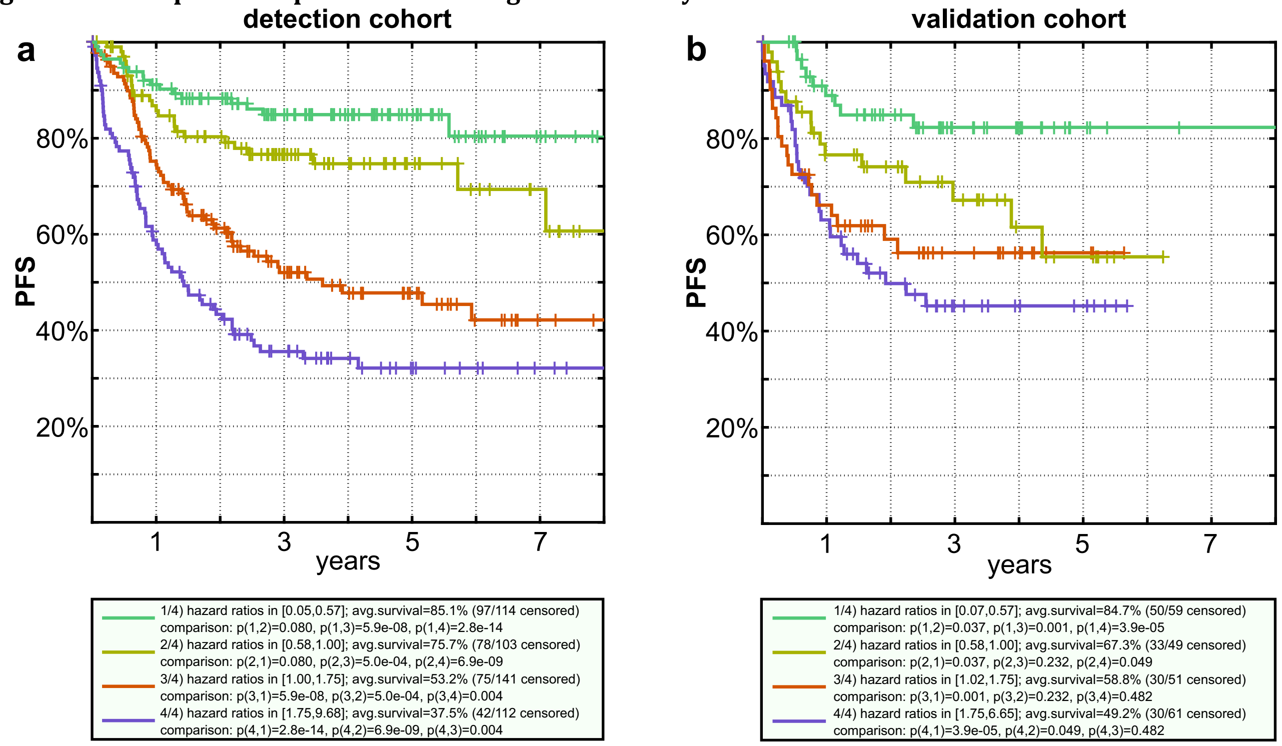

Kaplan-Meier estimates visualize survival differences revealed by the top 3-signature model based on signatures SDCM#27, SDCM#11 and ICA.gauss#54 (cf. Note 14 and Table 4). **(a)** Progression-free survival for molecular risk partitions (of ≥ 1.75, ≥ 1, $\geq 1/1.75$ and $< 1/1.75$ times the cohort average risk) is shown for the detection cohort (GSE31312, follow-up events available for 470 R-CHOP treated patients). Differences between most partitions were significant and the second and third risk partitions show a clear survival difference. Average survival per partition and p-values for comparisons between partitions (log-rank tests) are provided in the legend. **(b)** In the validation cohort (GSE10846, PFS events available for 220 R-CHOP treated patients), differences in progression-free survival between all non-neighboring risk partitions remained significant. However, model generalizability was lower than expected, as clear distinctions between the second and third as well as between the third and fourth risk partitions were lost in the validation cohort. This may indicate that this tri-variate predictor is over-fitted.

Fig. 30: Survival predictor performance and generalizability for the bi-variate model

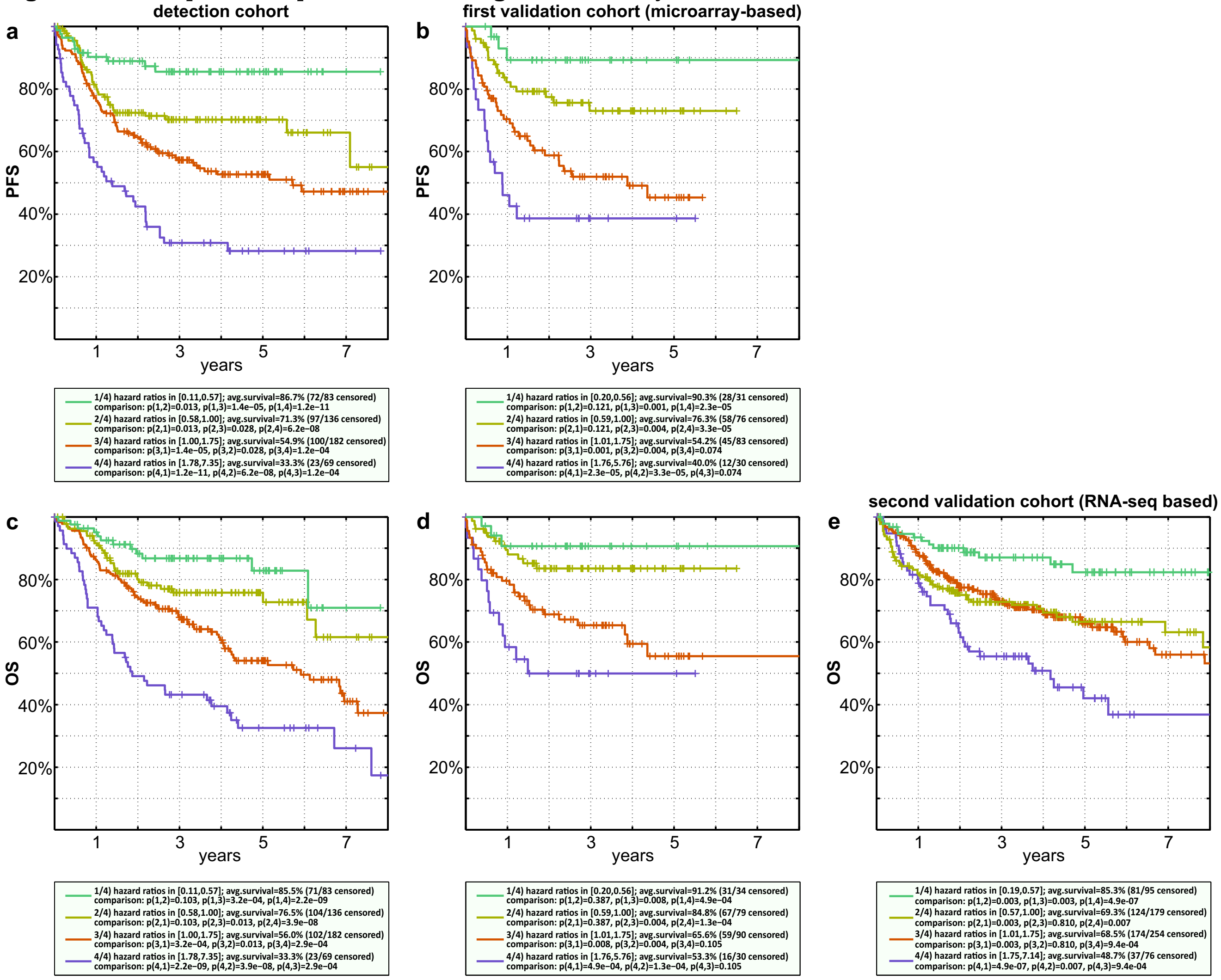

Kaplan-Meier estimates visualize survival differences revealed by the top 2-signature model based on signatures SDCM#27 and SDCM#11. **(a)** Progression-free survival for molecular risk partitions (of ≥ 1.75, ≥ 1, ≥ 1/1.75 and < 1/1.75 times the cohort average risk) is shown for the detection cohort (GSE31312, follow-up events available for 470 R-CHOP treated patients). Differences between any two partitions were significant. Average survival per partition and *p*-values for comparisons between partitions (log-rank tests) are provided in the legend. **(b)** In the validation cohort (GSE10846, PFS events available for 220 R-CHOP treated patients), differences in progression-free survival of about the same magnitude were revealed by our 2-signature predictor, confirming its generalizability. All non-neighboring risk partitions were significantly different. **(c, d, e)** Overall survival for the same molecular risk partitions was significantly different between all non-neighboring risk partitions in both the detection and validation cohort. In the RNA-sequencing based second validation cohort, all but the two center partitions showed significantly different overall survival.

(Available PFS data for GSE31312 did not count late deaths as treatment failures, as they were assumed to be unrelated to the underlying lymphoma or its treatment[3]. Hence, OS curves may be lower than respective PFS curves for later times. Clinical data for GSE10846 considered every observed death as a progression; thus, PFS is ≤OS always for this cohort. No PFS data was available for the second validation cohort EGAS00001002606.)

Fig. 31: Generalizability of survival differences between DLBCL subtypes

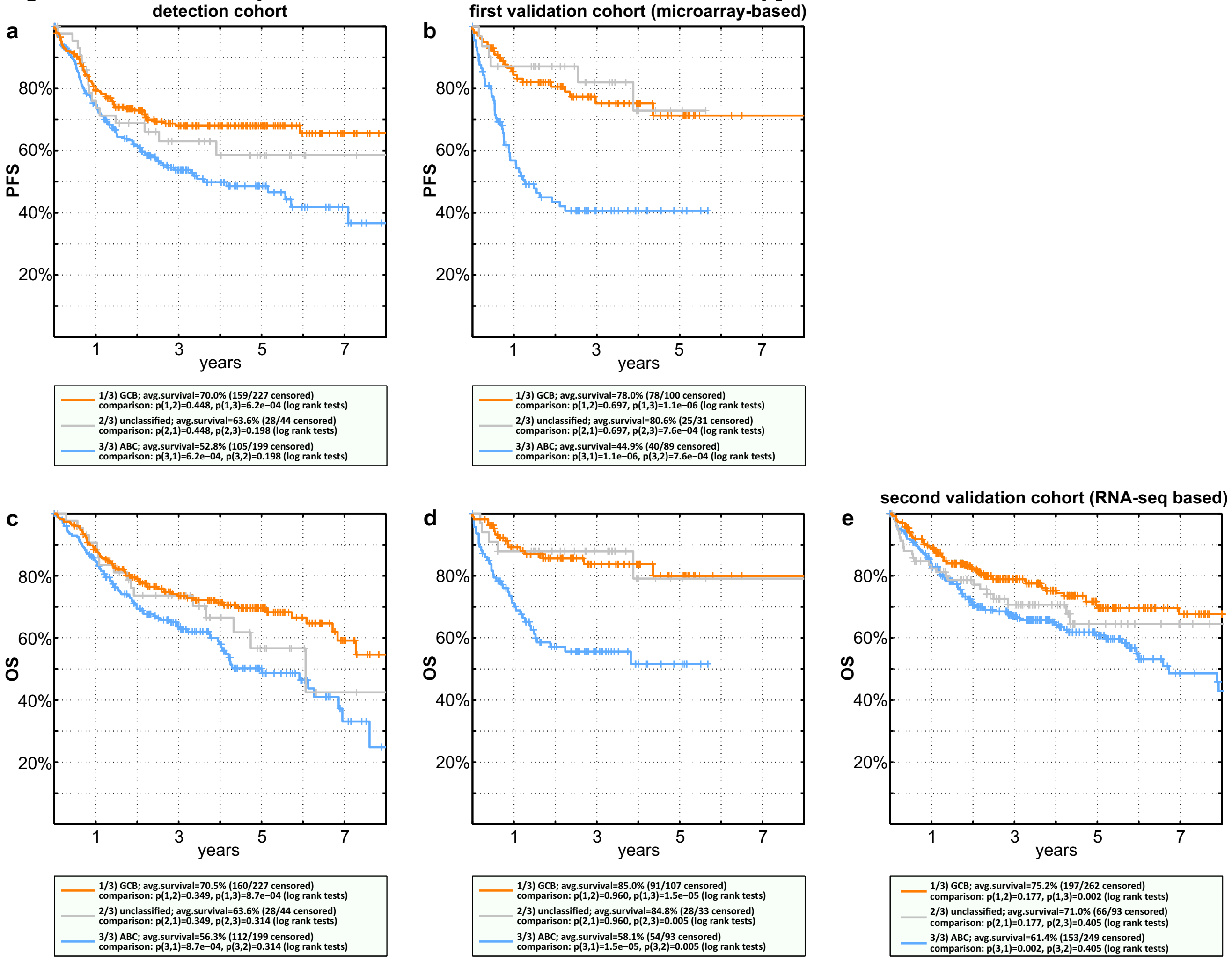

To compare the generalizability of our predictor model with that of DLBCL subtypes, we partitioned samples by subtypes (as published with original datasets). **(a)** PFS differences between DLBCL subtypes in the newer cohort (GSE31312, 470/498 R-CHOP treated patients with PFS data) showed a 5-years gap of only 19%. Detailed statistics and *p*-values for comparisons between subtypes (log-rank tests) are provided in the legend. **(b)** PFS differences between DLBCL subtypes in the older cohort (GSE10846, 220/233 R-CHOP treated patients with PFS data) showed a much larger 5-years gap of 30%. **(c, d, e)** OS differences between DLBCL subtypes revealed similar discrepancies. Published subtypes seem to be optimally suited for explaining survival differences in cohort GSE10846, but this did not generalize well to cohort GSE31312 and neither to the RNA-sequencing based addon validation cohort (EGAS00001002606, 604/624 R-CHOP treated patients had OS follow-up data, no PFS available for this cohort).

Fig. 32: Predictions within DLBCL subtypes, progression-free survival

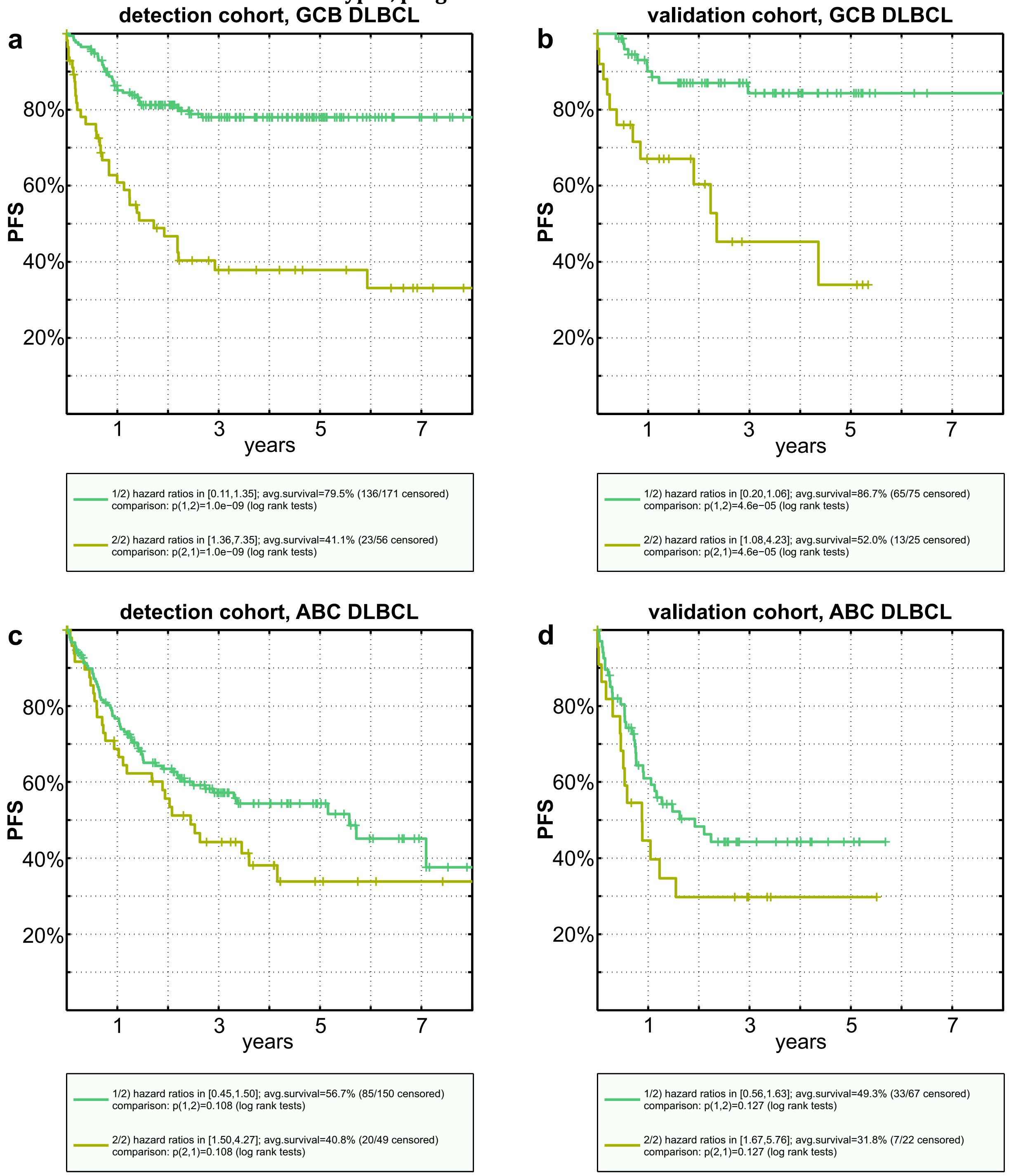

To test if discovered survival differences affect known DLBCL subtypes differently, we applied our predictor separately to ABC DLBCL and GCB DLBCL cases, as annotated in GEO accessions GSE31312 (detection cohort) and GSE10846 (validation cohort). **(a, b)** In both the detection and the validation cohort, PFS for GCB DLBCL was significantly and strongly adverse for patients in the quartile with the highest molecular risks predicted by our 2-signature model. Detailed statistics and p-values (log rank tests) are provided in legends. **(c, d)** For ABC DLBCL cases, a trend towards adverse survival in the high risk quartile was also visible in both cohorts, but it was not significant.

Fig. 33: Predictions within GCB DLBCL, overall survival

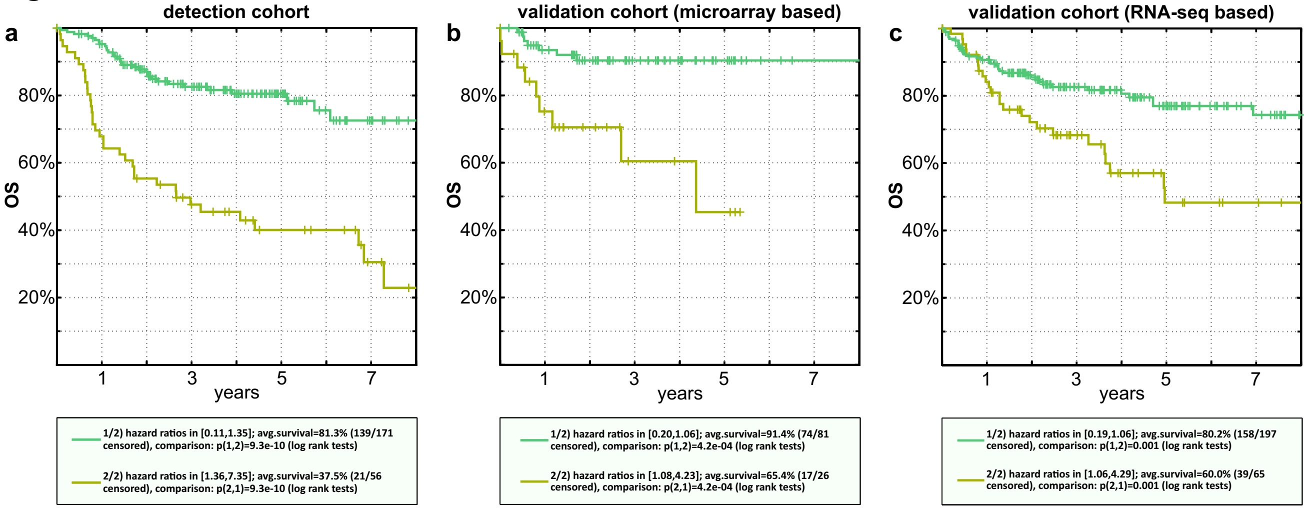

To test generalizability of the predicted subgroup of significantly inferior survival within GCB DLBCL, we additionally analyzed survival differences in a RNA-sequencing based second validation cohort (EGAS00001002606, 604/624 R-CHOP treated patients had OS follow-up data). As no PFS follow-up information was available for this cohort, we again analyzed GCB DLBCL in GSE31312 (microarray-based detection cohort) and GSE10846 (microarray-based validation cohort) with OS for direct visual comparability, i.e. without using information about disease progressions. **(a, b, c)** In the detection and in both validation cohorts, OS for GCB DLBCL remained significantly adverse for patients in the quartile of highest predicted molecular risks by our 2-signature model. Detailed statistics and *p*-values (log rank tests) are provided in legends.

Fig. 34: Risk landscape over sample strengths in discovered survival signatures

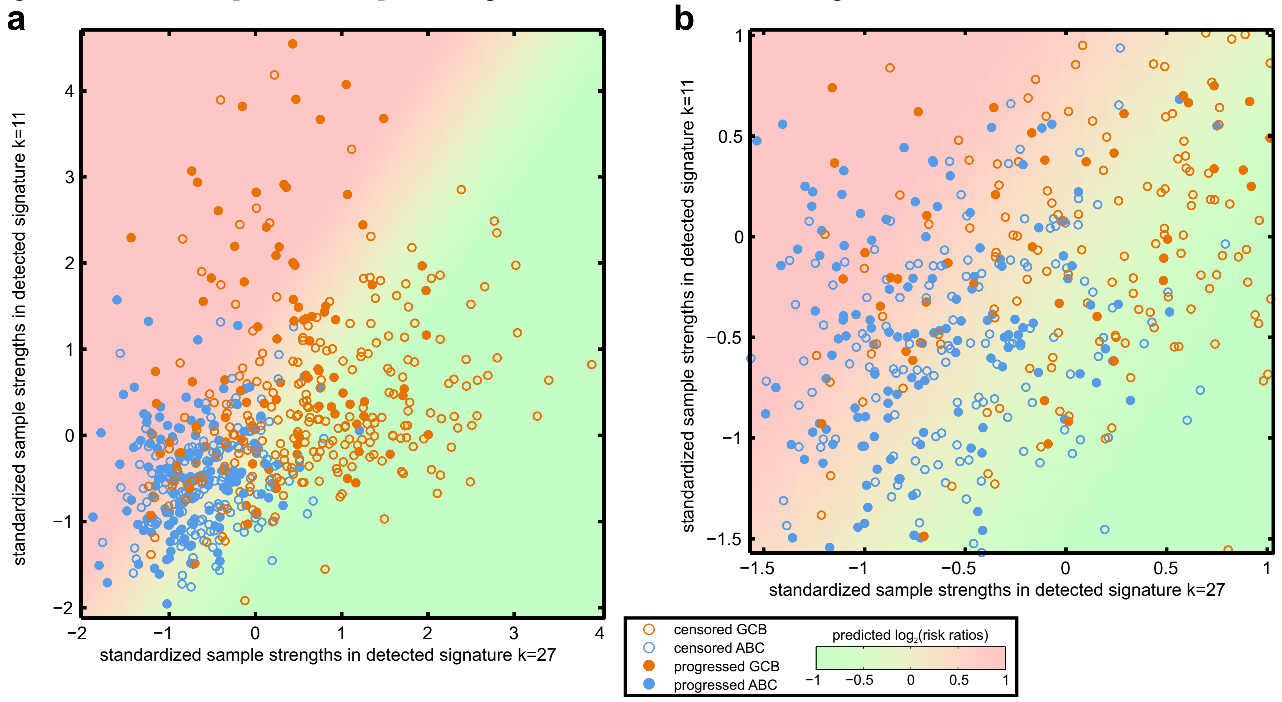

To illuminate the relationship with DLBCL subtypes, we scattered all available samples based on their strengths in both discovered survival signatures (see Supplementary Figs. 26 and 27). Different markers indicate DLBCL subtype and follow-up status (see legend). Background colors depict $\log_2$(risk ratios) according to our 2-signature survival predictor relative to the average risk. **(a)** ABC DLBCL cases (blue) clustered densely in the lower left; this explains why sample strengths of both detected survival signatures are associated with subtype. GCB DLBCL cases (orange) were scattered over a wider range of sample strengths and risks. The survival gradient from red to green indicates that only the relative sample strength in both survival signatures can explain existing survival dependencies precisely. One signature alone and standard subtypes can only capture some parts of this survival gradient and fail to resolve survival differences between GCB DLBCL cases at the top and the right. **(b)** Zooming into sample strengths where ABC DLBCL cases clustered densely, shows that the survival gradient is weaker within ABC DLBCL. However, it is still visible in form of a higher density of progressed events (filled circles) on the upper left compared to the bottom center.

Fig. 35: Predictions within clinical risk classes

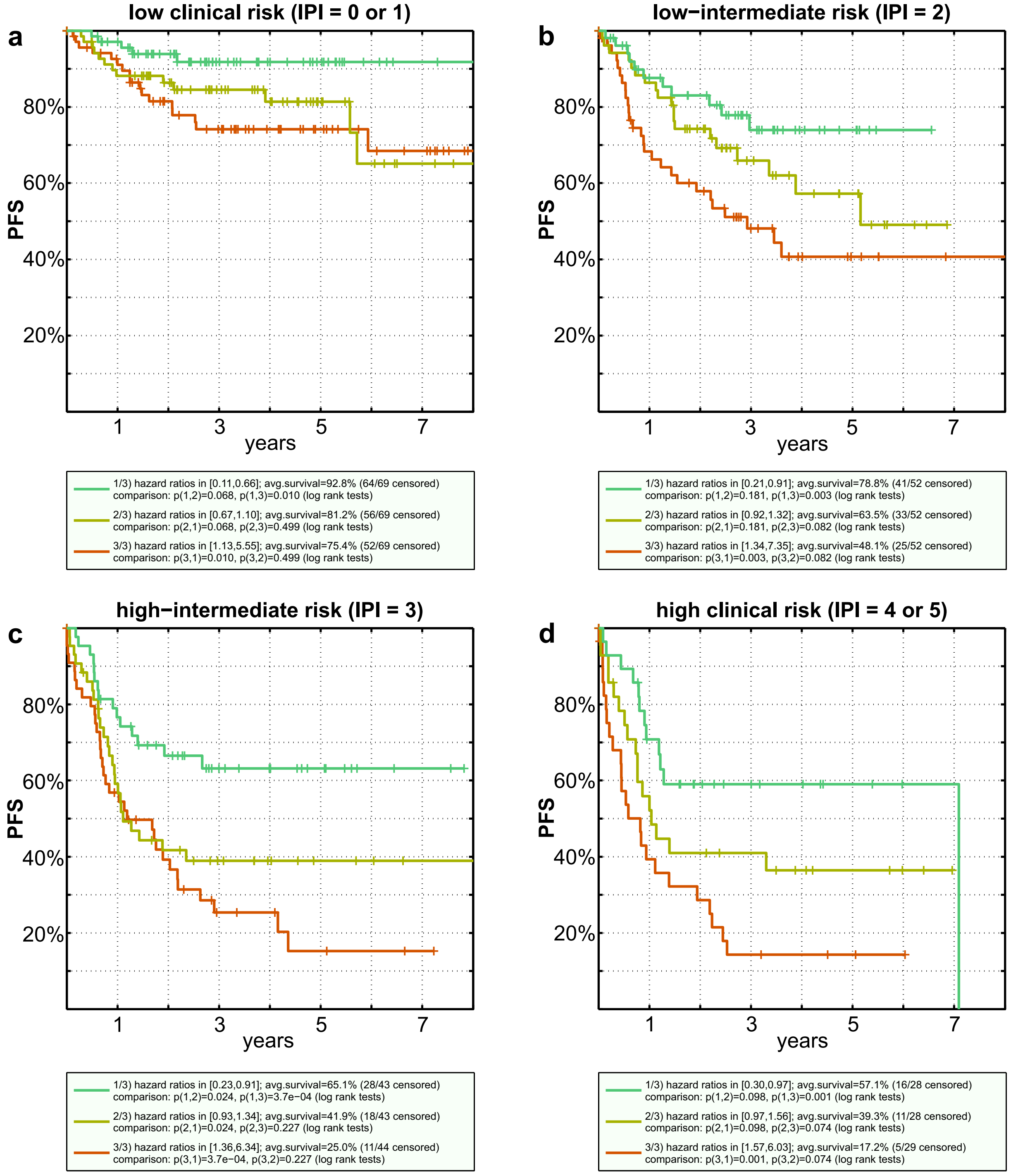

To analyze the relationship between molecular risks predicted by our 2-signature model and macroscopically determined clinical risks, we finally partitioned patients into four clinical risk classes according to their international prognostic index (IPI)[42]. Not all patients had IPI annotations. To retain enough patients in each clinical class for a tercile analysis of their molecular risks, we merged events from both cohorts for this test. **(a)** Of these patients, 207 were in the low clinical risk group. **(b)** 156 were in the low-intermediate risk group. **(c)** 130 were in the high-intermediate risk group. **(d)** 85 were characterized by high clinical risk. As expected, average survival declined with increasing clinical risk. On top of that, our 2-signature model revealed significant survival differences between top and bottom terciles of predicted molecular risks in all four clinical risk classes. Figure legends provide detailed statistics and *p*-values (log-rank tests).

Fig. 36: Gene set enrichment analyses for SDCM signatures k=27and k=11

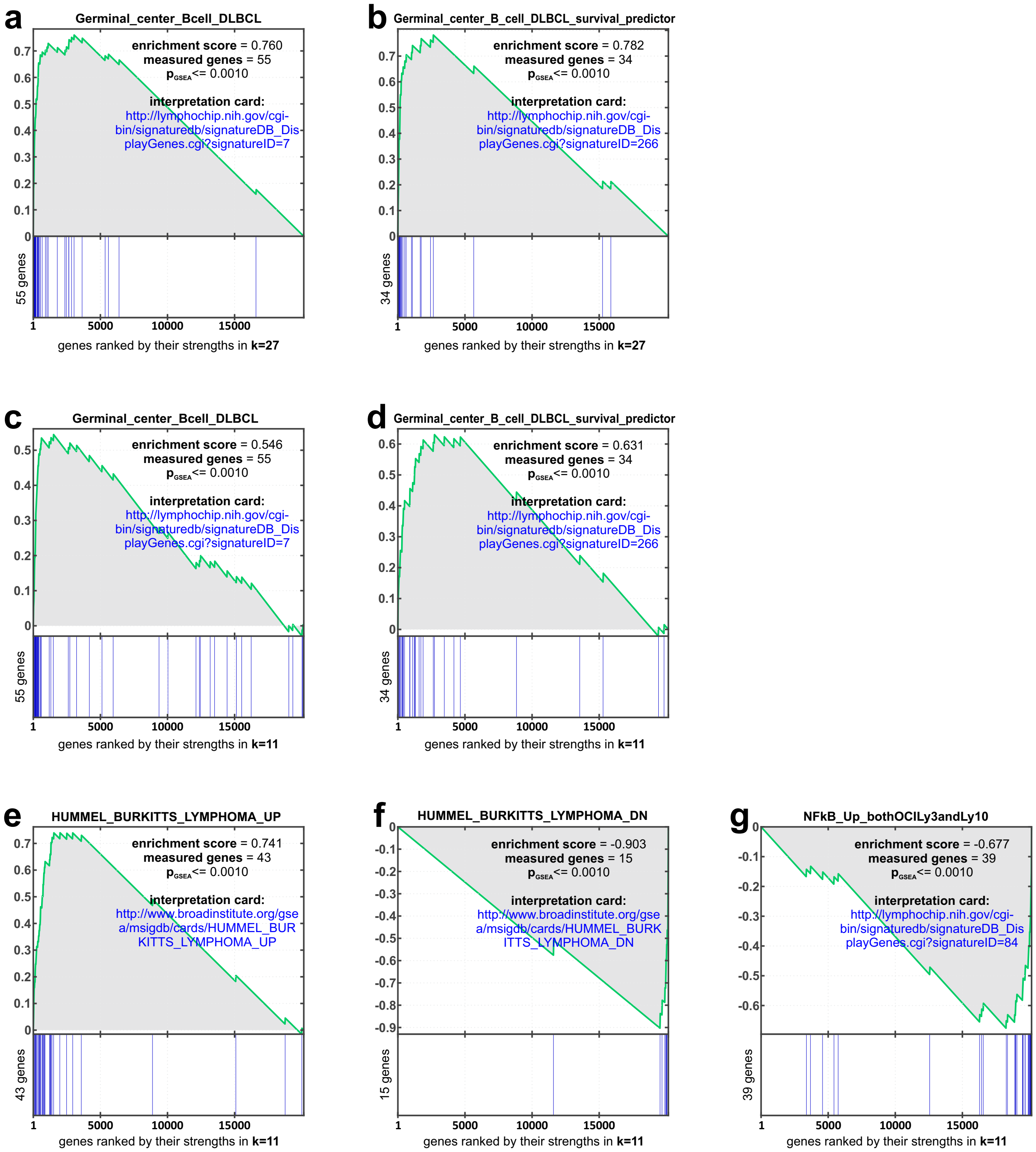

Key results from gene set enrichment analysis characterizing discovered SDCM DLBCL signatures $k = 27$ and $k = 11$ in context of previously published gene signatures. **(a-d)** Enrichment of germinal center (GC) related signatures for $k = 27$ and $k = 11$. Both GC signatures were significantly enriched with comparable scores at top gene ranks of both SDCM signatures, despite their anti-aligned survival associations. As discussed in the main text, previous GC signatures might heterogeneously represent a mixture of different gene expression sources in DLBCL, such as patterns from the cell of origin or stromal influences that SDCM was able to properly dissect by correlation maximization. **(e-g)** Additional significantly enriched gene signatures associating $k = 11$ to a Burkitt lymphoma like gene expression pattern and to activity of the oncogenic NF-κB pathway. In contrast to GC signatures, these signatures were only characteristic for $k = 11$. (See discussion in main text for interpretation.)

Supplementary Tables

Table 1: Simulation parameters for signatures in the versatility test scenario

#	Comments	participating genes (of 1000)			affected samples (of 100)			signal strength	
		+	0	-	+	0	-	max	min
**1**	Technical laboratory effects like different labeling protocols can cause very broad and strong signatures that may outshine smaller signatures of biological origin. Such large laboratory effects are typically easy to detect, but hard to dissect in order to allow detection of initially outshined signatures.	500	0	500	34	0	66	1.5	0
**2**	For example, the gender signature (Supplementary Fig. 16) shows a strong binary signal, but only for very few of all measured genes. Such signatures also represent the prototype of traditional clustering methods that assume large gaps between clusters instead of a gradual transition that is supported by our bimonotonic signature model.	3	990	7	50	0	50	2	2
**3**	This pattern represents biological signatures that are carried by many genes, but that only exists in a subclass of samples; in some stronger in others weaker (gradual transition). The signature is two-sided like the COO-related signature (Supplementary Fig. 19), i.e. it has a large number of both correlated and anti-correlated genes.	50	800	150	33	50	17	2	0.5
**4**	A signature of typical medium signal strength. It is one-sided like the Stromal-1 signature (Supplementary Fig. 17), i.e. it has no anti-correlated genes.	100	900	0	34	0	66	1	0.5
**5**	A narrow signature of typical medium strength. To test detection specificity on gene level, only one of 20 participating genes is positively correlated.	1	980	19	25	50	25	1	0.5
**6**	A signature of symmetric shape that has a very weak signal to test detection sensitivity limits. Its maximum signal of 0.5 just equals the standard deviation of the simulated noise.	50	900	50	50	0	50	0.5	0
**7**	A very narrow signature that only exists in 5% of all simulated samples. It could mimic a rare disease subtype. As the signature signal is comprised exclusively of positive simulated $\log_2$(ratios), it allows testing detection and dissection of high-dimensional expression offsets (analogous to the magenta 3D interaction in Fig. 1).	250	750	0	5	95	0	2	1

For method validation and comparison, we simulated signals of $m = 1000$ genes and $n = 100$ samples containing signatures inspired by different real-world patterns. Each signature has a bimonotonic signal (see Fig. 3a). For each simulation of the versatility test, participating genes, affected samples and signature orders for which the signature signal is bimonotonic were selected randomly. Except for signature #1 that affects all genes and samples, signatures have a non-zero signal only within random subspaces of the gene and sample space. Counts of participating correlated genes or samples are defined in (+)-columns; (-)-columns define counts of anti-correlated genes or samples. The minimum and maximum of absolute signal strengths of bimonotonic signature signals are defined in the last two columns. (The simulated noise had a standard deviation of 0.5.)

Table 2: Performance comparison of SDCM with biclustering methods

Method	M0		M1		M2		M3	
	**rank**	**s**	**rank**	**s**	**rank**	**S**	**rank**	**s**
SDCM sensitive	1	0.63	1	0.18	1	0.31	1	0.56
SDCM	2	0.61	3	0.14	3	0.23	3	0.49
FABIAS	3	0.56	2	0.15	2	0.27	2	0.55
FABIA	4	0.48	4	0.11	4	0.20	4	0.48
ISA_1	5	0.33	9	0.04	9	0.03	7	0.14
ISA_2	6	0.30	6	0.08	6	0.14	5	0.23
ISA_3	7	0.19	7	0.04	8	0.04	8	0.14
PLAID_ms_5	8	0.08	5	0.10	5	0.14	6	0.22
MFSC	9	0.06	20	0.00	20	0.00	20	0.00
PLAID_t_ab	10	0.05	11	0.02	14	0.00	11	0.02
PLAID_ss	11	0.04	8	0.04	7	0.04	9	0.07
PLAID_t_a	12	0.04	10	0.03	11	0.01	10	0.05
SPEC_2	13	0.03	19	0.00	19	0.00	19	0.00
OPSM	14	0.01	13	0.01	12	0.01	13	0.01
SAMBA	15	0.01	15	0.00	15	0.00	15	0.00
FLOC	16	0.01	14	0.01	13	0.00	14	0.00
BIMAX	17	0.00	12	0.01	10	0.01	12	0.01
XMOTIF	18	0.00	16	0.00	16	0.00	16	0.00
CC	19	0.00	17	0.00	17	0.00	17	0.00
SPEC_1	20	0.00	18	0.00	18	0.00	18	0.00

We applied SDCM to four previously published benchmarks[3], each consisting of 100 datasets. M0 is comprised of multiplicatively generated datasets. M1 to M3 were simulated with an additive data model for increasing signal-to-noise ratios. Detected signatures were reduced to biclusters by cutting their extended signature focus ($|\mathbf{v}^{g}\rangle$, $|\mathbf{v}^{s}\rangle$) at 0.6 (e.g., a gene with $\langle \mathbf{e}_i^{g} | \mathbf{v}^{g} \rangle \geq 0.6$ is in the bicluster, all others are out; likewise for samples). Then, we computed consensus scores[3] relative to true simulated biclusters, as was previously done for several other biclustering methods (13 base methods; different configurations are named as before[3]). Published biclustering results for all these other methods were downloaded from www.bioinf.jku.at/software/fabia/benchmark.html and their consensus scores were recomputed and confirmed with the identical function that we also used to score SDCM results. In our default configuration, SDCM ranked first or second in each benchmark M0-M3. It ranked first for all four benchmarks when we configured more sensitive qualification thresholds ($\alpha_{\mathrm{r}} = 10^{-8}/(m+n)$ and $\alpha_{\mathrm{s}} = 10^{-3}/(m+n)$; the divisor corresponds to Bonferroni correction again, cf. default qualification thresholds in Supplementary Note 4); we chose more conservative default thresholds, as signatures near the noise level are typically not of interest for biological or medical research of real datasets).

Table 3: Performance comparison between SDCM and PCA

		Gene axes correlations				Sample axes correlations			
simulated signature index	pattern index (see Fig. 3a)	Sensitivity p-values		Specificity p-values		Sensitivity p-values		Specificity p-values	
		SDCM	PCA	SDCM	PCA	SDCM	PCA	SDCM	PCA
1	1	0.9963	0.0037	1	7.20E-24	1	1.90E-05	1	1.10E-17
2	2	1.60E-08	1	2.80E-24	1	3.60E-22	1	1.00E-21	1
3	3	1.20E-12	1	0.0029	0.9971	6.70E-18	1	5.40E-05	0.9999
4	4	3.80E-17	1	9.50E-12	1	1.70E-26	1	2.80E-23	1
5	5	0.3803	0.6197	1.80E-17	1	2.50E-15	1	5.20E-16	1
6	6	0.9999	7.60E-05	7.60E-13	1	0.3351	0.6649	1.80E-10	1
7	7	2.30E-09	1	7.10E-10	1	0.7967	0.2033	4.60E-17	1
8	2	2.40E-10	1	3.20E-26	1	1.30E-22	1	3.40E-19	1
9	3	3.80E-12	1	1.50E-05	1	3.60E-15	1	8.50E-07	1
10	4	1.50E-13	1	1.60E-09	1	3.10E-19	1	1.30E-20	1
11	2	1.50E-08	1	6.80E-22	1	9.20E-23	1	5.20E-19	1
12	3	4.70E-12	1	0.0013	0.9987	2.50E-19	1	2.60E-11	1
13	4	9.00E-17	1	7.60E-24	1	1.40E-27	1	2.50E-18	1

We compared the detection performance of SDCM and PCA based on achieved correlations between simulated and detected gene axes in all 49 repetitions of the 13-signature versatility test (Supplementary Fig. 9).

To compare detection sensitivity for each simulated signature, we asked whether the best-matching gene axis detected by SDCM or the best matching PC detected by PCA showed a significantly higher correlation to the correct simulated gene axis (one-tailed paired t-tests of 49:49 absolute correlations). To compare detection specificity for each simulated signature, we first determined unspecific correlations for each detected gene axis respectively PC. As measure, we utilized the means of their absolute correlations to all other simulated gene axes, except for their best matching one. Then we asked which method had significantly lower unspecific correlations (again using one-tailed t-tests paired by the 49 simulation runs).

To quantify and compare the correct extraction of top genes that participate in a simulated signature, we computed correlations of gene axes in the gene space. Analogously, the correct detection of samples that are influenced by a simulated signature was measured in the sample space by correlating simulated with detected sample axes respectively PCs (Supplementary Note 8). The table lists one-tailed p-values of all comparisons in both the gene and sample space for each of the 13 signatures.

PCA showed significantly better sensitivity (i.e. higher correlation to the correct simulated signature axes) and better specificity (i.e. lower correlation to all other simulated signature axes) for signature #1. Results were mixed for signature #6. For all other signatures, SDCM was significantly more sensitive and significantly more specific.

Table 4: Top survival models

(a) Top 25 bi-variate survival models (cf. Note 14)				
**rank**	**explanatory CPHM variables detected by**	**indices in detection method context**	**AIC** (13, §2.2)	**relative model likelihood** (13, §2.8)
1	SDCM, SDCM	[11 27]	1992.39	1.00
2	SDCM, ICA_gauss	[27 54]	2000.65	0.02
3	SDCM, FABIAS_RDefaults	[27 8]	2000.95	0.01
4	SDCM, ICA_tanh	[12 58]	2002.48	6.4E-3
5	PCA, ICA_gauss	[5 54]	2005.04	1.8E-3
6	SDCM, ICA_skew	[12 183]	2005.37	1.5E-3
7	SDCM, FABIAS_paperDefaults	[27 60]	2005.59	1.4E-3
8	SDCM, ICA_skew	[27 35]	2005.92	1.2E-3
9	SDCM, ICA_pow3	[27 130]	2006.15	1.0E-3
10	SDCM, FABIA_RDefaults	[27 5]	2006.44	8.9E-4
11	SDCM, ICA_pow3	[27 114]	2007.49	5.3E-4
12	SDCM, ICA_gauss	[12 54]	2008.15	3.8E-4
13	NNMF_exp_L2Rs, NNMF_negativesZeroed	[26 8]	2008.77	2.8E-4
14	SDCM, PCA	[27 8]	2009.06	2.4E-4
15	ICA_tanh, ICA_gauss	[13 54]	2009.09	2.4E-4
16	SDCM, PCA	[27 12]	2009.12	2.3E-4
17	SDCM, FABIA_RDefaults	[12 91]	2009.18	2.3E-4
18	SDCM, ICA_tanh	[27 13]	2009.39	2.0E-4
19	SDCM, NNMF_negativesZeroed	[11 46]	2009.45	2.0E-4
20	SDCM, FABIAS_paperDefaults	[11 60]	2010.53	1.1E-4
21	NNMF_exp_L2Rs, NNMF_negativesZeroed	[26 4]	2010.54	1.1E-4
22	ICA_tanh, ICA_skew	[13 183]	2010.77	1.0E-4
23	SDCM, ICA_tanh	[27 58]	2010.82	9.9E-5
24	SDCM, ICA_skew	[27 183]	2010.94	9.4E-5
25	ICA_tanh, ICA_tanh	[13 58]	2011.14	8.5E-5
**(b) Top 25 tri-variate survival models** (cf. Note 14)				
**rank**	**explanatory CPHM variables detected by**	**indices in detection method context**	**AIC** (13, §2.2)	**relative model likelihood** (13, §2.8)
1	SDCM, SDCM, ICA_gauss	[11 27 54]	1972.52	1.00
2	SDCM, ICA_gauss, FABIAS_RDefaults	[27 54 8]	1976.01	0.17
3	SDCM, ICA_gauss, FABIAS_paperDefaults	[12 54 29]	1978.09	0.06
4	SDCM, PCA, ICA_gauss	[27 5 54]	1979.90	0.02
5	SDCM, SDCM, ICA_gauss	[7 12 54]	1981.11	0.01
6	SDCM, ICA_gauss, NNMF_negativesZeroed	[12 54 33]	1981.83	9.5E-3
7	SDCM, ICA_gauss, NNMF_negativesZeroed	[27 54 33]	1982.45	7.0E-3
8	SDCM, ICA_pow3, ICA_gauss	[27 130 54]	1982.70	6.2E-3
9	SDCM, ICA_gauss, FABIAS_RDefaults	[27 54 86]	1982.70	6.1E-3
10	SDCM, ICA_gauss, FABIAS_paperDefaults	[27 54 29]	1982.74	6.0E-3
11	SDCM, ICA_gauss, FABIA_RDefaults	[27 54 5]	1982.96	5.4E-3
12	SDCM, ICA_pow3, FABIAS_RDefaults	[27 463 8]	1983.30	4.6E-3
13	SDCM, SDCM, NNMF_negativesZeroed	[11 82 46]	1983.35	4.4E-3
14	SDCM, SDCM, ICA_gauss	[7 27 54]	1983.46	4.2E-3
15	SDCM, SDCM, ICA_gauss	[11 27 496]	1983.57	4.0E-3
16	ICA_gauss, FABIA_RDefaults, NNMF_exp_L2Rs	[54 86 26]	1984.54	2.5E-3
17	SDCM, NNMF_exp_L2Rs, NNMF_negativesZeroed	[12 26 8]	1984.58	2.4E-3
18	ICA_gauss, FABIA_RDefaults, FABIA_RDefaults	[54 3 54]	1984.66	2.3E-3
19	SDCM, ICA_tanh, ICA_gauss	[27 13 54]	1984.75	2.2E-3
20	ICA_tanh, ICA_gauss, FABIA_RDefaults	[13 54 91]	1984.93	2.0E-3
21	SDCM, SDCM, SDCM	[11 27 82]	1984.93	2.0E-3
22	SDCM, NNMF_exp_L2Rs, NNMF_negativesZeroed	[11 26 46]	1984.97	2.0E-3
23	SDCM, SDCM, PCA	[11 27 16]	1985.07	1.9E-3
24	SDCM, FABIAS_RDefaults, FABIAS_RDefaults	[27 8 32]	1985.26	1.7E-3
25	SDCM, SDCM, NNMF_exp_L2Rs	[11 27 26]	1985.34	1.6E-3

Supplementary References

1. Rosner, B. *Fundamentals of Biostatistics, 7th Edition*. (Brooks/Cole, Cengage Learning, Inc., 2011).
2. Hyvärinen, A. Fast and robust fixed-point algorithms for independent component analysis. *IEEE Trans. Neural Netw.* **10**, 626–34 (1999).
3. Hochreiter, S. *et al.* FABIA: factor analysis for bicluster acquisition. *Bioinformatics* **26**, 1520–7 (2010).
4. Edgar, R., Domrachev, M. & Lash, A. E. Gene Expression Omnibus: NCBI gene expression and hybridization array data repository. *Nucleic Acids Res.* **30**, 207–210 (2002).
5. Visco, C. *et al.* Comprehensive gene expression profiling and immunohistochemical studies support application of immunophenotypic algorithm for molecular subtype classification in diffuse large B-cell lymphoma. *Leukemia* **26**, 2103–13 (2012).
6. Subramanian, A. *et al.* Gene set enrichment analysis: a knowledge-based approach for interpreting genome-wide expression profiles. *Proc. Natl. Acad. Sci. USA* **102**, 15545–15550 (2005).
7. Lenz, G. *et al.* Stromal gene signatures in large-B-cell lymphomas. *N. Engl. J. Med.* **359**, 2313–23 (2008).
8. Reddy, A. *et al.* Genetic and Functional Drivers of Diffuse Large B Cell Lymphoma. *Cell* **171**, 481-494.e15 (2017).
9. O'Leary, N. A. *et al.* Reference sequence (RefSeq) database at NCBI: current status, taxonomic expansion, and functional annotation. *Nucleic Acids Res.* **44**, D733-45 (2016).
10. Langmead, B., Trapnell, C., Pop, M. & Salzberg, S. L. Ultrafast and memory-efficient alignment of short DNA sequences to the human genome. *Genome Biol.* **10**, R25 (2009).
11. Li, B. & Dewey, C. N. RSEM: accurate transcript quantification from RNA-Seq data with or without a reference genome. *BMC Bioinformatics* **12**, 323 (2011).
12. Cox, D. R. Regression models and life tables. *J. R. Stat. Soc. Ser. B* **34**, 187–220 (1972).
13. Burnham, K. P. & Anderson, D. R. *Model Selection and Multimodel Inference: a Practical Information - Theoretic Approach, second edition*. *Springer-Verlag, New York* (2002).
14. Shipp, M. A. *et al.* A Predictive Model for Aggressive Non-Hodgkin's Lymphoma. *N. Engl. J. Med.* **329**, 987–994 (1993).
15. Wilks, S. S. The Large-Sample Distribution of the Likelihood Ratio for Testing Composite Hypotheses. *Ann. Math. Stat.* **9**, 60–62 (1938).
