# Supplementary material for: Dissection of gene expression datasets into clinically relevant interaction signatures via high-dimensional correlation maximization
Source: Nat Commun. 2019 Nov 28;10:5417. doi: 10.1038/s41467-019-12713-5 (PMC6883077; doi:10.1038/s41467-019-12713-5)
Supplement: Supplementary file 3 — Description of Additional Supplementary Files [file 41467_2019_12713_MOESM3_ESM.pdf]

Description of Additional Supplementary Files

File Name: Supplementary Data 1

Description: **Discovered signatures in gene space.** This supplementary data table (in Microsoft® Excel® format) defines all 105 signatures detected for DLBCL gene expression data in the gene space with transcript cluster resolution (i.e., it contains final gene axes $|\mathbf{b}_{\hat{l}}\rangle$, correlations $|\mathbf{r}^{\mathrm{g}}\rangle$ to them, $|\mathbf{p}^{\mathrm{g}}\rangle$ values for correlations, gene weights $|\mathbf{w}^{\mathrm{g}}\rangle$ and gene strengths $|\mathbf{u}^{\mathrm{g}}\rangle$ for all nonzero gene weights).

File Name: Supplementary Data 2

Description: **Discovered signatures in sample space.** This supplementary data table (in Microsoft® Excel® format) defines all 105 signatures detected for DLBCL gene expression data in the sample space (i.e., it contains final sample axes $|\mathbf{b}_{\hat{l}}\rangle$, correlations $|\mathbf{r}^{\mathrm{s}}\rangle$ to them, $|\mathbf{p}^{\mathrm{s}}\rangle$ values for correlations, sample weights $|\mathbf{w}^{\mathrm{s}}\rangle$ and sample strengths $|\mathbf{u}^{\mathrm{s}}\rangle$ for all samples). Sample strengths $|\mathbf{u}^{\mathrm{Val,s}}\rangle$ of transferred signatures in the validation cohort are provided in the second worksheet.

File Name: Supplementary Data 3

Description: **Derived sets of top genes for discovered signatures.** Additionally, this supplementary data table (in Microsoft® Excel® format) provides derived traditional gene signatures (i.e. sets of top genes) for backwards compatibility (e.g., with Gene Set Enrichment Analysis).
