# Supplementary material for: Dissection of gene expression datasets into clinically relevant interaction signatures via high-dimensional correlation maximization
Source: Nat Commun. 2019 Nov 28;10:5417. doi: 10.1038/s41467-019-12713-5 (PMC6883077; doi:10.1038/s41467-019-12713-5)
Supplement: Supplementary file 7 — Reporting Summary [file 41467_2019_12713_MOESM7_ESM.pdf]

nature research

Corresponding author(s): Peter Lenz

Last updated by author(s): Aug 31, 2019

Reporting Summary

Nature Research wishes to improve the reproducibility of the work that we publish. This form provides structure for consistency and transparency in reporting. For further information on Nature Research policies, see Authors & Referees and the Editorial Policy Checklist .

Statistics

For all statistical analyses, confirm that the following items are present in the figure legend, table legend, main text, or Methods section.

n/a	Confirmed	
☐	☒	The exact sample size (*n*) for each experimental group/condition, given as a discrete number and unit of measurement
☒	☐	A statement on whether measurements were taken from distinct samples or whether the same sample was measured repeatedly
☐	☒	The statistical test(s) used AND whether they are one- or two-sided<br>*Only common tests should be described solely by name; describe more complex techniques in the Methods section.*
☐	☒	A description of all covariates tested
☐	☒	A description of any assumptions or corrections, such as tests of normality and adjustment for multiple comparisons
☐	☒	A full description of the statistical parameters including central tendency (e.g. means) or other basic estimates (e.g. regression coefficient) AND variation (e.g. standard deviation) or associated estimates of uncertainty (e.g. confidence intervals)
☐	☒	For null hypothesis testing, the test statistic (e.g. *F*, *t*, *r*) with confidence intervals, effect sizes, degrees of freedom and *P* value noted<br>*Give P values as exact values whenever suitable.*
☒	☐	For Bayesian analysis, information on the choice of priors and Markov chain Monte Carlo settings
☐	☒	For hierarchical and complex designs, identification of the appropriate level for tests and full reporting of outcomes
☐	☒	Estimates of effect sizes (e.g. Cohen's *d*, Pearson's *r*), indicating how they were calculated

Our web collection on statistics for biologists contains articles on many of the points above.

Software and code

Policy information about availability of computer code

Data collection	n/a (No new data was collected for this method study; see data availability statement below.)
Data analysis	As encouraged, we provide our MALTAB® analysis toolbox including the full SDCM source code, unit tests and examples at https://github.com/GrauLab/SDCM (first release commit is the paper version; repository will be made public in sync with paper release). We used MATLAB® (versions R2013b-R2016b, The MathWorks® Inc., Natick, Massachusetts, United States) for development and all computations.

For manuscripts utilizing custom algorithms or software that are central to the research but not yet described in published literature, software must be made available to editors/reviewers. We strongly encourage code deposition in a community repository (e.g. GitHub). See the Nature Research guidelines for submitting code & software for further information.

Data

Policy information about availability of data

All manuscripts must include a data availability statement. This statement should provide the following information, where applicable:

- Accession codes, unique identifiers, or web links for publicly available datasets
- A list of figures that have associated raw data
- A description of any restrictions on data availability

[copy of the data availability statement provided in the manuscript:]
Analyzed DLBCL GE datasets are publicly available in NCBI GEO via accessions GSE31312 for the detection cohort and GSE10846 for the validation cohort. Data for the RNA-sequencing based add-on validation cohort are available from the European Genome-Phenome Archive (https://www.ega-archive.org) via accession EGAS00001002606. Discovered DLBCL signatures and derived top gene sets are provided as Supplementary Datasets 1-3.

Field-specific reporting

Please select the one below that is the best fit for your research. If you are not sure, read the appropriate sections before making your selection.

☒ Life sciences ☐ Behavioural & social sciences ☐ Ecological, evolutionary & environmental sciences

For a reference copy of the document with all sections, see nature.com/documents/nr-reporting-summary-flat.pdf

Life sciences study design

All studies must disclose on these points even when the disclosure is negative.

Sample size	See 2.3 and 2.4 in Supplementary Methods for details. In brief, the detection cohort GSE31312 provided samples from n=498 DLBCL patients, the microarray-based validation cohort (GSE10846) for n=233, the RNA-sequencing based add-on validation cohort (EGAS00001002606) for n=624.
Data exclusions	same sample exclusions as by QC in original studies; no additional exclusions.
Replication	n/a (instead, our method actively searches for reproduced signatures in multiple samples from different patients)
Randomization	n/a
Blinding	n/a

Reporting for specific materials, systems and methods

We require information from authors about some types of materials, experimental systems and methods used in many studies. Here, indicate whether each material, system or method listed is relevant to your study. If you are not sure if a list item applies to your research, read the appropriate section before selecting a response.

Materials & experimental systems

n/a	Involved in the study	
☒	☐	Antibodies
☒	☐	Eukaryotic cell lines
☒	☐	Palaeontology
☒	☐	Animals and other organisms
☒	☐	Human research participants
☒	☐	Clinical data

Methods

n/a	Involved in the study	
☒	☐	ChIP-seq
☒	☐	Flow cytometry
☒	☐	MRI-based neuroimaging
